# Identification of Breast Cancer Subtype Specific MicroRNAs Using Survival Analysis to Find Their Role in Transcriptomic Regulation

**DOI:** 10.3389/fgene.2019.01047

**Published:** 2019-10-31

**Authors:** Michał Denkiewicz, Indrajit Saha, Somnath Rakshit, Jnanendra Prasad Sarkar, Dariusz Plewczynski

**Affiliations:** ^1^Laboratory of Functional and Structural Genomics, Center of New Technologies, University of Warsaw, Warsaw, Poland; ^2^College of Inter-Faculty Individual Studies in Mathematics and Natural Sciences, University of Warsaw, Warsaw, Poland; ^3^Department of Computer Science and Engineering, National Institute of Technical Teachers’ Training and Research, Kolkata, India; ^4^Cognitive and Analytics, Larsen & Toubro Infotech Ltd., Pune, India; ^5^Department of Computer Science & Engineering, Jadavpur University, Kolkata, India; ^6^Faculty of Mathematics and Information Science, Warsaw University of Technology Warsaw, Poland

**Keywords:** breast cancer, Kaplan-Meier estimator, miRNA-seq, Nelson-Aalen estimator, protein-protein interaction, regulatory circuit, survival analysis

## Abstract

The microRNA (miRNA) biomolecules have a significant role in the development of breast cancer, and their expression profiles are different in each subtype of breast cancer. Thus, our goal is to use the Next Generation Sequencing provided high-throughput miRNA expression and clinical data in an integrated fashion to perform survival analysis in order to identify breast cancer subtype specific miRNAs, and analyze associated genes and transcription factors. We select top 100 miRNAs for each of the four subtypes, based on the value of hazard ratio and p-value, thereafter, identify 44 miRNAs that are related to all four subtypes, which we call as four-star miRNAs. Moreover, 12, 14, 9, and 15 subtype specific, viz. one-star miRNAs, are also identified. The resulting miRNAs are validated by using machine learning methods to differentiate tumor cases from controls (for four-star miRNAs), and subtypes (for one-star miRNAs). The four-star miRNAs provide 95% average accuracy, while in case of one-star miRNAs 81% accuracy is achieved for HER2-Enriched. Differences in expression of miRNAs between cancer stages is also analyzed, and a subset of eight miRNAs is found, for which expression is increased in stage II relative to stage I, including hsa-miR-10b-5p, which contributes to breast cancer metastasis. Subsequently we prepare regulatory networks in order to identify the interactions among miRNAs, their targeted genes and transcription factors (TFs), that are targeting those miRNAs. In this way, key regulatory circuits are identified, where genes such as *TP53*, *ESR1*, *BRCA1*, *MYC*, and others, that are known to be important genetic factors for the cause of breast cancer, produce transcription factors that target the same genes as well as interact with the selected miRNAs. To provide further biological validation the Protein-Protein Interaction (PPI) networks are prepared and Kyoto Encyclopedia of Genes and Genomes pathway and gene ontology (GO) enrichment analysis are performed. Among the enriched pathways many are breast cancer-related, such as PI3K-Akt or p53 signaling pathways, and contain proteins such as TP53, also present in the regulatory networks. Moreover, we find that the genes are enriched in GO terms associated with breast cancer. Our results provide detailed analysis of selected miRNAs and their regulatory networks.

## Introduction

Breast cancer is the second most common type of cancer and a leading cause of death among women worldwide (Bray et al., 2018; Siegel et al., 2019). Due to its heterogeneous nature, different approaches have been used to classify its molecular subtypes (Dai et al., 2015). Recent research has shown that breast cancer can be categorized into four subtypes, based on the regulatory activity of molecular receptors like estrogen, progesterone and human epidermal growth factor receptor 2 (HER2) (Blenkiron et al., 2007; Banerji et al., 2012; Hon et al., 2016). The four subtypes are Luminal A, Luminal B, HER2-Enriched and Basal-Like. In the Luminal A (LA) subtype, estrogen and progesterone receptors are positive and HER2 receptor is negative. On the other hand, in Luminal B (LB) subtype, estrogen and progesterone receptors are positive, and HER2 receptor can be either positive or negative. In case of HER2-Enriched (HER2-E) subtype, HER2 receptor is positive and estrogen and progesterone receptors are negative. While in Basal-Like (BL) subtype, which is also known as triple-negative breast cancer, all three receptors are negative. The Luminal A (LA) breast cancer is the most prevalent, constituting 50–70% of female breast cancer cases (DeSantis et al., 2017; Kwan et al., 2009), while the latter three subtypes are less common, constituting approximately 10–20% of all cases for Luminal B and Basal-Like, and 5–20% for HER2-Enriched. The presence of one or more of these receptors suggests that a treatment targeting their pathways might be effective, thus the subtype identification and subtype-aware research are needed. For instance the HER2-Enriched subtype tumors can be often effectively treated with therapies aimed at the HER2 protein (Brand et al., 2006). The breast cancer subtypes also vary in their clinical characteristics. For example, HER2-Enriched cancers tend to grow faster than Luminal A or B cancers and the outlook is usually worse. Crucially, it has been found that each subtype possess different expression profiles of miRNAs (Sørlie et al., 2001; Hon et al., 2016).

MiRNAs are non-coding RNA molecules of length 19-22 nucleotides, first discovered in *Caenorhabditis elegans* (Lim et al., 2003). Subsequently a range of studies revealed their important cellular functions (Bartel, 2009; Wahid et al., 2010). They are responsible for post-transcriptional gene regulation: they bind with their targeted mRNAs and degrade them, and as a result those targeted mRNAs are not able to take part in protein formation. Over the years, researchers were trying to understand the involvement of miRNAs in different malignancies (Reddy, 2015; Peng and Croce, 2016; Hosseinahli et al., 2018) including, most importantly for this work, breast cancer (Takahashi et al., 2015; Kurozumi et al.,2017). The advancement of Next Generation Sequencing (NGS) techniques (van Dijk et al., 2014), such as miRNA-seq, provided the data for more extensive studies of miRNAs. Subsequently it has been show in many studies, that genetic components such as miRNAs play a significant role in development, growth and metastasis of breast cancer (Chang et al., 2016). In this regard, a review (Takahashi et al., 2015) was carried out to find the functions of miRNAs associated with breast cancer and discuss their potential clinical uses. On the other hand, Li et al. (2015) focused on the role that miRNAs play in breast cancer, as well as discussed their potential as prognostic and predictive biomarkers. Moreover, breast cancer associated miRNAs were identified in order to understand the impact of expression change in this cancer type (Kawaguchi et al., 2017). In Wang and Lou (2015), the importance of the oncogene and tumor suppressor miRNAs associated with breast cancer are studied and developments in therapies are discussed, Kurozumi et al. (2017) conducted a survey on the recent trends of miRNA in breast cancer, focused on the association of miRNAs in particular breast cancer subtypes. For this purpose, Oztemur Islakoglu et al. (2018) proposed an approach, in which multiple microarray datasets were used to obtain a set of subtype-specific miRNAs. Studies also propose machine learning methods: Sathipati and Ho (2018) provide a method based on support vector machines and feature selection to predict cancer stage. Finally, a review (Bahrami et al., 2018) focused on circulating and tissue-specific miRNAs as biomarkers.

One of the key methods in studying the role of miRNAs in breast cancer is survival analysis. Several tools have been developed to perform survival analysis, including Kaplan-Meier Plotter (Györffy et al., 2010), BreastMark (Madden et al., 2013) or SurvMicro (Aguirre-Gamboa and Trevino, 2014). These, however, can be used on microarray datasets, and do not make full use of the NGS data. Among the tools that use such data, are PROGgeneV2 (Goswami and Nakshatri, 2014), which focuses on genes, and PROGmiR (Goswami and Nakshatri, 2012) which allows for comparison of survival between high and low expression groups, but does not relay information about cancer subtypes. Finally, integrated solutions begin to emerge, like KM-express (Chen et al., 2018), that combines survival data as well as gene and cell line expression. Overall, majority of the tools and studies are still concerned about the functional and expression change of miRNAs associated with breast cancer, but the survival analysis of those miRNAs for breast cancer subtypes is limited to the best of our knowledge. Moreover, while studies employ survival analysis at the validation step, using survival data to identify candidate miRNAs is not common. This fact motivated us to conduct the present study on survival analysis using the NGS-provided high-throughput expression and clinical data of miRNAs associated with breast cancer subtypes.

To address the above fact, we propose a method of identifying miRNAs related to breast cancer and its intrinsic subtypes based on survial analysis. We use the Kaplan-Meier estimator (Jager et al., 2008; Hosmer Jr et al., 2013) and the log-rank test (Bland and Altman, 2004; Koletsi and Pandis, 2017) to rank the miRNAs by their influence to the patient survival in each breast cancer subtype. The results of the survival analysis provide us with four different sets of miRNAs with their rank based on the difference of hazard ratio (Sedgwick and Joekes, 2015), and p-value of the log-rank test. By selecting 100 miRNAs for each subtype and intersecting the lists, five different sets of miRNAs are prepared and named as four-star and one-star miRNAs. The four-star miRNAs are involved in all four subtypes, which means that this set of miRNAs can be used to discriminate the tumor and control samples of breast cancer. On the other hand, the four one-star miRNAs sets contain miRNAs specific to the subtypes such as Luminal A, Luminal B, HER2-Enriched and Basal-Like. These one-star miRNAs can be helpful for the identification of breast cancer subtypes.

In order to validate our findings computationally, we use seven well-known machine learning methods: Logistic Regression (LR, Hosmer Jr et al., 2013), Decision Tree (DT, Rokach and Maimon 2008), Artificial Neural Network (ANN), also known as Multilayer Perceptron (Huda et al., 2014), Support Vector Machine with linear kernel (SVM, Ben-Hur and Weston 2010), K-Nearest Neighbors (K-NN, Kramer 2013), Random Forest (RF, Qi 2012), and finally Naive Bayes Classifier (NB, Barber 2012). Using these methods we performed five 2-class classification tasks and obtained the average accuracy of 95%, 68%, 73%, 81% and 77% for four-star and one-star miRNAs, respectively. We also compared these results, with the accuracy obtained on the sets of miRNAs from other studies, where the four-star miRNAs identified by us provided superior performance on our expression dataset. Next, we investigate the changes in expression of the selected miRNAs in different stages of breast cancer. We identify 10 miRNAs that exhibit variable expression in cancer stages I and II, and for 8 of which there is an increase as the stage progresses. In order to perform biological validation we analyze the networks of miRNAs, genes targeted by those miRNAs, and transcription factors (TFs), that target the miRNAs. To do this, we gradually refine the sets of miRNAs, genes and TFs, incorporating mRNA expression data, to obtain a network of closely interacting miRNAs, genes, and TFs that are all associated with breast cancer. As a result, we find several regulatory circuits, in which a miRNA targets a gene, that produces a transcription factor, that targets the same miRNA. We also analyze protein-protein interaction networks for the refined set of transcription factors. Finally, Kyoto Encyclopedia of Genes and Genomes (KEGG) pathway and gene ontology (GO) enrichment analysis is performed, where we find cancer-related pathways and GO terms to be significantly enriched in genes targeted by the four-star and one-star miRNAs.

## Materials and Methods

This section describes briefly the Kaplan-Meier and Nelson-Aalen estimators, the Log-Rank Test, the preparation of the dataset and the proposed framework.

### Survival Analysis

One of the widely used non-parametric methods for analyzing survival is the Kaplan-Meier (KM) estimator (Clark et al., 2003; Jager et al., 2008; Stel et al., 2011). The KM estimator refers to a certain population and estimates the survival function *S*(*t*), describing the probability of a certain event (in our case, a patient’s death) happening before a certain point in time *t*, as given in the Equation 1.

(1) S(t)=P(T>t), 0<t<∞

where *T* is the random variable representing the time of death. A less steep shape of survival function means better prognosis for the population, for which the function has been estimated. The Kaplan-Merier estimator is defined as in Equation 2.

(2) $${\hat{S}}_{\mathrm{KM}}(t)=\underset{k:t_{k}\leq t}{\Pi }\left(1-\frac{E_{k}}{N_{k}}\right)$$

where, *t* *_k_* is the time when at least one event (i.e. death) occurred, *E* *_k_* is the number of events occurring at time *t* *_k,_* and the so-called “individuals known to survive” at *t* *_k_* (event of death not occurred, or right-censored) is expressed by *N* *_k_*. The expression data, along with the status and last days to follow up information, is used to compute the KM plots. The log-rank test is a non-parametric statistical test used to compare the survival curves of the two samples. The test statistic is described by Equation 3,

(3) $$Z_{i}=\frac{\overset{T}{\underset{k=1}{\Sigma }}(O_{ik}-E_{ik})}{\sqrt{\overset{T}{\underset{k=1}{\Sigma }}\text{Var}(O_{ik})}}$$

where *O* *_ik_* and *E* *_ik_* are the observed and expected numbers of events (deaths) in group *i* (one of the two) at time *k* = 1,…, *T*. The test statistic *Z* converges to the normal distribution as *T* approaches infinity. The null hypothesis is that there is no difference between the populations in the probability of an event at any time point. While the log-rank test provides a p-value for the difference between groups, it does not yield an estimate of the effect size. For this purpose, we compute the hazard ratio (HR): the ratio of the hazard rates (probabilities of death at a give time) in the two compared groups of patients. Taking our data as an example: if *HR* > 1, then the high expression group has a higher chance of dying at any given time point. Under the null hypothesis of the log-rank test, i.e. when no difference in the probability of death between the two groups, the hazard ratio is equal to 1.

For comparison, we also include another non-parametric method of analyzing survival, named as Nelson-Aalen estimator (Colosimo et al., 2002; Borgan, 2005). It estimates the cumulative hazard rate Λ(*t*), which is given in the Equation 4:

(4) Λ(t)=−log⁡S(t), 0<t<∞

The Nelson-Aalen estimator has the form given in Equation 5, with the similar symbols as mentioned in the Kaplan-Meier estimator.

(5) $${\hat{H}}_{\mathrm{NA}}(t)=\underset{k:t_{k}\leq t}{\Sigma }\frac{E_{k}}{N_{k}}$$

### Data Preparation

The expression and clinical data of miRNA-seq of breast invasive carcinoma (BRCA) is obtained from The Cancer Genome Atlas (TCGA Network, 2012). TCGA provides miRNA-seq data in the form of reads per million (RPM) of 842 patients. However, the cancer subtype information is provided only for 231 patients: 190 with breast cancer and 41 controls. For this dataset, the subtype categorization based on Prediction Analysis of Microarray 50 genes (PAM 50) is taken (Ohnstad et al., 2017). The statistics for each breast cancer subtype are provided in **Table 1**. With this expression data, we have the clinical information such as age, gender, last day to followup and status (alive or dead). Moreover, we select only those miRNAs for which at least 60% expression values are non-zero.

**Table 1:** Statistics of four breast cancer subtypes and control samples.

Subtype	Code	Sample size	Average age	Average days to last follow-up
Luminal A	LA	86	56.96	1704.55
Luminal B	LB	39	55.07	1431.86
HER2-Enriched	HER2-E	24	52.87	1307.12
Basal-Like	BL	41	56.41	1402.34
Control	Control	41	54.73	1632.60

### Method

**Figure 1:**
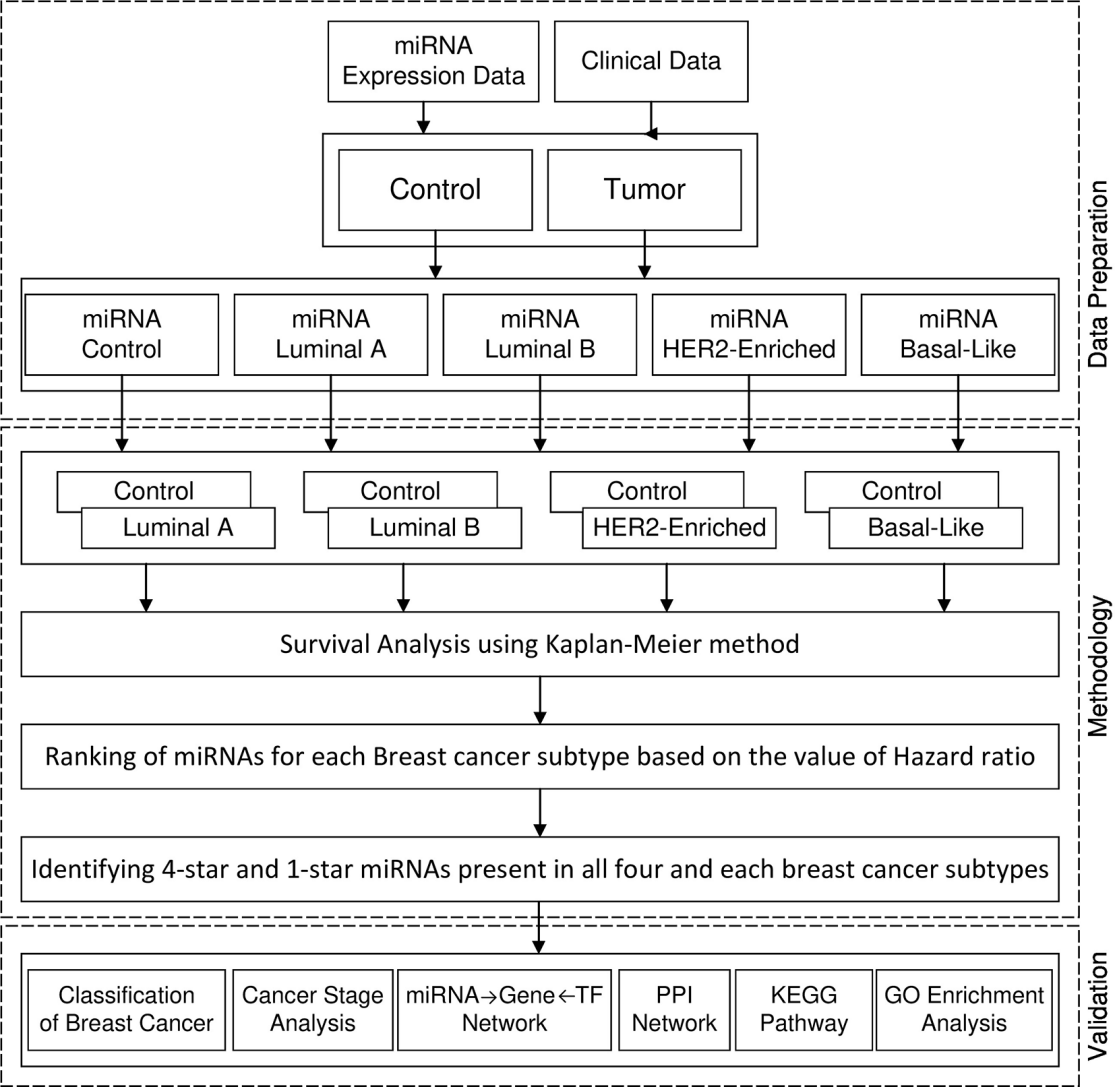
Framework of proposed method.

**Figure 1** shows the framework of our method in detail. First, we perform survival analysis using the KM method separately for each cancer subtype. For each subtype of breast cancer, we combine the set of patients having the expression of miRNA in cancer subtype with the controls. With such data we enter to the survival analysis, where patients are split into two balanced groups, based on the high-expression (above median) and low-expression (below median) of miRNA. For these two groups, we use KM estimator and log-rank test to obtain the hazard ratio (HR) and p-value. Using these statistics we create four ranking lists of miRNAs (one for each breast cancer subtype), based on the difference of hazard ratio and p-value, from highest to lowest. We then select 100 top miRNAs from each of the four subtype-related lists. In this way, we obtain filtered lists of miRNAs, each of which is most related to one of the breast cancer subtypes. A given miRNA can be present in any of these four lists related to the subtypes. In this study we focus on sets of miRNAs obtained by analyzing the intersection of the four lists. These are: the four-star miRNAs which are present in all lists, and one-star miRNAs, that are present only in a single list. The four-star miRNAs are considered to be relevant in all subtypes, while the one-star miRNAs are subtype-specific.

Finally, in order to rank the four-star miRNAs, we use an objective function Ψ, which provides a measure of relevance incorporating both statistical significance in terms of p-value and strength of influence of miRNA to the subtypes, i.e. hazard ratio. The objective function Ψ is defined in Equation 6 for each miRNA as sum of the difference of hazard ratio and p-value over the four subtypes.

(6) $$\Psi =\overset{S}{\underset{i=1}{\Sigma }}(HR_{i}-p-\text{valu}e_{i})$$

where *HR* *_i_* and *p-value* *_i_* are hazard ratios and p-values obtained for a given miRNA for the *i*-th cancer subtype and *S* is the number of subtypes i.e. equal to 4.

For the computational verification of the miRNAs, we use seven popular machine learning methods (Logistic Regression, Decision Tree, Artificial Neural Network, Support Vector Machine, K-Nearest Neighbour, Random Forest and Gaussian Naive Bayes Classifier) to assess the relevance of the four-star and one-star miRNAs in predicting breast cancer and distinguishing its type. As an input for the classifiers, we use the expression datasets of patients, in order to perform two different experiments. First, for the four-star miRNA set, we perform binary classification, by differentiating between tumor vs. control cases, without the division into different breast cancer subtypes. This is because the four-star miRNAs are related to all subtypes. Then, for each set of one-star miRNAs, we train the classifiers to distinguish the patients with the particular cancer subtype vs. all other patients (those with other breast cancer subtypes and controls). Moreover, we compare the accuracy of the above machine learning methods on our four-star miRNA set, to other sets of miRNAs proposed in the literature as either biomarkers or otherwise associated with breast cancer. The analysis is conducted in exactly the same fashion as with our miRNAs, as described above. Only those sets of miRNAs proposed by other studies are used, for which expression data existed in the TCGA dataset. These studies are listed along with the results.

## Results and Discussion

In this section, we first describe the results of miRNA selection procedure based on survival analysis, through which we obtain the four-star and one-star miRNAs. Next, we discuss the accuracy of classifiers that distinguish cancer patients from controls, as well as differentiate the breast cancer subtypes, based on the miRNAs expression data. Finally, we validate biological significance of the five miRNA sets identified by our method.

In order to validate our results, we perform the following analyses: first, we use miRTarBase (Chou et al., 2018) for finding miRNA-targeted genes, TransmiR v2.0 (Tong et al., 2019) for finding transcription factors targeting the miRNAs, and TRRUST v2 (Han et al., 2018) for transcription factor’s targeted genes. This data, after refinement, is used to create regulatory networks of miRNAs, genes and TFs. Next, we create protein-protein interaction (PPI) networks of the transcription factors targeting the miRNAs, and identified highly connected nodes, that are important in the network. For this purpose, the STRING database (Szklarczyk et al., 2018) is used. Thereafter, we perform Kyoto Encyclopedia of Genes and Genomes (KEGG, Kanehisa and Goto 2000) pathway analysis, in order to find pathways influenced by the miRNAs’ targeted genes. Finally, Gene Ontology enrichment analysis is performed, to find the biological processes, cellular components, and molecular functions on which the miRNAs have influence through their targeted genes. Both pathway and gene ontology analysis is performed using STRING database as well.

### Selection of miRNAs Using Survival Analysis

**Figure 2:**
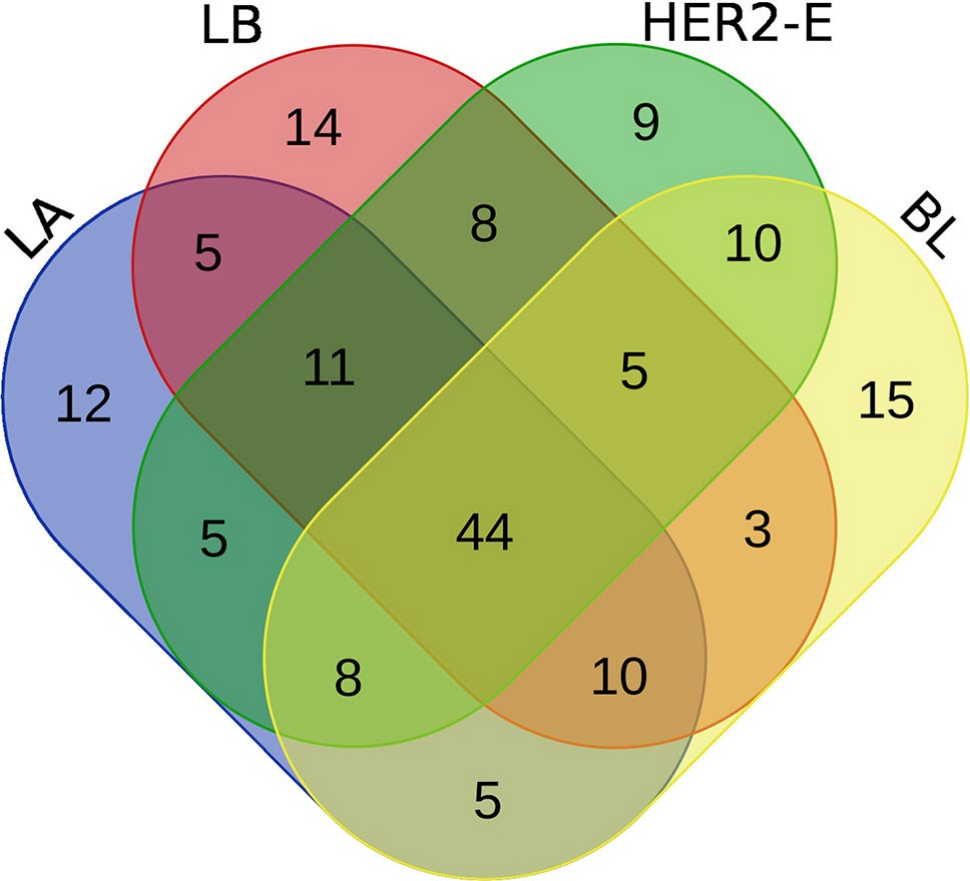
Venn diagram of top 100 miRNAs for four breast cancer subtypes.

We perform the survival analysis using MATLAB software (MATLAB, 2018). From the initial set of patients, we retain only those, for whom two conditions are met: 1) the breast cancer subtype information is known and 2) at least 60% of the expression values are non-zero. Using these criteria, 190 patients are entered into the analysis, forming four groups for the breast cancer subtype, as described in the Method section. Each group is divided into high and low expression subgroups by median split, and hazard ratio is computed between the low and high expression groups. The log-rank test comparing the two groups is also performed to obtain a p-value. Moreover, the survival functions of the high and low-expression groups are estimated using the KM estimator, and survival plots are created. Then, four rankings (one for each breast cancer subtype) of the initial 587 miRNAs are created, based on difference of the hazard ratio and p-value relevant to the given subtype, and the top 100 miRNAs are taken from each list. We inspect, how these lists of 100 miRNAs intersect, since a given miRNA can be present in more than one list, and the intersection is visualized using a Venn diagram in **Figure 2**.

We note the 44 miRNAs present in all four lists as constituting the set of four-star miRNAs. Subtype-specific sets (miRNAs present in exactly one of the lists) are also identified, comprising 12 miRNAs for the LA subtype, 14 for LB subtype, 9 for the HER2-E subtype, and finally 15 for the BL subtype. The results of survival analysis of four-star miRNAs are presented in **Table 2**. Apart from the value of the previously described scoring function, the hazard ratios and corresponding p-values for each of the cancer subtype are reported in the same table.

**Table 2:** The four-star miRNAs ranked based on the sum (Ψ) of the difference of hazard ratio and p-value over the four breast cancer subtypes, along with the PubMed ID.

miRNA	LA		LB		HER2-E		BL		Ψ Score	PubMed ID
	HR	p-value	HR	p-value	HR	p-value	HR	p-value		
hsa-miR-365a-3p	3.308	0.001	2.467	0.044	3.503	0.007	4.851	0.000	14.076	23592263
hsa-miR-452-5p	2.311	0.019	3.039	0.012	4.922	0.000	2.367	0.065	12.542	23592263
hsa-miR-378a-3p	2.318	0.019	2.910	0.017	3.003	0.020	4.037	0.002	12.210	23592263
hsa-miR-215-5p	2.127	0.039	2.636	0.037	2.158	0.101	4.655	0.001	11.399	23592263
hsa-miR-103a-3p	3.664	0.000	2.182	0.086	3.713	0.004	1.572	0.376	10.664	21572407
hsa-miR-224-5p	3.193	0.001	3.435	0.005	2.372	0.061	1.095	0.996	9.031	23399735
hsa-miR-335-3p	2.203	0.035	2.119	0.100	4.142	0.002	2.317	0.068	10.576	23592263
hsa-miR-326	2.661	0.010	2.081	0.110	2.455	0.057	3.061	0.014	10.066	20216554
hsa-miR-10a-5p	1.344	0.471	1.934	0.157	2.217	0.110	4.203	0.001	8.958	23622248
hsa-miR-217	1.848	0.092	4.166	0.001	1.985	0.162	1.346	0.610	8.479	19008416
hsa-miR-10b-3p	2.091	0.054	2.098	0.112	1.521	0.443	3.878	0.003	8.976	22012620
hsa-miR-378a-5p	2.791	0.004	1.560	0.395	2.076	0.135	2.996	0.017	8.874	23592263
hsa-miR-193a-5p	2.372	0.016	1.803	0.215	3.078	0.016	1.922	0.172	8.757	23446348
hsa-miR-664a-3p	1.967	0.066	1.411	0.527	1.470	0.494	3.456	0.006	7.210	23592263
hsa-miR-30c-2-3p	2.731	0.006	1.847	0.208	2.197	0.118	2.093	0.117	8.419	23592263
hsa-miR-511-5p	2.245	0.030	2.316	0.064	1.956	0.176	2.366	0.064	8.549	23592263
hsa-miR-143-3p	2.400	0.014	1.841	0.206	1.157	0.901	2.567	0.042	6.802	17504027
hsa-miR-10b-5p	1.524	0.272	2.304	0.069	2.374	0.079	2.445	0.056	8.171	17898713
hsa-miR-22-3p	2.148	0.038	2.715	0.025	1.099	0.992	1.786	0.237	6.456	20371350
hsa-miR-140-3p	1.769	0.141	2.083	0.118	2.270	0.090	2.481	0.051	8.203	23446348
hsa-miR-338-3p	2.806	0.005	1.644	0.313	1.885	0.181	1.164	0.879	6.121	25945841
hsa-miR-451a	2.035	0.050	1.610	0.330	1.639	0.340	2.812	0.022	7.354	20227367
hsa-miR-486-5p	2.263	0.027	1.380	0.554	1.565	0.401	2.603	0.035	6.793	23592263
hsa-miR-28-3p	1.966	0.063	2.363	0.063	2.169	0.101	1.499	0.451	7.318	23592263
hsa-miR-139-5p	1.709	0.167	1.685	0.293	1.791	0.253	2.641	0.033	7.080	21925125
hsa-miR-125b-2-3p	1.869	0.089	1.821	0.223	0.986	0.850	2.683	0.034	6.162	23592263
hsa-miR-100-5p	1.868	0.089	1.811	0.235	1.771	0.280	2.311	0.086	7.071	19739117
hsa-miR-195-5p	1.547	0.253	1.351	0.608	1.430	0.550	2.642	0.036	5.524	18320040
hsa-miR-584-5p	1.623	0.200	1.812	0.218	2.546	0.053	1.442	0.512	6.442	23446348
hsa-let-7c-5p	1.835	0.099	1.821	0.223	1.118	0.972	2.224	0.094	5.610	17600087
hsa-miR-574-3p	2.434	0.022	1.330	0.624	1.489	0.467	1.304	0.665	4.780	23592263
hsa-miR-144-5p	2.184	0.032	1.239	0.747	1.387	0.574	1.822	0.214	5.065	26458302
hsa-miR-145-5p	1.696	0.158	1.446	0.497	1.296	0.701	2.191	0.098	5.175	20160723
hsa-let-7e-3p	2.092	0.054	1.159	0.875	1.460	0.502	1.491	0.449	4.322	24398324
hsa-miR-24-1-5p	1.443	0.348	2.057	0.123	1.045	0.915	1.900	0.191	4.868	23592263
hsa-miR-30a-3p	1.364	0.466	1.258	0.720	1.815	0.256	1.902	0.180	4.717	23592263
hsa-miR-362-5p	1.584	0.222	2.059	0.117	1.646	0.333	1.316	0.657	5.277	24495516
hsa-miR-339-5p	1.333	0.509	1.303	0.653	1.716	0.285	1.089	0.985	3.010	23592263
hsa-miR-361-3p	1.992	0.071	1.485	0.445	1.381	0.584	0.933	0.967	3.723	23592263
hsa-miR-30e-3p	1.518	0.280	1.748	0.253	1.327	0.656	1.076	0.977	3.504	23592263
hsa-miR-145-3p	1.684	0.164	1.435	0.506	1.264	0.743	1.760	0.257	4.473	24398324
hsa-miR-29a-3p	1.526	0.271	1.718	0.261	1.401	0.561	1.080	0.980	3.651	19850741
hsa-miR-34a-5p	1.653	0.178	1.830	0.197	1.603	0.360	1.403	0.539	5.216	18834855
hsa-miR-193b-5p	1.373	0.454	1.034	0.900	1.785	0.242	1.022	0.876	2.742	23446348

**Figure 3:**
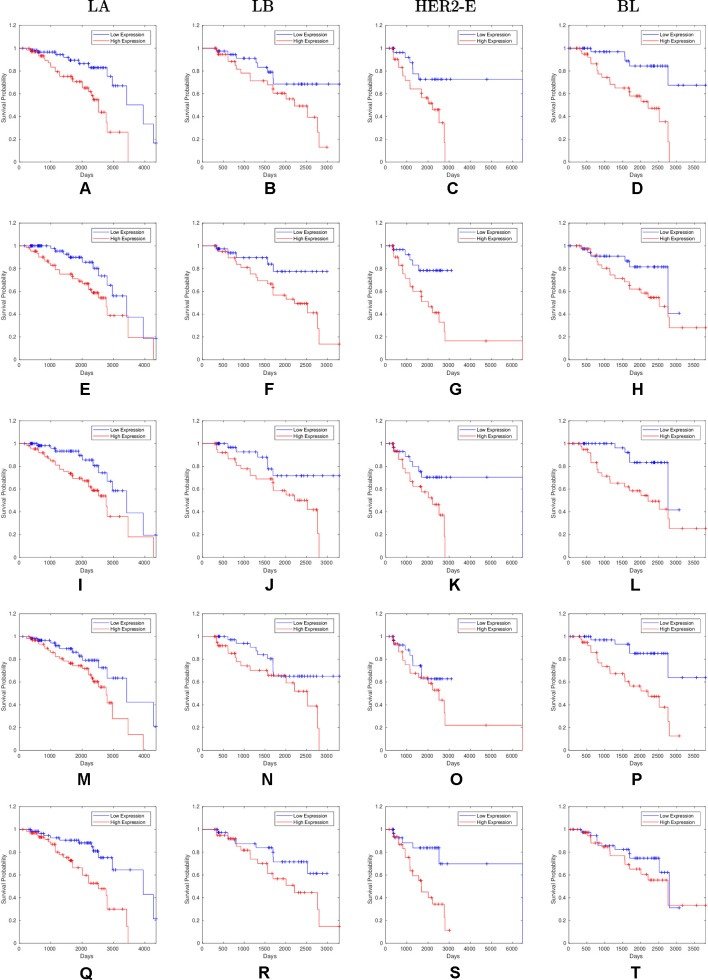
Survival plots of top five four-star miRNAs: **(A**–**D)**:hsa-miR-365a-3p, **(E**–**H)**:hsa-miR-452-5p, **(I**–**L)**:hsa-miR-378a-3p, **(M**–**P)**:hsa-miR-215-5p and **(Q**–**T)**:hsa-miR-103a-3p as mentioned in **Table 2** where blue line indicates low expression group and red line indicates high expression group.

**Figure 4:**
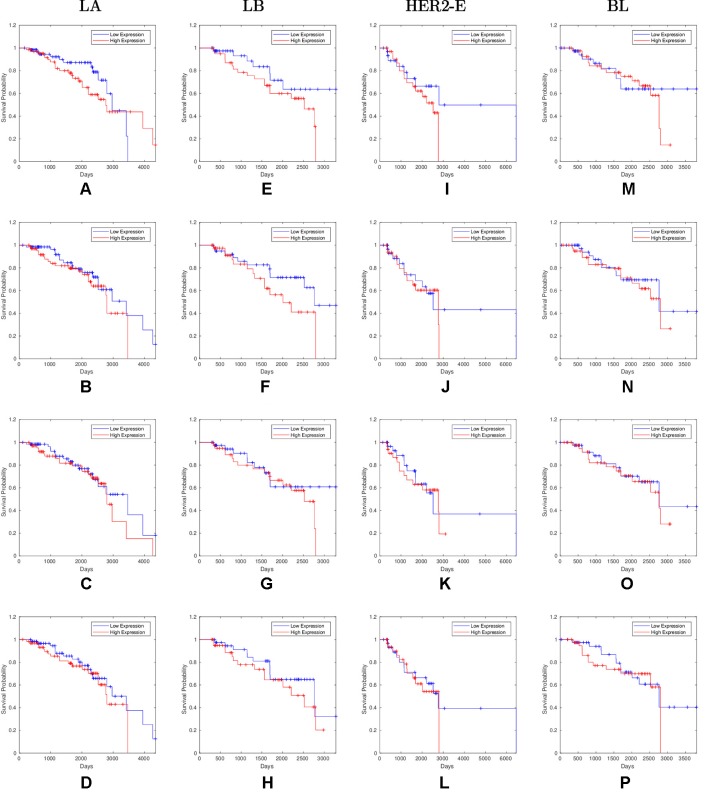
Survival plots of top four one-star miRNAs for LA:**(A)** hsa-miR-30b-3p **(B)** hsa-miR-1247-5p **(C)** hsa-miR-221-3p **(D)** hsa-miR-186-5p LB:**(E)** hsa-miR-485-3p **(F)** hsa-miR-582-3p **(G)** hsa-miR-491-5p **(H)** hsa-miR-22-5p HER2-E:**(I)** hsa-miR-30c-5p **(J)** hsa-miR-26b-3p **(K)** hsa-miR-29c-5p **(L)** hsa-miR-19b-3p BL:**(M)** hsa-miR-199b-5p **(N)** hsa-miR-889-3p **(O)** hsa-miR-26b-5p **(P)** hsa-miR-214-3p subtypes as mentioned in **Table 3** where blue line indicates low expression group and red line indicates high expression group.

Overall, 26 out of 44 miRNAs are significant (*p-value* < 0.05) after log-rank test for at least one cancer subtype. In all except one case there is a negative relation between survivability and the expression of the miRNA (*HR* > 1). The exception was hsa-miR-361-3p for HER2-E subtype, where the *HR* = 0.933 and p-value = 0.967. Since the sample is not very large, and we were mainly interested in ranking based on hazard ratio, and not in the influence per se, we do not reject any miRNAs on the base of p-value, and proceed with biological validation. The Kaplan-Meier survival plots of top five miRNAs from the list of four-star miRNAs are shown in **Figure 3** for each subtype. Similarly, survial plots of top four one-star miRNAs for each subtype are presented in **Figure 4**, and the obtained statistics in **Table 3**. Both the Kaplan-Meier plots and the cumulative hazard ratio plots, created using Nelson-Aalen estimator, indicate in concordance, that increased expression increases breast cancer risk. The hazard ratio and p-value obtained from the log-rank test for 100 miRNAs of each subtype are reported in the **Supplementary Table 1**. Moreover, both Kaplan-Meier survival plots and cumulative hazard ratio plots using Nelson-Aalen estimator are provided in **Supplementary Figures S1**–**S10**.

**Table 3:** The one-star miRNAs ranked based on the difference of hazard ratio and p-value for four breast cancer subtypes.

miRNA	LA			miRNA	LB		
	HR	p-value	Ψ Score		HR	p-value	Ψ Score
hsa-miR-30b-3p	1.461	0.354	1.107	hsa-miR-485-3p	2.073	0.116	1.956
hsa-miR-1247-5p	1.458	0.352	1.105	hsa-miR-582-3p	1.892	0.179	1.713
hsa-miR-221-3p	1.373	0.437	0.936	hsa-miR-491-5p	1.603	0.348	1.255
hsa-miR-222-3p	1.363	0.478	0.885	hsa-miR-22-5p	1.588	0.352	1.236
hsa-miR-1180-3p	1.362	0.477	0.885	hsa-miR-1249-3p	1.531	0.401	1.131
hsa-miR-186-5p	1.332	0.505	0.827	hsa-miR-146b-3p	1.288	0.681	0.608
hsa-miR-107	1.331	0.509	0.823	hsa-miR-374b-5p	1.260	0.713	0.546
hsa-miR-505-3p	1.317	0.530	0.787	hsa-miR-151a-3p	1.209	0.795	0.414
hsa-miR-455-5p	1.308	0.534	0.774	hsa-miR-152-3p	1.004	0.844	0.159
hsa-miR-532-5p	1.221	0.689	0.532	hsa-miR-27b-5p	1.020	0.877	0.143
hsa-miR-502-3p	1.217	0.689	0.528	hsa-miR-181a-3p	1.019	0.876	0.143
hsa-miR-24-3p	1.208	0.689	0.519	hsa-miR-185-5p	1.027	0.889	0.138
				hsa-miR-1307-3p	1.054	0.940	0.114
				hsa-miR-425-3p	0.939	0.957	-0.019
miRNA	HER2-E			miRNA	BL		
	HR	p-value	Ψ Score		HR	p-value	ΨScore
hsa-miR-30c-5p	1.746	0.295	1.451	hsa-miR-199b-5p	1.508	0.447	1.061
hsa-miR-26b-3p	1.175	0.867	0.308	hsa-miR-889-3p	1.240	0.753	0.486
hsa-miR-29c-5p	1.163	0.883	0.281	hsa-miR-26b-5p	1.230	0.769	0.461
hsa-miR-19b-3p	1.128	0.941	0.187	hsa-miR-214-3p	1.145	0.907	0.238
hsa-miR-20b-5p	1.019	0.871	0.148	hsa-miR-136-3p	0.999	0.836	0.163
hsa-miR-874-3p	1.038	0.900	0.138	hsa-miR-134-5p	1.011	0.856	0.156
hsa-miR-3127-5p	1.038	0.901	0.136	hsa-miR-101-5p	1.022	0.873	0.148
hsa-miR-299-5p	1.059	0.941	0.118	hsa-miR-30b-5p	0.992	0.844	0.148
hsa-miR-20a-3p	1.068	0.956	0.111	hsa-miR-542-3p	1.028	0.888	0.140
				hsa-miR-154-5p	1.060	0.947	0.114
				hsa-miR-375	1.066	0.958	0.108
				hsa-miR-409-5p	0.971	0.893	0.078
				hsa-miR-409-3p	0.929	0.972	-0.043
				hsa-miR-539-5p	0.925	0.984	-0.059
				hsa-let-7f-5p	0.920	0.993	-0.073

### Selected miRNAs for Classification of Breast Cancer

Using seven machine learning methods viz. Logistic Regression, Decision Tree, Artificial Neural Network, Support Vector Machine, K-Nearest Neighbour, Random Forest and Gaussian Naive Bayes Classifier, we perform five binary classification tasks by considering four-star and one-star miRNAs for which the results are presented in **Table 4**. The implementations of the algorithms is provided by the scikit-learn Python library version 0.19.1 (Pedregosa et al., 2011), and the detailed parameters of each algorithm are reported in the **Supplementary Table 2**, by following the literature (Pedregosa et al., 2011). For each breast cancer subtype, the machine learning methods are able to distinguish patients with that subtype from other breast cancer subtypes and controls, using the expression data of the relevant sets of four-star and one-star miRNAs, by achieving average accuracy of 95%, 68%, 73%, 81%, and 77% using 5-fold cross-validation. Among the seven machine learning methods, Random Forest achieves on average the highest accuracy of 84% over five sets of miRNAs. Moreover, the four-star set of miRNAs provides a higher average accuracy of the classifiers, than the sets that have been obtained from the literature, using the same expression data. Here 7 studies have been considered, and average accuracy (listed in **Table 4**) ranges from 77% to 94% on tumor vs control classification task.

**Table 4:** Classification results of breast cancer using four-star miRNAs in comparison with other sets of miRNAs in the literature, as well as the results of one-star miRNAs. The four-star results are sorted according to average prediction accuracy. From other studies only those four-star miRNAs, for which expression data was available, are included.

miRNA set	No. of Set of miRNA	LR	DT	ANN	SVM	K-NN	RF	NB	Average
4-star (Our study)	44	0.965	0.914	0.935	0.922	0.978	0.965	0.974	0.951
[55]	23	0.927	0.922	0.927	0.952	0.974	0.948	0.897	0.935
[9]	12	0.901	0.901	0.909	0.923	0.957	0.953	0.953	0.928
[64]	3	0.888	0.918	0.883	0.888	0.905	0.922	0.931	0.905
[71]	2	0.827	0.836	0.836	0.814	0.862	0.844	0.818	0.834
[2]	3	0.780	0.806	0.858	0.780	0.871	0.832	0.801	0.818
[44]	2	0.706	0.762	0.823	0.693	0.823	0.805	0.849	0.780
[77]	3	0.732	0.749	0.809	0.719	0.806	0.789	0.788	0.770
1-star LA	12	0.663	0.667	0.711	0.597	0.710	0.710	0.676	0.676
1-star LB	14	0.577	0.745	0.775	0.635	0.805	0.810	0.732	0.726
1-star HER2-E	9	0.649	0.827	0.736	0.771	0.896	0.901	0.858	0.805
1-star BL	15	0.701	0.792	0.762	0.740	0.809	0.827	0.731	0.766

Furthermore, the hierarchical clustering analysis has been performed, in the same schema as in the classification task, to visualize the comparison between miRNA expression in cancer patients and controls for the four-star miRNAs and between the cancer subtype and control for one-star miRNAs. The resulting heatmaps are provided in the **Supplementary Figures 11** and **12**.

### Selected miRNAs for the Analysis of Cancer Stage

We analyze the relationship between miRNA expression and the stage of breast cancer, using the R statistical package (R Core Team, 2019). We assign each patient to one of breast cancer stages (I-IV), according to the AJCC stage data obtained from the TCGA database. Moreover, we restrict the analyses to stages I and II, as it is more clinically relevant to detect cancer in earlier stages. For the one-star miRNAs, only patients having that particular cancer subtype are taken, while for the four-star miRNAs all patients, for whom stage data were available, are included. The statistics of the samples of breast cancer stage for different subtypes are presented in the **Supplementary Table 3**.

**Figure 5:**
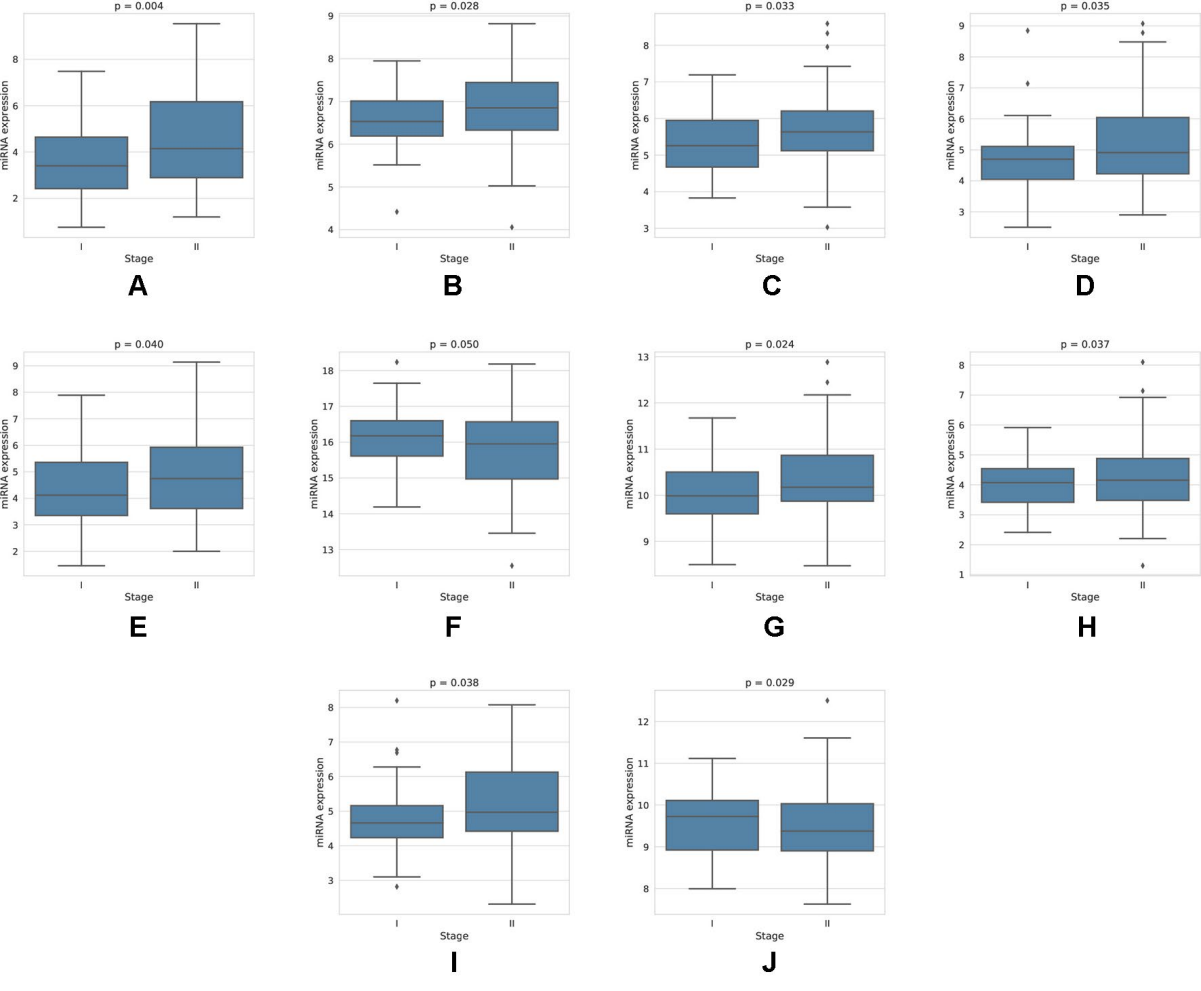
Expression of miRNAs in cancer stages I and II, for those 10 miRNAs, that have shown significant difference in expression. For the four-star miRNAs, these are: **(A)** hsa-miR-224-5p, **(B)** hsa-miR-574-3p, **(C)** hsa-miR-339-5p, **(D)** hsa-miR-584-5p, **(E)** hsa-miR-452-5p, **(F)** hsa-miR-10b-5p; For LA one-star miRNA set: **(G)** hsa-miR-24-3p, **(H)** hsa-miR-455-5p, **(I)** hsa-miR-505-3p; For HER-2: **(J)** hsa-miR-30c-5p

For each miRNA, we execute a one-way ANOVA, with the independent variable being the stage of cancer, and the dependent variable being the expression value of the miRNA. We find, among the four-star and one-star miRNA sets, 10 miRNAs with significant (*p-value* < 0.05) expression change between the stages. The four-star miRNAs identified are: hsa-miR-224-5p (p-value = 0.004), hsa-miR-574-3p (p-value = 0.028), hsa-miR-339-5p (p-value = 0.033), hsa-miR-584-5p (p-value = 0.035), hsa-miR-452-5p (p-value = 0.040) and hsa-miR-10b-5p (p-value = 0.050). Moreover, in the Luminal A one-star miRNA set, hsa-miR-24-3p (p-value = 0.024), hsa-miR-455-5p (p-value = 0.037) and hsa-miR-505-3p (p-value = 0.038) are having significant p-values. Finally, in the HER2-Enriched one-star miRNA set, the hsa-miR-30c-5p (p-value = 0.029) is significant. For all except hsa-miR-30c-5p and hsa-miR-10b-5p miRNAs, the expression increases with stage. This increase is concordant with the increased hazard in the group with high expression of these miRNAs, as indicated by the survival analysis, and implies, that the miRNA expression increases with stage progression. For example, overexpression of hsa-miR-10b-5p is known to trigger invasion and metastasis of breast cancer (Ma et al., 2007), which can be inhibited by silencing this miRNA (Ma et al., 2010) in a mouse model. Different survival characteristics of high and low expression groups can also induce changes in stage expression of miRNAs, as patients with high hazard value progress more rapidly through stages of cancer. On the other hand, an expression change in a given cancer stage can indicate a potential miRNA, that is associated specifically with a given stage. The plots showing the miRNA expression in different cancer stages are given in **Figure 5**. The full results of ANOVA are available in the **Supplementary Table 4**, as well as plots for all miRNAs in four-star and one-star sets are given in **Supplementary Figure 15**.

### Selected miRNAs for Regulatory Network Analysis

**Figure 6:**
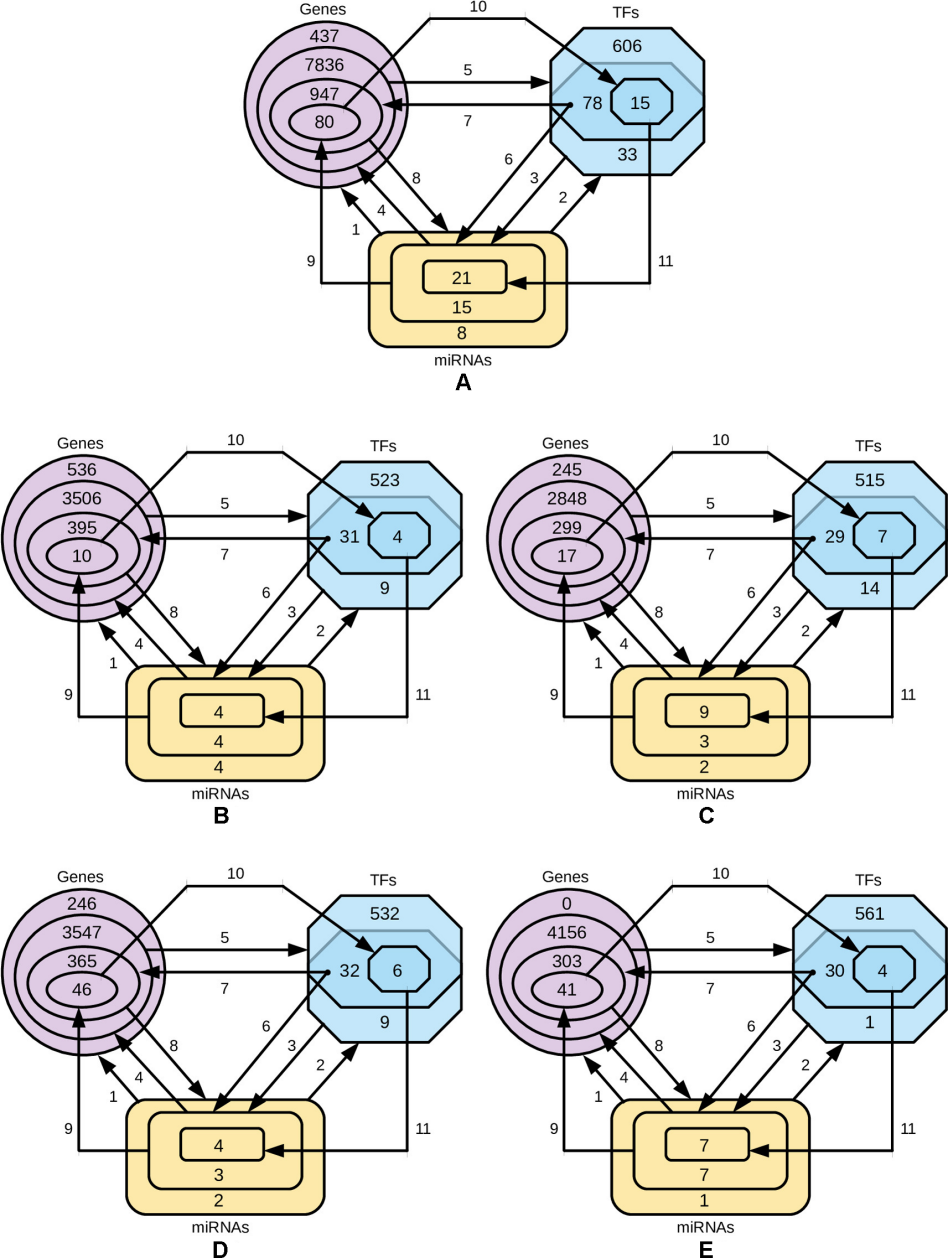
Diagrams representing the steps of refining the miRNAs, gene and TF sets. The steps 1-8 are, while the steps 9, 10 and 11 are based on negative correlation. **(A)** four-star miRNAs and one-star miRNAs for **(B)** LA, **(C)** LB, **(D)** HER2-E and **(E)** BL.

In order to identify the genes that are targeted by miRNAs and TFs, and the TFs that target miRNAs for four-star and one-star miRNAs, we used recently published databases of miRTarBase, TRRUST and TransmiR respectively. Then we refine these three sets to select only those genes, TFs and miRNAs, that are closely related. The procedure is described below and visualized in **Figure 6**.

- Step 1: for the miRNAs in a given set (either four-star or one of the one-star) we identify the targeted genes.
- Step 2: the TFs that target the miRNAs in the set are identified.
- Step 3: for the obtained TFs we identify a smaller subset of targeted miRNAs.
- Step 4: the genes targeted by this smaller subset of miRNAs is selected.
- Step 5: the set of TFs associated with the genes identified in step 4.
- Step 6: an intersection of the two sets of TFs (produced by steps 2 and 5) is computed. These TFs from both these sets target the initial miRNAs.
- Step 7: we find the genes targeted by TFs from step 6.
- Step 8: genes, in turn, are targeted by the initial set of miRNAs.
- Step 9: the expression values of miRNAs are correlated with the mRNA expression, and only those mRNAs are selected, for which Pearson correlation is below −0.3.
- Step 10: the TFs targeting genes corresponding to the mRNAs are found.
- Step 11: the miRNA set is restricted by considering only the miRNAs targeted by TFs identified in the previous step.

The numbers of miRNAs, genes and TFs in each phase of the procedure is provided in **Supplementary Table 5**, and the details of each step, in the **Supplementary Table 6**.

**Figure 7:**
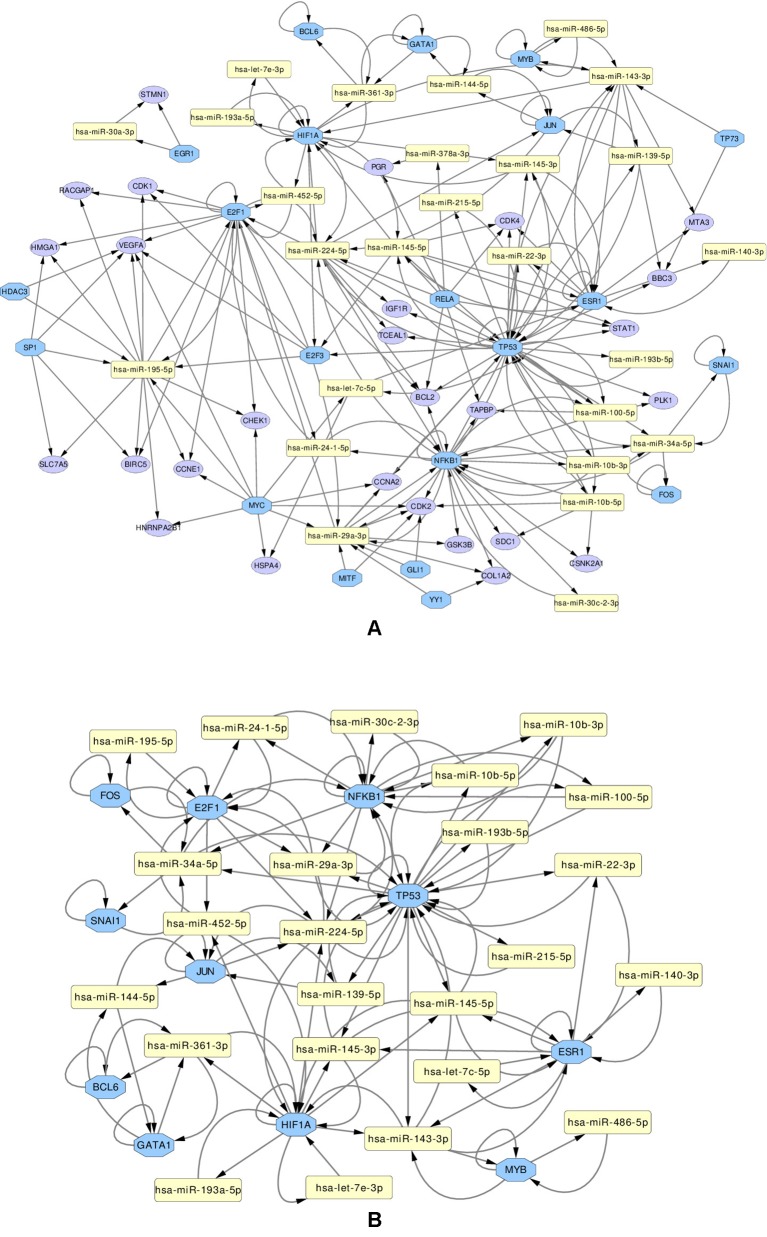
Regulatory networks of miRNAs (yellow rectangle), genes (purple ellipse) and transcription factors (blue octagon), created using the combined list of transcription factors. The network **(A)** contains all elements. Network **(B)** contians only the loops between miRNAs and same-named Genes and TFs

We use these final, refined sets to create regulatory miRNA-Gene-TF networks, that show the relationships among the miRNAs, genes and transcription factors. These networks are given in **Supplementary Figures 13** and **14**. All such networks have been created for each miRNA set. However in this article, a combined network of miRNA-Gene-TF is shown in **Figure 7**. This combined network shows all the miRNAs, genes, and TFs present in other five networks. For better visualization, each network is provided in two forms: in one, all the nodes (miRNAs, genes, TFs) are present, in the other, only those nodes are presented, that form loops, where the TF and a gene with the same name are both associated with the same miRNA.

Several important transcription factors are present in the combined networks, such as the MYC proto-oncogene protein, which is acting as an oncogene for breast cancer (Dang, 2012), or the estrogen receptor 1 (ESR1), highly relevant to breast cancer (Clatot et al., 2017). Moreover, the breast cancer type 1 susceptibility protein (BRCA1), which acts as a tumor suppressor trough DNA repair (Friedenson, 2007) is present. Another node is the hypoxia-inducible factor 1-alpha encoded by HIF1A gene, whose abnormal expression is widely known to be involved in multiple cancers (Yeo et al., 2004). On the other hand, the nuclear factor NF kappa B encoded by NFKB1 gene, suspected but not associated directly with breast cancer (Curran et al., 2002), is also present and interacting with other elements in the network, which may indicate an indirect relationship with this disease. The E2F1 and E2F3 transcription factors, implicated in breast cancer and other cancer types, are also present: E2F1 is promoting cancer cell proliferation (Berteaux et al., 2005), while overexpression of E2F3 can lead to development of cancer (Vimala et al., 2012).

**Figure 8:**
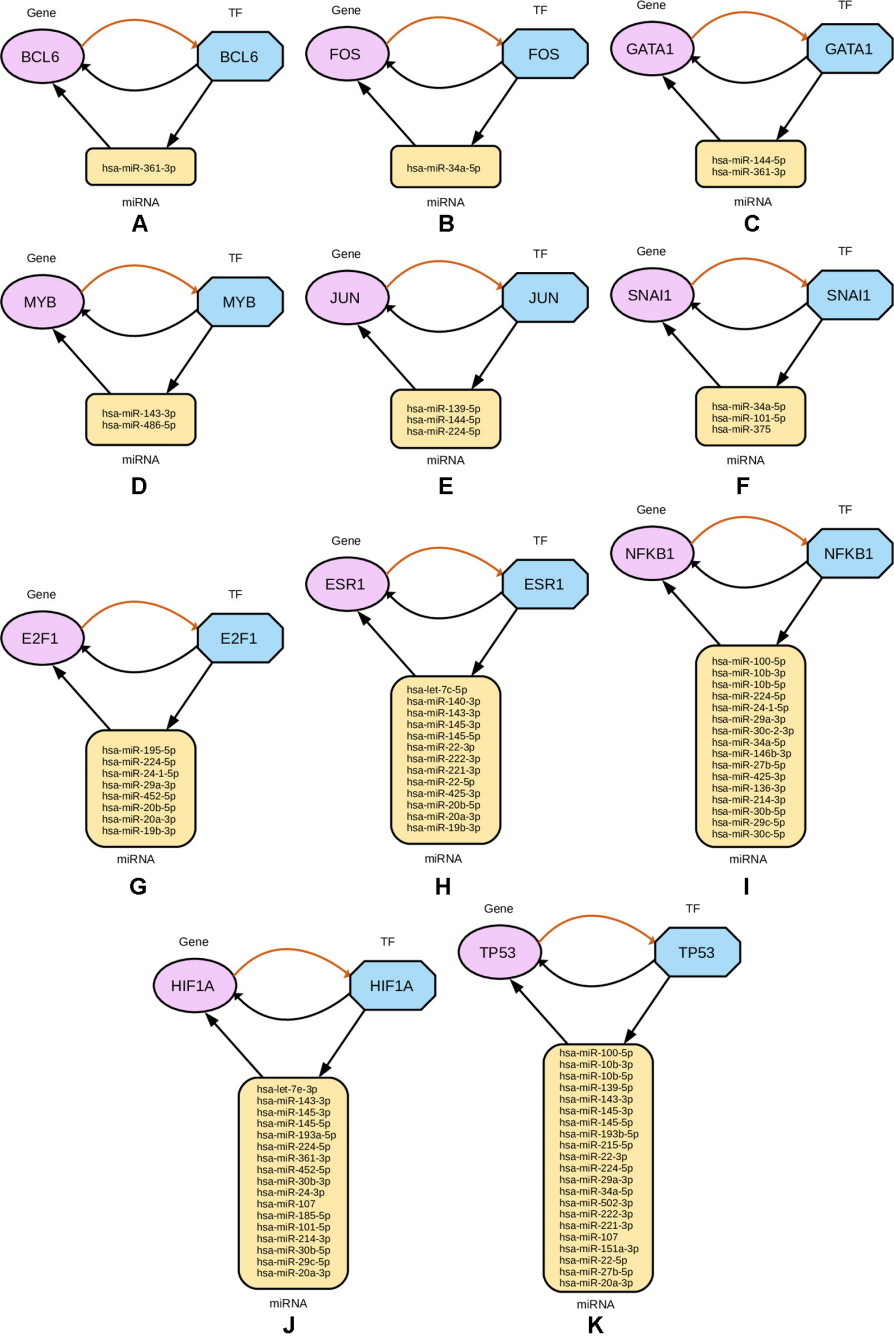
Regulatory circuits identified from cross-referencing miRNA and TF targets for the four-star and one-star miRNAs. In each subfigure, a TF targets a miRNA, that in turn targets a Gene associated with that TF. Multiple miRNAs are combined in the plot for the same TF and gene. The miRNAs are represented by yellow rounded rectangles, genes by purple ellipses, and transcription factors by blue octagons.

Subsequently, from the interaction results of miRNA-Gene-TF networks, we identify a set of regulatory circuits, where a certain miRNA targets a gene, and is targeted by a TF which is a product of the same gene and interacting with each other. For example, hsa-miR-100-5p targets *TP53* gene, which encodes the cellular tumor antigen p53, which is a tumor suppressor and alterations of which is associated with breast cancer (Børresen-Dale, 2003; Bourdon et al., 2005). These circuits are shown in **Figure 8**.

### Selected miRNAs for Protein-Protein Interaction Network Analysis

**Figure 9:**
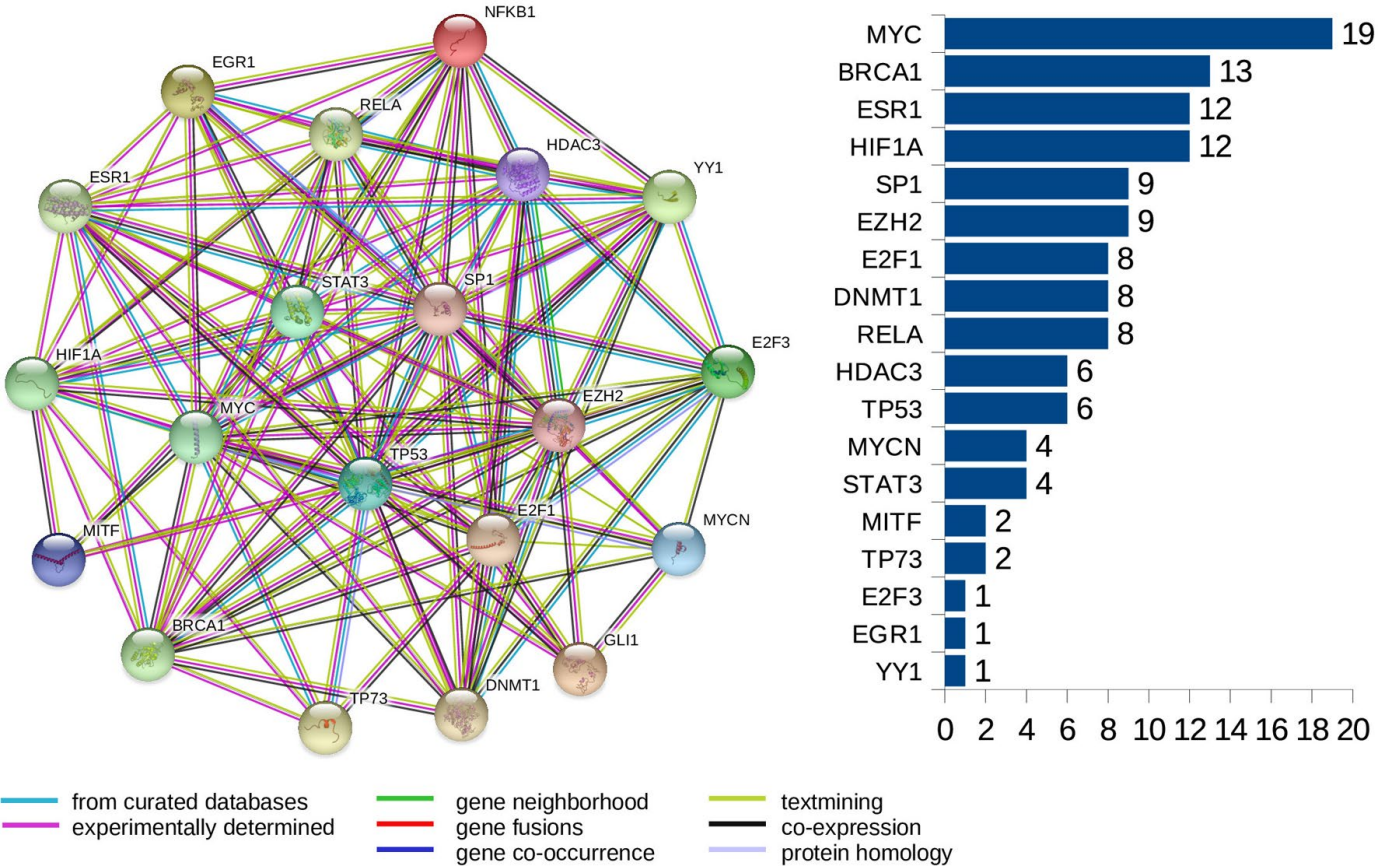
The PPI network created for the combined set of transcription factors. The barplot shows the number of connections for the proteins. Colors of edges represent protein-protein association type, as indicated in the legend. Node colors are for aesthetic purposes.

Furthermore, we look at protein-protein interaction (PPI) networks of the refined sets of proteins (TFs) that interact with the four-star and one-star miRNAs. Using STRING database we create six networks: one for each of the five sets of miRNA, and a combined network, using the transcription factors present in all of the five networks. All networks have highly significant enrichment p-values, indicating that significantly more interactions are present, than expected. The p-values are: 0.037 for Luminal A network, 5.88*e*−5 for Luminal B, 8.12*e*−5 for HER2-Enriched, 0.012 for Basal-Like, and less than 1.00*e*−16 for both the four-star network and the combined network. The combined network is presented in **Figure 9**, while the individual networks are shown in **Supplementary Figure 16**. From each PPI network, the degree of every node (number of interactions of a given protein) is computed, and shown alongside each network in respective figure. Here, our focus is to identify proteins forming hubs in the PPI networks, i.e. those having relatively large number of interactions. These highly connected proteins can be seen as significant, as cancer-related proteins tend to have more interaction partners and be located close to central hubs (Jonsson and Bates, 2006). They often are the very transcription factors discussed in the previous section. It is to be noted that several proteins with high number of interactions have been found in the combined network, starting with the protein MYC, being the most interacting for the combined network, as well as for the networks corresponding to the four-star miRNA set, and the one-star LB, HER2-E, and BL sets. Next, the BRCA1 protein is present and highly connected in the combined network and the LB network. Moreover, the estrogen receptor 1 (ESR1) is also present an highly connected in the combined network, as well as in HER2-E and BL network. The most interacting for the Luminal A breast cancer subtype, is the HIF1A protein, also mentioned in the discussion of the regulatory networks. Finally, one moderately connected protein not present in the combined regulatory network is the Polycomb group protein EZH2, identified as a marker of aggressive breast cancer (Kleer et al., 2003). The composition of the PPI networks is given in **Supplementary Table 7**, and the details of PPI interactions are in the **Supplementary Table 8**.

### Selected miRNAs for KEGG Pathway Analysis

For four-star and one-star miRNAs, we performed a KEGG pathway analysis using STRING database online tool, in order to uncover the pathways affected. The analysis has been performed for the refined sets of genes, associated with each of the miRNA sets. All five sets of genes are found to be enriched in pathways relating to microRNAs in cancer (hsa05206). Similarly, all cases except Luminal A were enriched in breast cancer (hsa05224) and pathways in cancer (hsa05200). Moreover, in all except HER2-Enriched subtype, apoptosis (hsa04210) is enriched (Musgrove and Sutherland, 2009). Many cancer-related pathways are also enriched in most of the five sets. These are for example the PI3K-Akt signaling pathway (hsa04151), which plays a significant role in tumor proliferation and endocrine resistance in breast cancer (Paplomata and O’Regan, 2014; Raphael et al., 2018), or the well studied p53 pathway [hsa04115, Sherr and McCormick (2002)]. Another example is the MAPK signaling pathway (hsa04010), aberrations in which are present in many tumor types (Downward, 2003), especially including the Basal-Like breast cancer (Giltnane and Balko, 2014). Significant pathways related to breast cancer for the four-star and one-star miRNAs are presented in **Table 5**. Full information about all enriched pathways is provided in **Supplementary Table 9**.

**Table 5:** KEGG pathways significantly enriched in the refined gene set.

ID	Description	4-star	LA	LB	HER2-E	BL
hsa05224	Breast cancer	✓		✓	✓	✓
hsa05200	Pathways in cancer	✓		✓	✓	✓
hsa05206	MicroRNAs in cancer	✓	✓	✓	✓	✓
hsa05202	Transcriptional misregulation in cancer	✓		✓		
hsa04210	Apoptosis	✓	✓	✓		✓
hsa04215	Apoptosis - multiple species	✓	✓		✓	✓
hsa04115	p53 signaling pathway	✓		✓	✓	✓
hsa04151	PI3K-Akt signaling pathway	✓		✓	✓	✓
hsa04010	MAPK signaling pathway	✓		✓		✓
hsa04110	Cell cycle	✓		✓	✓	✓

### Selected miRNAs for Gene Ontology Enrichment Analysis

Finally, we analyzed the GO annotations of targeted genes of four-star and one-star miRNAs, also using STRING database. Significantly enriched terms, related to breast cancer, are presented in **Table 6**, and the full results of GO enrichment analysis are available in the **Supplementary Table 10**. Most importantly, among the enriched biological processes are regulation of cell cycle (GO:0051173) and regulation of cell population proliferation (GO:0010604), that are linked to cancer (Yuan et al., 2015). These are enriched in the four-star miRNAs and in one-star HER2-E and BL sets, the latter also in the LB set. Among the molecular functions obtained from GO enrichment analysis are chromatin binding (GO:0003682) and protein binding (GO:0005515), significant in the four-star miRNAs, and LB, HER2-E and BL sets. In the one-star LA miRNAs, on the other hand, (GO:0003677) DNA binding and (GO:0019901) protein kinase binding are significantly enriched, both also enriched in the four-star miRNAs. For the four-star miRNAs, most notable significantly enriched cellular components include nucleus (GO:0005634), chromatin (GO:0000785) and heterochromatin (GO:0000792). In the case of one-star miRNAs the nucleus (GO:0005634) was also significantly enriched in all sets, except LA.

**Table 6:** Gene Ontology terms significantly enriched in the refined gene set.

ID	Description	4-star	LA	LB	HER2-E	BL
GO: Biological Process						
GO:0051173	Regulation of cell cycle	✓			✓	✓
GO:0010604	Regulation of cell population proliferation	✓		✓	✓	✓
GO:0000122	Negative regulation of transcription by RNA polymerase II	✓	✓	✓	✓	
GO:0010332	Response to gamma radiation	✓				
GO:0045892	Negative regulation of transcription, DNA-templated	✓			✓	✓
GO:0006974	Cellular response to DNA damage stimulus	✓	✓	✓		✓
GO:1902043	Positive regulation of extrinsic apoptotic signaling pathway via death domain receptors				✓	
GO: Molecular Function						
GO:0003682	Chromatin binding	✓		✓	✓	✓
GO:0005515	Protein binding	✓		✓	✓	✓
GO:0003677	DNA binding	✓	✓		✓	
GO:0019901	Protein kinase binding	✓	✓			✓
GO:0019900	Kinase binding	✓				
GO:0008139	Nuclear localization sequence binding	✓				
GO:0043565	Sequence-specific DNA binding	✓		✓	✓	
GO: Cellular Component						
GO:0044454	Nuclear chromosome part	✓				✓
GO:0005634	Nucleus	✓		✓	✓	✓
GO:0000785	Chromatin	✓			✓	
GO:0000792	Heterochromatin	✓			✓	
GO:0005737	Cytoplasm	✓			✓	✓
GO:0000803	Sex chromosome	✓				
GO:0005829	Cytosol	✓			✓	✓

## Conclusion

In this article, we proposed a method allowing to investigate the miRNAs related to breast cancer, based on Kaplan-Meier survival analysis and expression data obtained from sequencing of miRNAs. This method provides a subset of miRNAs specific to breast cancer subtypes. In particular, using our method, we have identified miRNAs for which increased expression decreases the odds of survival across all subtypes (four-star), and those related in this way only to one subtype (one-star). With the use of four-star miRNAs, we are able to classify patients into tumor and control by achieving 95% average accuracy, while the one-star miRNAs provide maximum of 81% average accuracy for subtype identification over seven machine learning methods. Moreover, the miRNAs described in the stage expression analysis can be the focus of future studies concerning cancer stage progression and its mechanisms. Additionally, investigating the miRNA-Gene-TF networks allowed us to identify miRNAs involved in regulatory circuits, where a miRNA targets and is being targeted by a certain Gene-TF pair. Moreover, PPI network analysis shows known cancer-related proteins, such as TP53, ESR1, BRCA1, MYC and others, which are encoded by genes targeted by some of the four-star and one-star miRNAs, adding to the validity of our study. Furthermore, KEGG pathway and GO enrichment analyses confirmed the biological relevance of our four-star and one-star miRNAs by showing that several pathways, processes and functions known to be associated with breast cancer as their targeted genes are enriched.

The outcome of this research can help to advance the understanding of miRNAs involvement in breast cancer and its subtypes. The key miRNAs can be further tested for use as biomarkers, which are crucial for the development of breast cancer detection and treatment response in the clinical setting (Gasparri et al., 2018). They can also potentially serve as targets for therapeutic modulation, which is a promising application of miRNAs (Mehrgou and Akouchekian, 2017).

## Author Contributions

MD, IS, and DP have conceived and designed the experiments. MD, IS, SR, and JS have performed the experiments. MD, IS, and SR have scripted the manuscript. MD, IS, and DP have corrected and edited the manuscript. All authors read and approved the final manuscript.

## Funding

This work has been supported by Polish National Science Centre (2014/15/B/ST6/05082), Foundation for Polish Science co-financed by the European Union under the European Regional Development Fund (TEAM to DP), and by the grant from Department of Science and Technology, Govt. of India and Polish Government under Indo-Polish/Polish-Indo project No.: DST/INT/POL/P-36/2016. The work was partially supported as RENOIR Project by the European Union Horizon 2020 research and innovation programme under the Marie Skłodowska-Curie grant agreement No 691152 and by Ministry of Science and Higher Education (Poland), grant Nos. W34/H2020/2016, 329025/PnH/2016.

## Conflict of Interest

Author JPS was employed by company Larsen & Toubro Infotech Ltd.

The remaining authors declare that the research was conducted in the absence of any commercial or financial relationships that could be construed as a potential conflict of interest.
