# Corrigendum: Identification of Breast Cancer Subtype Specific MicroRNAs Using Survival Analysis to Find Their Role in Transcriptomic Regulation

**DOI:** 10.3389/fgene.2019.01382

**Published:** 2020-02-10

**Authors:** Michał Denkiewicz, Indrajit Saha, Somnath Rakshit, Jnanendra Prasad Sarkar, Dariusz Plewczynski

**Affiliations:** ^1^Laboratory of Functional and Structural Genomics, Center of New Technologies, University of Warsaw, Warsaw, Poland; ^2^College of Inter-Faculty Individual Studies in Mathematics and Natural Sciences, University of Warsaw, Warsaw, Poland; ^3^Department of Computer Science and Engineering, National Institute of Technical Teachers’ Training and Research, Kolkata, India; ^4^Cognitive and Analytics, Larsen & Toubro Infotech Ltd., Pune, India; ^5^Department of Computer Science & Engineering, Jadavpur University, Kolkata, India; ^6^Faculty of Mathematics and Information Science, Warsaw University of Technology, Warsaw, Poland

**Keywords:** breast cancer, KaplanMeier estimator, miRNA-seq, Nelson-Aalen estimator, protein-protein interaction, regulatory circuit, survival analysis

In the original article, there are errors in the reference list the reference. The authors cited a few old versions of literature even though the latest version were consulted in the study.

The Reference for “MATLAB, 2010” was incorrectly written as “MATLAB (2010). version 7.10.0 (R2010a). Natick, Massachusetts: The MathWorks Inc.”. It should be “MATLAB (2018). version 9.4.0 (R2018a). Natick, Massachusetts: The MathWorks Inc.”.

The reference for “Chou et al., 2016” was incorrectly written as “Chou, C.-H., Chang, N.-W., Shrestha, S., Hsu, S.-D., Lin, Y.-L., Lee, W.-H., et al. (2016). miRTarBase 2016: updates to the experimentally validated miRNA-target interactions database. *Nucleic Acids Res*. 44, D239–D247. doi: 10.1093/nar/gkv1258”. It should be “Chou, C. H., Shrestha, S., Yang, C. D., Chang. N. W., Lin, Y. L., Liao, K. W., et al. (2018). miRTarBase update 2018: a resource for experimentally validated microRNA-target interactions. *Nucleic Acids Res*. 46 (D1), D296–D302. doi: 10.1093/nar/gkx1067”.

The reference for “Wang et al., 2010” was incorrectly written as “Wang, J., Lu, M., Qiu, C., and Cui, Q. (2010). TransmiR: a transcription factor-microRNA regulation database. Nucleic Acids Res. 38, D119 -D122. doi: 10.1093/nar/gkp803”. It should be “Tong, Z., Cui, Q., Wang, J., and Zhou,Y. (2019). TransmiR v2.0: an updated transcription factor-microRNA regulation database. *Nucleic Acids Res*. 47 (D1), D253 -D258. doi: 10.1093/nar/gky1023”.

The reference for “Han et al., 2015” was incorrectly written as “Han, H., Shim, H., Shin, D., Shim, J. E., Ko, Y., Shin, J., et al. (2015). TRRUST: a reference database of human transcriptional regulatory interactions. Sci. Rep. 5, 11432. doi: 10.1038/srep11432”. It should be “Han, H., Cho, J. W., Lee, S., Yun, A., Kim, H., Bae, D., et al. (2018). TRRUST v2: an expanded reference database of human and mouse transcriptional regulatory interactions. *Nucleic Acids Res*. 46 (D1), D380–D386. doi: 10.1093/nar/gkx1013”

The authors apologize for these errors and state that this does not change the scientific conclusions of the article in any way. The original article has been updated.

