# Supplementary material for: Identification of Breast Cancer Subtype Specific MicroRNAs Using Survival Analysis to Find Their Role in Transcriptomic Regulation
Source: Front Genet. 2019 Oct 31;10:1047. doi: 10.3389/fgene.2019.01047 (PMC6837165; doi:10.3389/fgene.2019.01047)
Supplement: Supplementary file 1 [file DataSheet_1.pdf]

Supplementary Material: Integration of miRNA-seq and Clinical data to Identify Breast Cancer Subtype specific miRNAs using Survival Analysis and their Interactions with Genes and Transcription Factors

1 SUPPLEMENTARY TABLES AND FIGURES

1.1 Tables

Table S1. List of top 100 miRNAs for each breast cancer subtype after performing survival analysis. (Please see Excel file named 'Top100-miRNAs')

Table S2. The parameters of seven different classifiers

Logistic Regression		Support Vector Machine		Decision Tree	
Parameter	Value	Parameter	Value	Parameter	Value
class_weight	balanced	class_weight	balanced	class_weight	balanced
solver	lbfgs				
max_iter	200	Random Forest		Multilayer Perceptron	
multi_class	multinomial	Parameter	Value	Parameter	Value
		class_weight	balanced	max_iter	1000
KNN				solver	lbfgs
Parameter	Value	Naive Bayes		early_stopping	True
n_neighbors	3	Parameter	Value		
		var_smoothing	10^-9		

Table S3. Statistics of the samples of breast cancer stage for different subtypes. For the 1-star miRNAs only the patients with respective cancer subtype are used, while in case of the 4-star miRNAs all patients with stage data are used

	Breast cancer stage			
miRNA set	I	II	III	IV
4-star	46	107	29	2
1-star LA	30	41	12	1
1-star LB	5	25	8	1
1-star H2	3	13	6	0
1-star BL	8	28	3	0

Table S4. Results of Anova test on the stage-wise data for 4-star and 1-star miRNAs (Please see Excel file named 'Anova-Stage')

Table S5. Count of miRNAs, genes, and transcription factors (TFs) after specific steps of the filtering procedure

miRNAs	Steps	miRNA	Gene	TF
4-star	1, 2	44	9300	126
	3, 4, 5	36	8863	699
	6, 7, 8	36	1027	93
	9, 10, 11	21	80	15
1-star LA	1, 2	12	4447	558
	3, 4, 5	8	3911	44
	6, 7, 8	8	405	35
	9, 10, 11	4	10	4
1-star LB	1, 2	14	3409	551
	3, 4, 5	12	3164	50
	6, 7, 8	12	316	36
	9, 10, 11	9	17	7
1-star HER2-E	1, 2	9	4204	570
	3, 4, 5	7	3958	47
	6, 7, 8	7	411	38
	9, 10, 11	4	46	6
1-star BL	1, 2	15	4500	595
	3, 4, 5	14	4500	35
	6, 7, 8	14	344	34
	9, 10, 11	7	41	4

Table S6. The detailed results of interactions of miRNA→Gene, TF→Gene, and TF→miRNA for 4-star and 1-star miRNAsm as well as for the combined networks

miRNA set	File
Combined network	Excel file named 'Combined_miRNA_Query_details'
4-star	Excel file named '44_miRNA_Query_details'
1-star LA	Excel file named 'LA_miRNA_Query_details'
1-star LB	Excel file named 'LB_miRNA_Query_details'
1-star HER2-E	Excel file named 'H2_miRNA_Query_details'
1-star BL	Excel file named 'BL_miRNA_Query_details'

Table S7. The Transcription factors identified as the result of filtering procedure, in each for 4-star and 1-star miRNA sets, and the common set (Please see the Excel file named 'TF-Common')

Table S8. The detailed results of PPI interaction analysis for 4-star and 1-star miRNAs (Please see the Excel file named 'PPI-Interaction')

Table S9. The detailed results of KEGG Pathway Analysis, from gene perspective, for 4-star and 1-star miRNAs (Please see the Excel file named 'KEGG')

Table S10. The detailed results of GO Enrichment Analysis, from gene perspective, for 4-star and 1-star miRNAs

miRNAs	File
4-star	Excel file named 'GO-44'
1-star LA	Excel file named 'GO-LA'
1-star LB	Excel file named 'GO-LB'
1-star HER2-E	Excel file named 'GO-HER2-E'
1-star BL	Excel file named 'GO-BL'

1.2 Figures

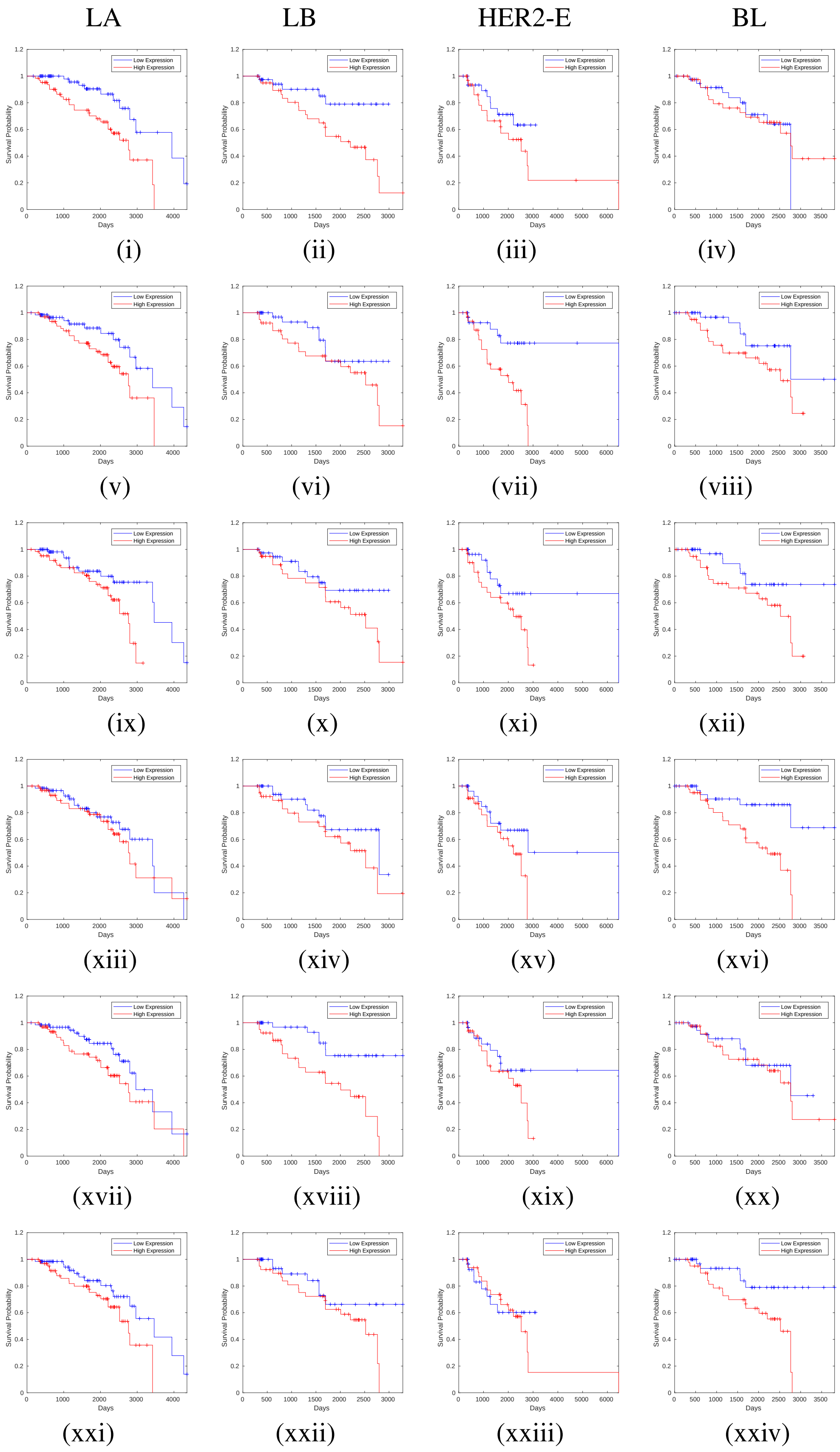
LA
LB
HER2-E
BL
Low Expression
High Expression
Survival Probability
Days
(i)
(ii)
(iii)
(iv)
(v)
(vi)
(vii)
(viii)
(ix)
(x)
(xi)
(xii)
(xiii)
(xiv)
(xv)
(xvi)
(xvii)
(xviii)
(xix)
(xx)
(xxi)
(xxii)
(xxiii)
(xxiv)

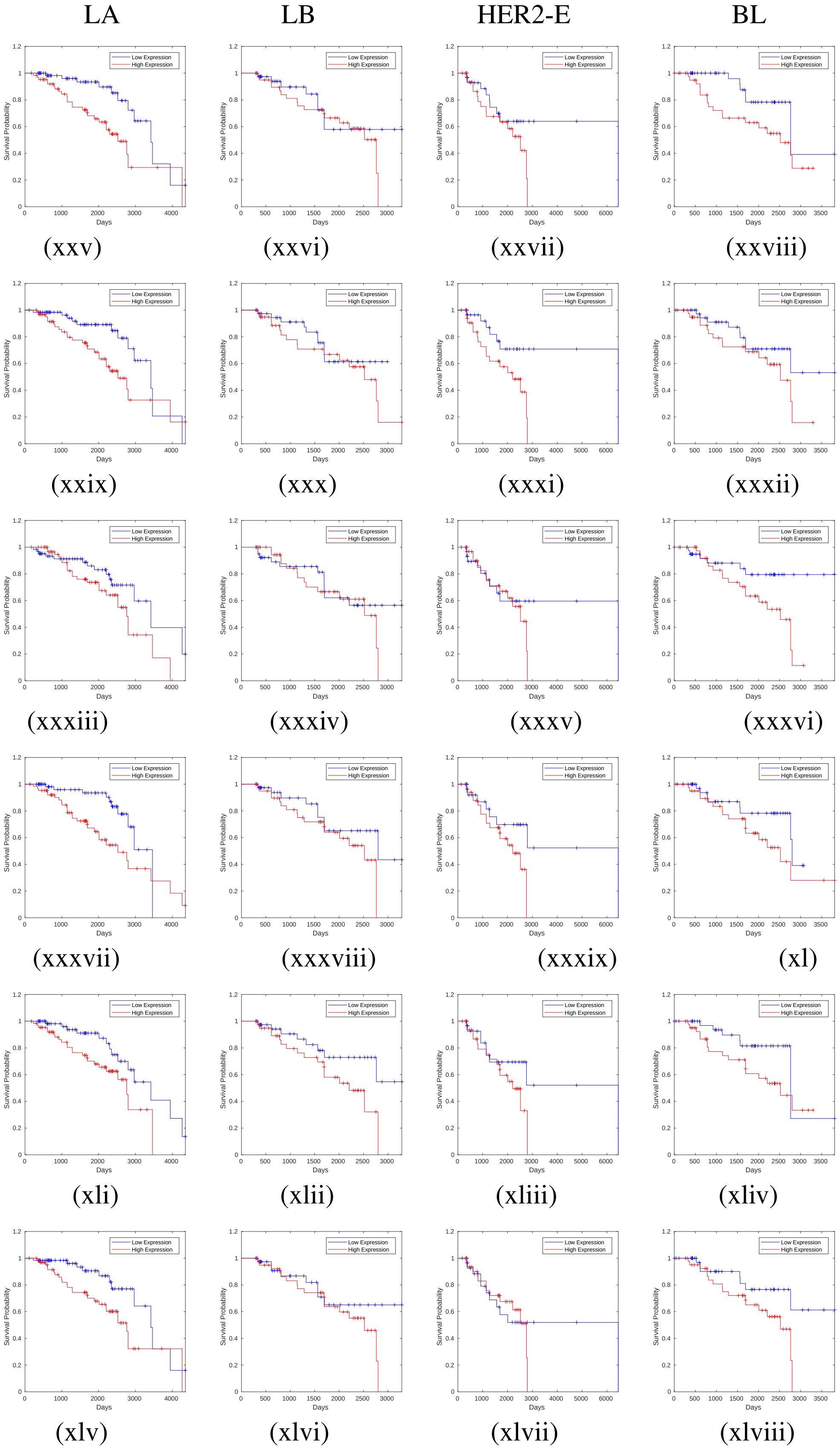
LA
LB
HER2-E
BL
Low Expression
High Expression
Survival Probability
Days
(xxv)
(xxvi)
(xxvii)
(xxviii)
(xxix)
(xxx)
(xxxi)
(xxxii)
(xxxiii)
(xxxiv)
(xxxv)
(xxxvi)
(xxxvii)
(xxxviii)
(xxxix)
(xl)
(xli)
(xlii)
(xliii)
(xliv)
(xlv)
(xlvi)
(xlvii)
(xlviii)

LA LB HER2-E BL

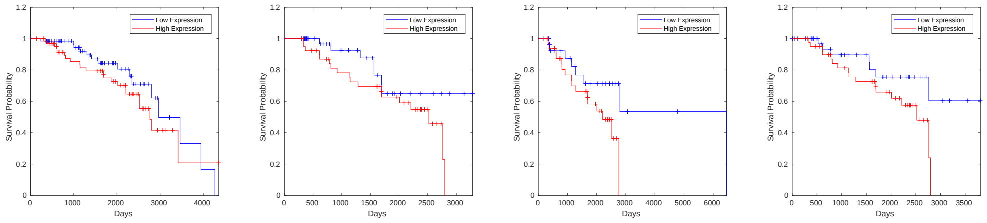

(xlix) (l) (li) (lii)

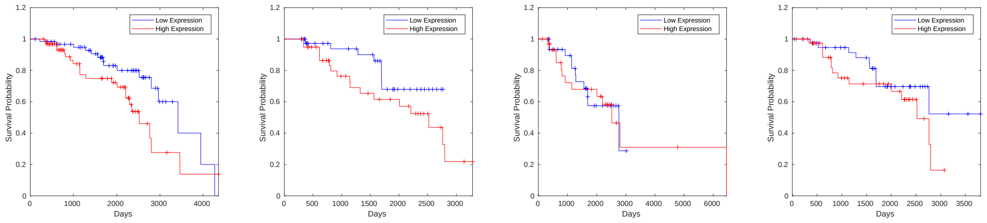

(liii) (liv) (lv) (lvi)

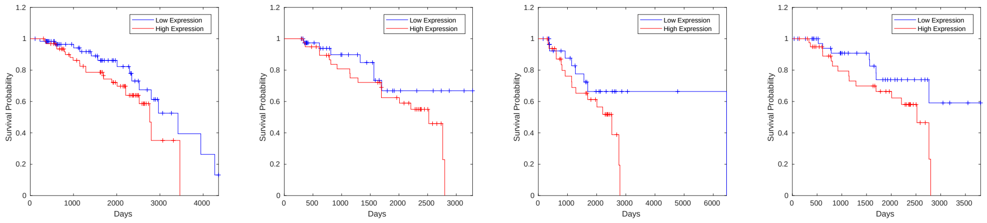

(lvii) (lviii) (lix) (lx)

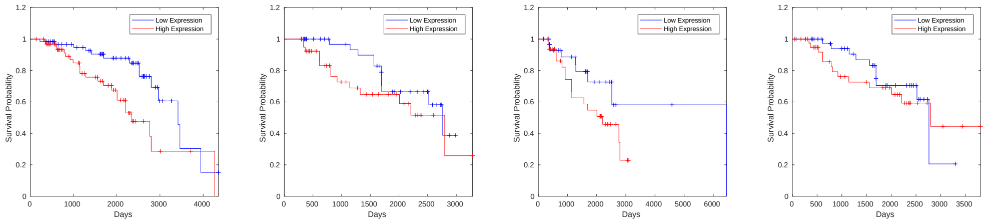

(lxi) (lxii) (lxiii) (lxiv)

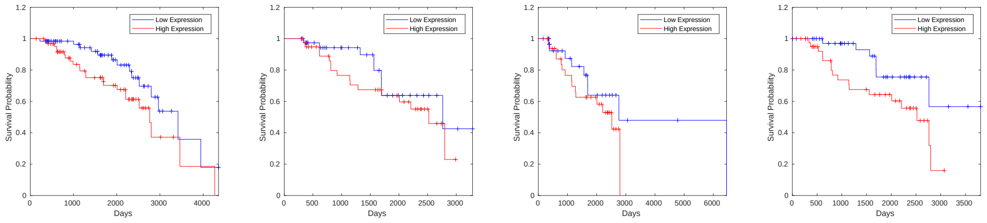

(lxv) (lxvi) (lxvii) (lxviii)

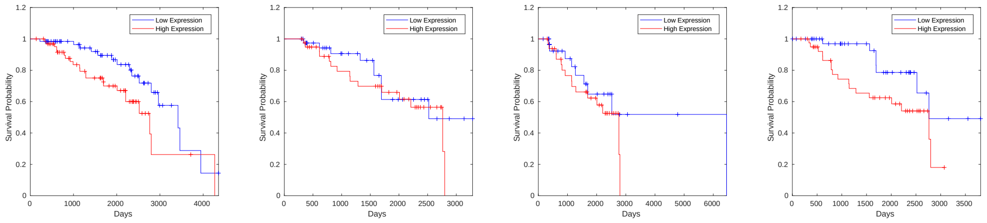

(lxix) (lxx) (lxxi) (lxxii)

LA	LB	HER2-E	BL

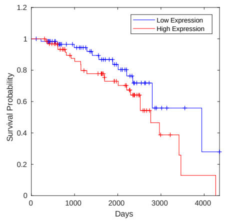

(lxxiii)

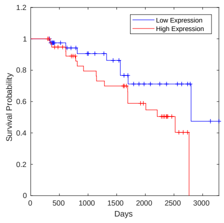

(lxxiv)

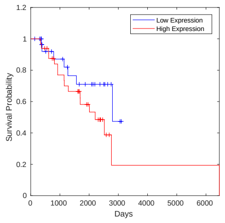

(lxxv)

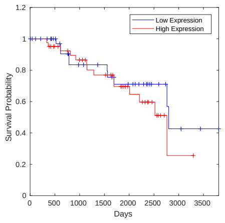

(lxxvi)

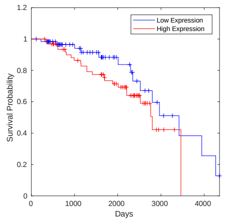

(lxxvii)

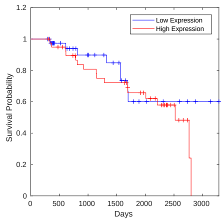

(lxxviii)

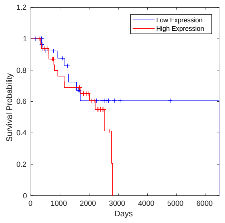

(lxxix)

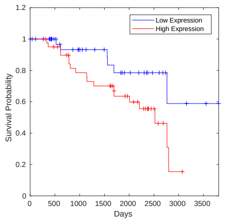

(lxxx)

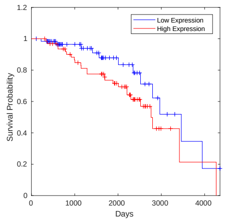

(lxxxi)

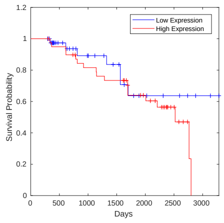

(lxxxii)

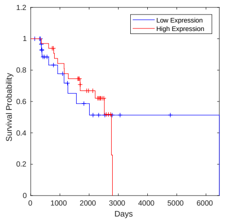

(lxxxiii)

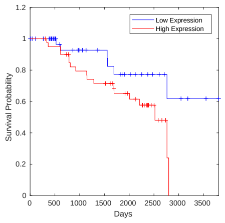

(lxxxiv)

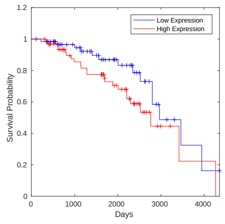

(lxxxv)

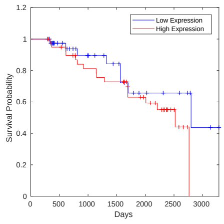

(lxxxvi)

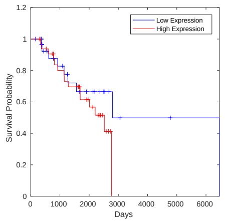

(lxxxvii)

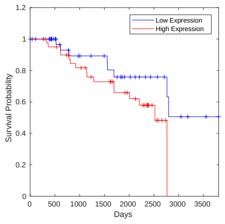

(lxxxviii)

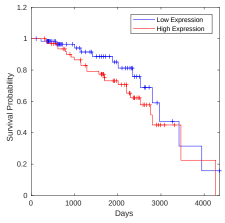

(lxxxix)

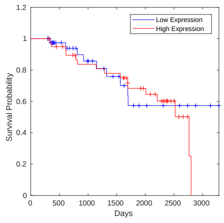

(xc)

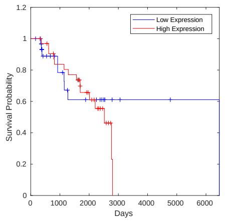

(xci)

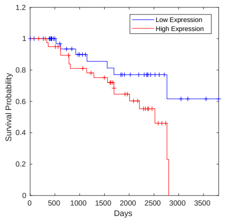

(xcii)

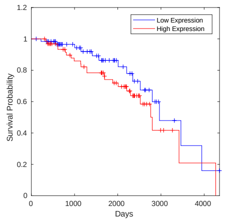

(xciii)

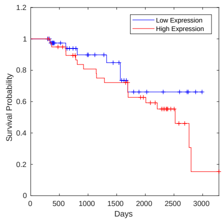

(xciv)

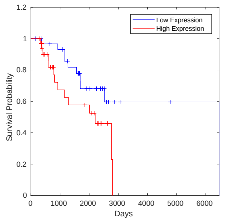

(xcv)

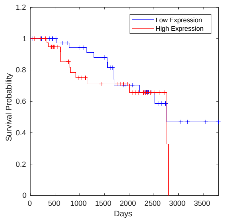

(xcvi)

LA LB HER2-E BL

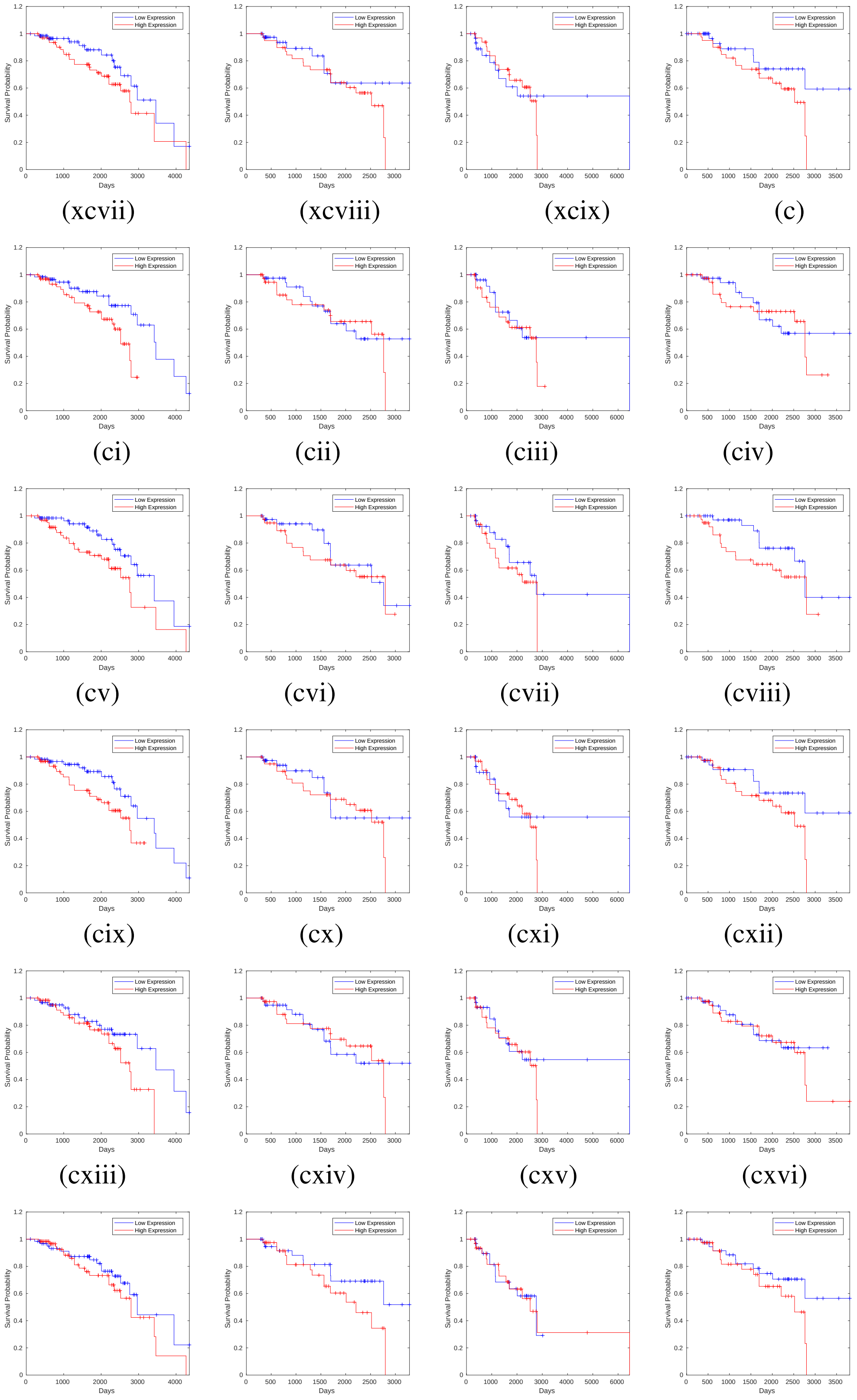

(xcvii) (xcviii) (xcix) (c)

(ci) (cii) (ciii) (civ)

(cv) (cvi) (cvii) (cviii)

(cix) (cx) (cxi) (cxii)

(cxiii) (cxiv) (cxv) (cxvi)

(cxvii) (cxviii) (cxix) (cxx)

LA | LB | HER2-E | BL

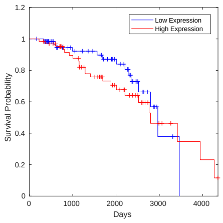

(cxxi)

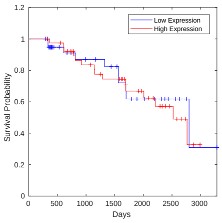

(cxxii)

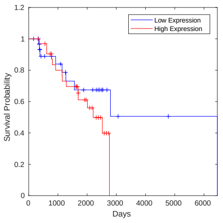

(cxxiii)

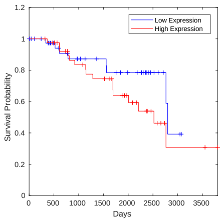

(cxxiv)

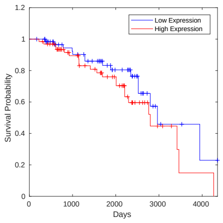

(cxxv)

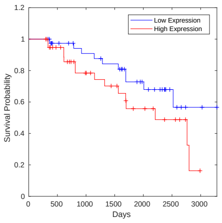

(cxxvi)

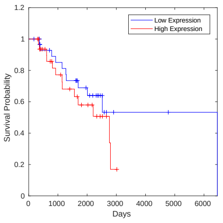

(cxxvii)

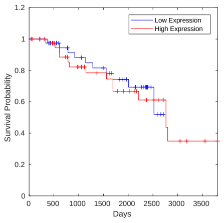

(cxxviii)

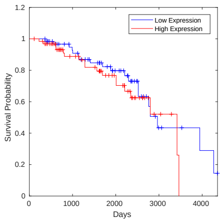

(cxxix)

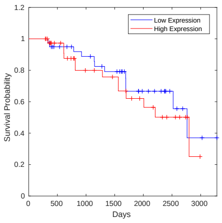

(cxxx)

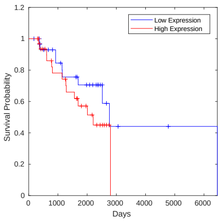

(cxxxi)

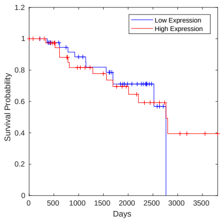

(cxxxii)

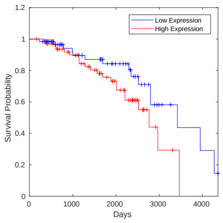

(cxxxiii)

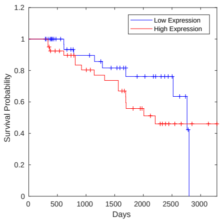

(cxxxiv)

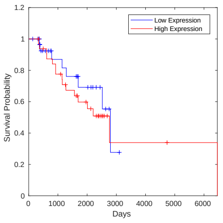

(cxxxv)

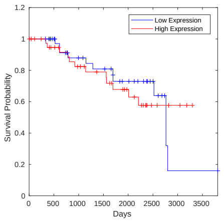

(cxxxvi)

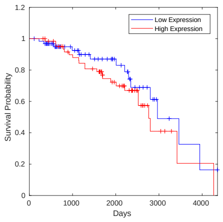

(cxxxvii)

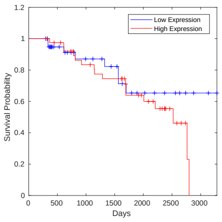

(cxxxviii)

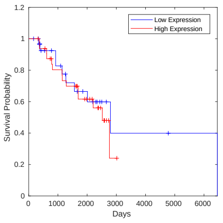

(cxxxix)

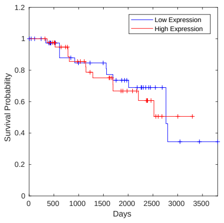

(cxl)

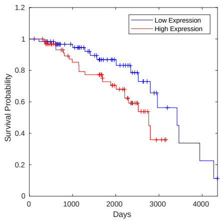

(cxli)

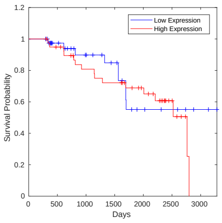

(cxlii)

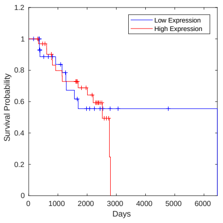

(cxliii)

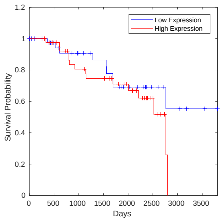

(cxliv)

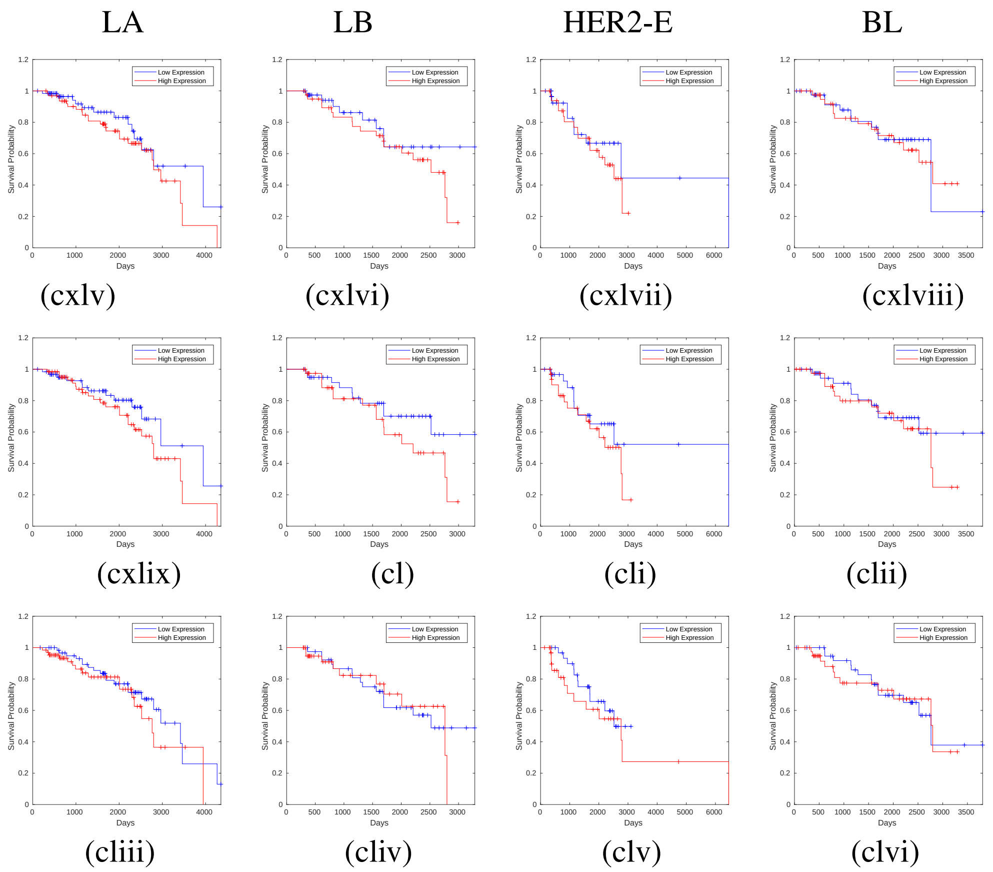

Figure S1: Survival plots for 4-star miRNAs in LA, LB, HER2-E and BL as (i)-(iv):hsa-miR-224-5p, (v)-(viii):hsa-miR-335-3p, (ix)-(xii):hsa-miR-326, (xiii)-(xvi):hsa-miR-10a-5p, (xvii)-(xx):hsa-miR-217, (xxi)-(xxiv):hsa-miR-10b-3p, (xxv)-(xxviii):hsa-miR-378a-5p, (xxix)-(xxxii):hsa-miR-193a-5p, (xxxiii)-(xxxvi):hsa-miR-664a-3p, (xxxvii)-(xl):hsa-miR-30c-2-3p, (xli)-(xliv):hsa-miR-511-5p, (xlv)-(xlviii):hsa-miR-143-3p, (xlix)-(lii):hsa-miR-10b-5p, (liii)-(lvi):hsa-miR-22-3p, (lvii)-(lx):hsa-miR-140-3p, (lxi)-(lxiv):hsa-miR-338-3p, (lxv)-(lxviii):hsa-miR-451a, (lxix)-(lxxii):hsa-miR-486-5p, (lxxiii)-(lxxvi):hsa-miR-28-3p, (lxxvii)-(lxxx):hsa-miR-139-5p, (lxxxi)-(lxxxiv):hsa-miR-125b-2-3p, (lxxxv)-(lxxxviii):hsa-miR-100-5p, (lxxxix)-(xcii):hsa-miR-195-5p, (xciii)-(xcvi):hsa-miR-584-5p, (xcvii)-(c):hsa-let-7c-5p, (ci)-(civ):hsa-miR-574-3p, (cv)-(cviii):hsa-miR-144-5p, (cix)-(cxii):hsa-miR-145-5p, (cxiii)-(cxvi):hsa-let-7e-3p, (cxvii)-(cxx):hsa-miR-24-1-5p, (cxxi)-(cxxiv):hsa-miR-30a-3p, (cxxv)-(cxxviii):hsa-miR-362-5p, (cxxix)-(cxxxii):hsa-miR-339-5p, (cxxxiii)-(cxxxvi):hsa-miR-361-3p, (cxxxvii)-(cxl):hsa-miR-30e-3p, (cxli)-(cxliv):hsa-miR-145-3, (cxlv)-(cxlviii):hsa-miR-29a-3p, (cxlix)-(clii):hsa-miR-34a-5p, (cliii)-(clvi):hsa-miR-193b-5p, where blue line indicates low expression group and red line indicates high expression group

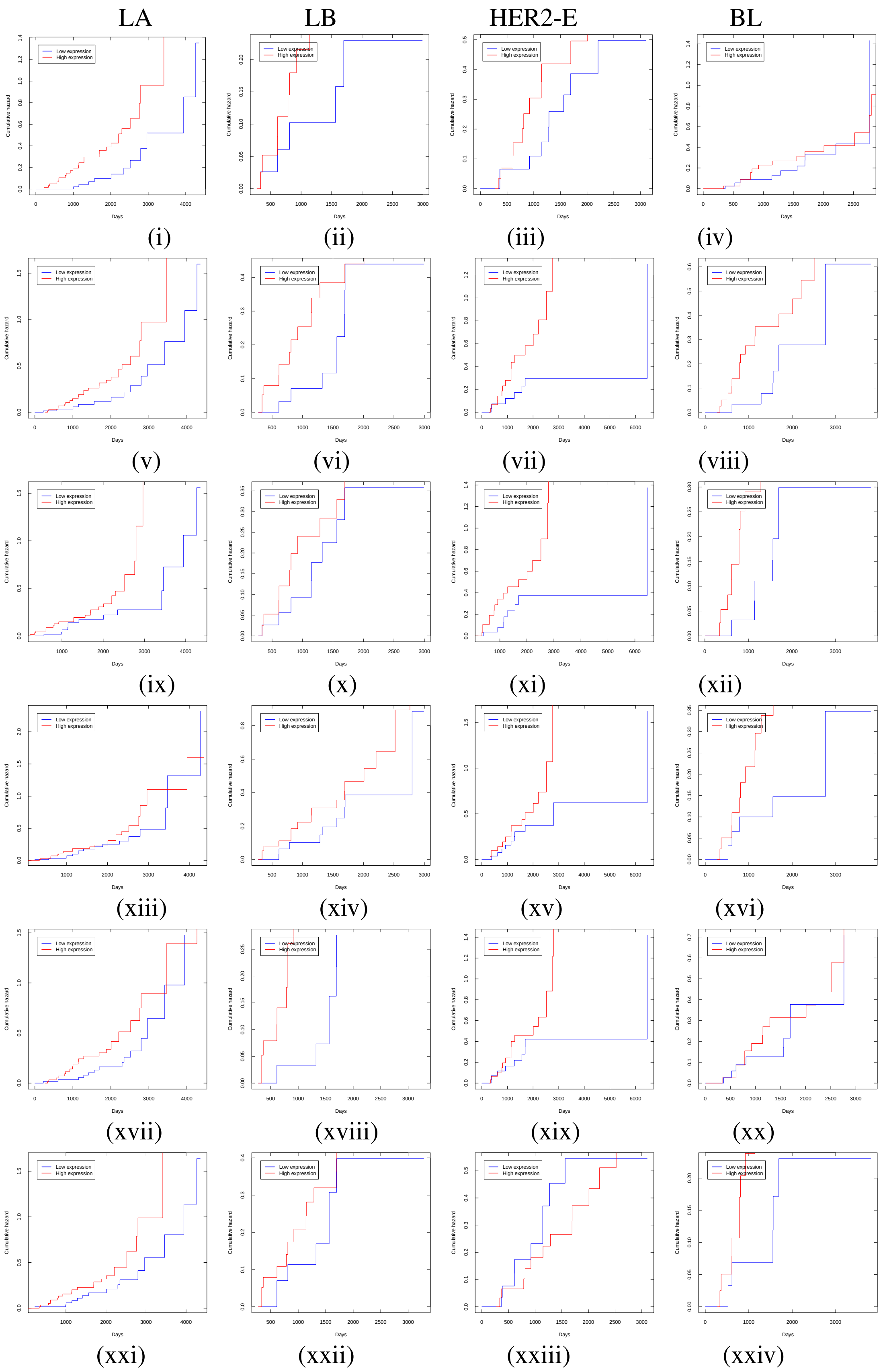
LA
LB
HER2-E
BL
Low expression
High expression
Cumulative hazard
Days
(i)
(ii)
(iii)
(iv)
(v)
(vi)
(vii)
(viii)
(ix)
(x)
(xi)
(xii)
(xiii)
(xiv)
(xv)
(xvi)
(xvii)
(xviii)
(xix)
(xx)
(xxi)
(xxii)
(xxiii)
(xxiv)

LA LB HER2-E BL

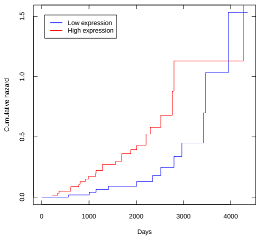

(xxv)

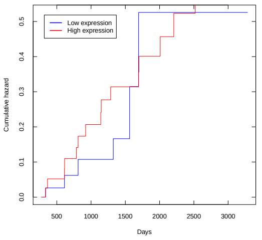

(xxvi)

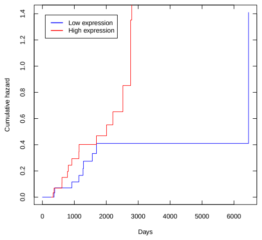

(xxvii)

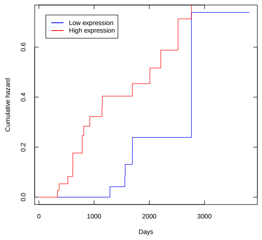

(xxviii)

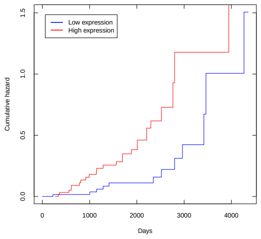

(xxix)

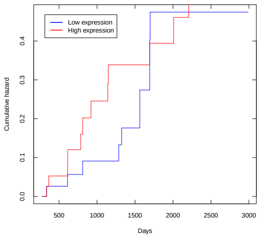

(xxx)

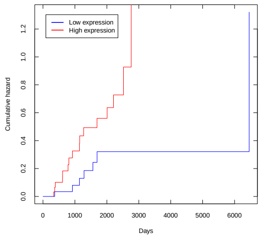

(xxxi)

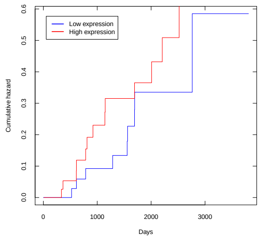

(xxxii)

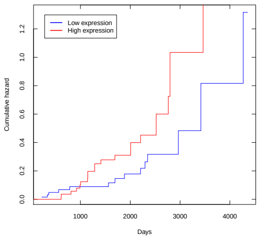

(xxxiii)

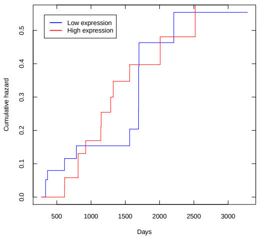

(xxxiv)

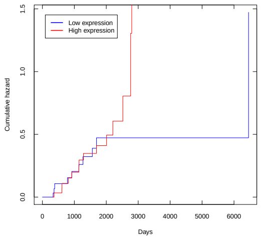

(xxxv)

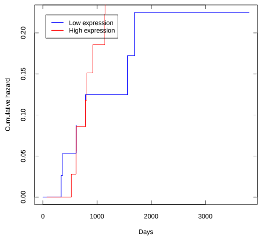

(xxxvi)

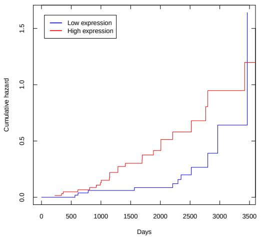

(xxxvii)

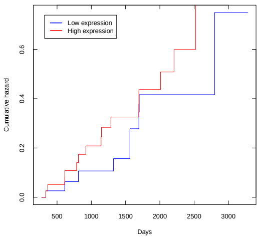

(xxxviii)

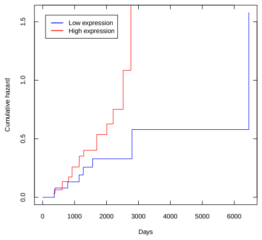

(xxxix)

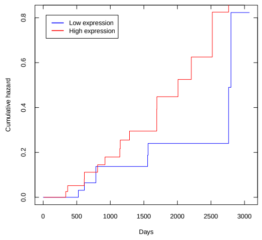

(xl)

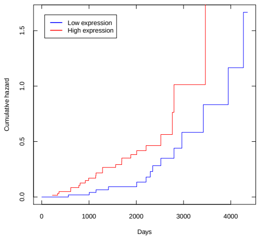

(xli)

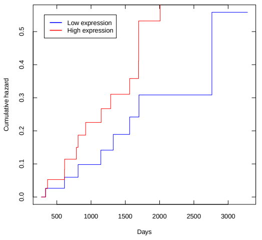

(xlii)

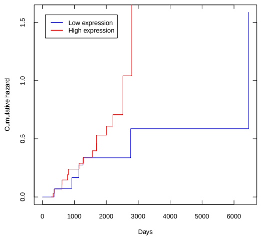

(xliii)

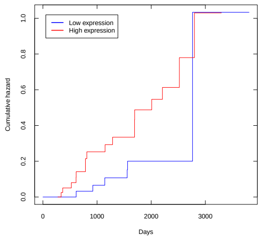

(xliv)

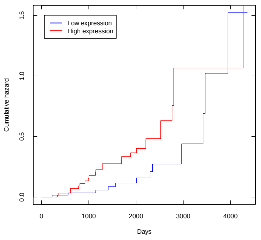

(xlv)

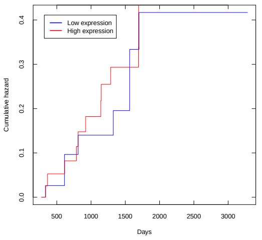

(xlvi)

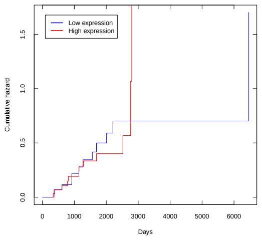

(xlvii)

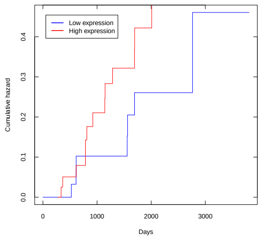

(xlviii)

LA | LB | HER2-E | BL

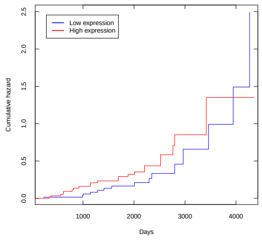

(xlix)

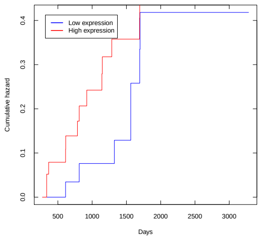

(l)

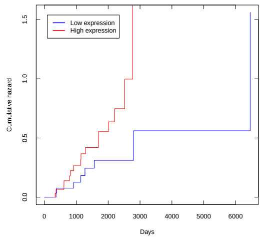

(li)

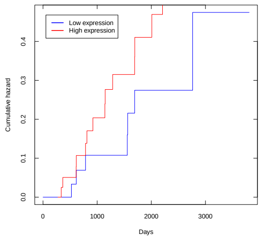

(lii)

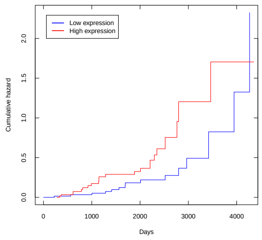

(liii)

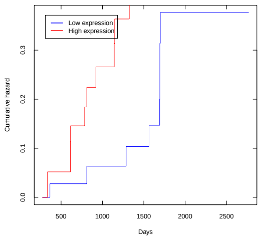

(liv)

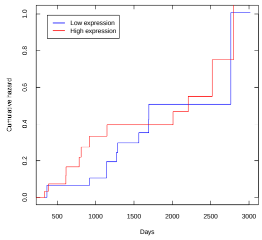

(lv)

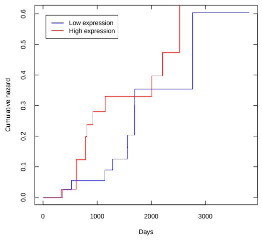

(lvi)

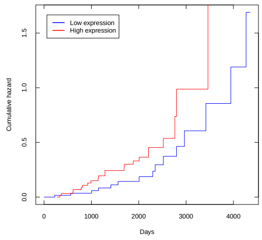

(lvii)

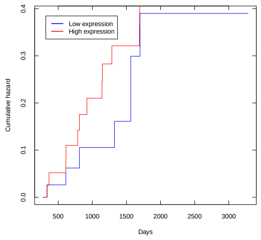

(lviii)

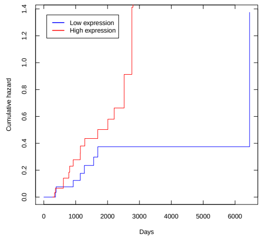

(lix)

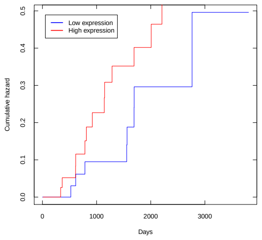

(lx)

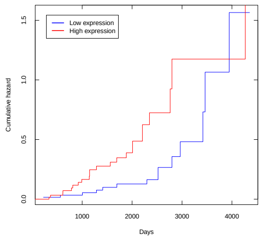

(lxi)

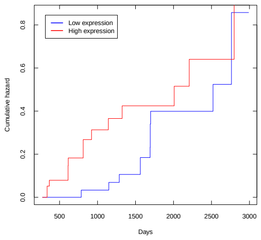

(lxii)

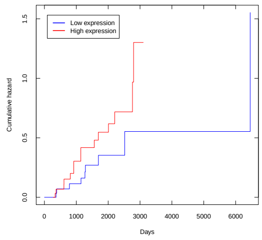

(lxiii)

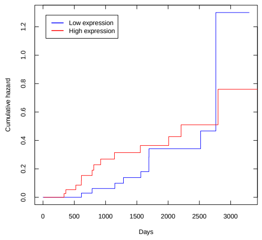

(lxiv)

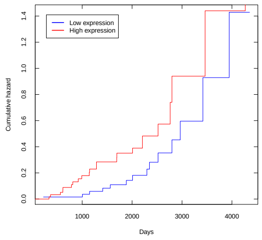

(lxv)

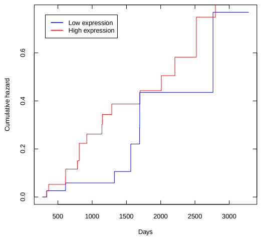

(lxvi)

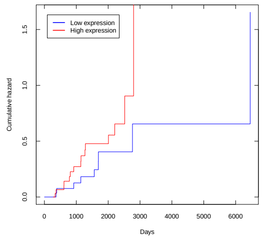

(lxvii)

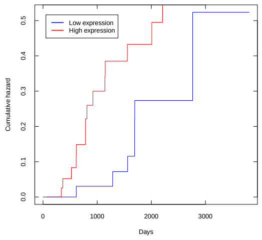

(lxviii)

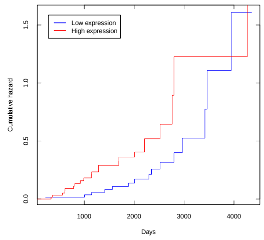

(lxix)

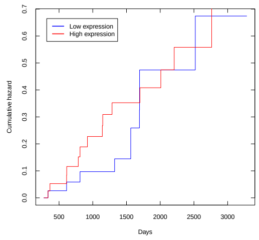

(lxx)

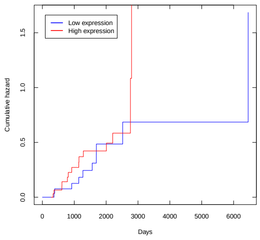

(lxxi)

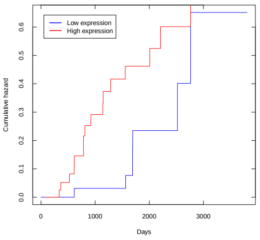

(lxxii)

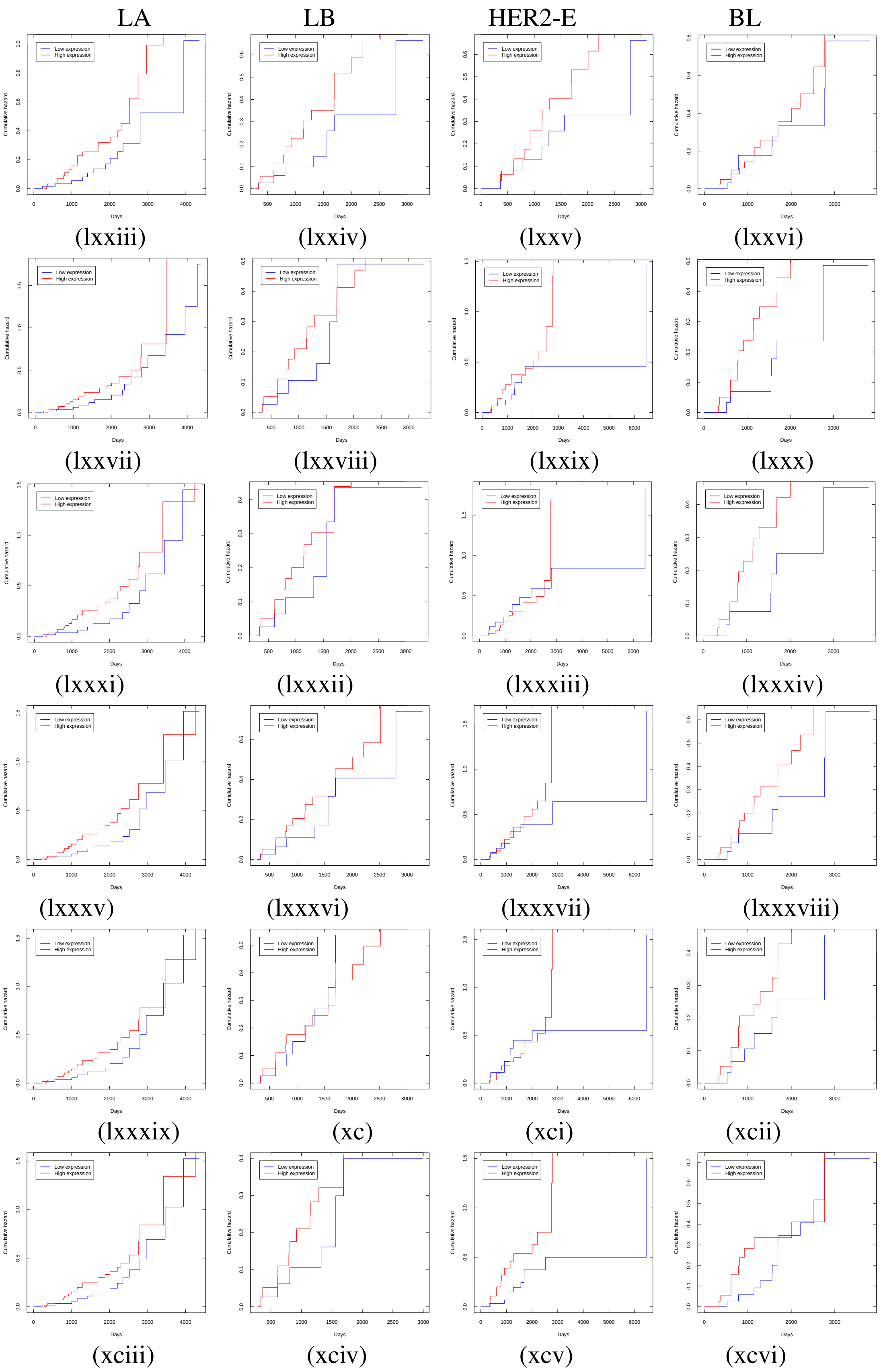
LA
LB
HER2-E
BL
Low expression
High expression
Cumulative hazard
Days
(lxxiii)
(lxxiv)
(lxxv)
(lxxvi)
(lxxvii)
(lxxviii)
(lxxix)
(lxxx)
(lxxxi)
(lxxxii)
(lxxxiii)
(lxxxiv)
(lxxxv)
(lxxxvi)
(lxxxvii)
(lxxxviii)
(lxxxix)
(xc)
(xci)
(xcii)
(xciii)
(xciv)
(xcv)
(xcvi)

LA	LB	HER2-E	BL

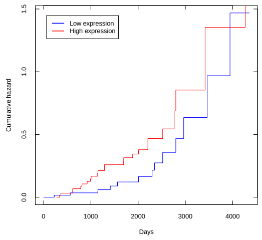

(xcvii)

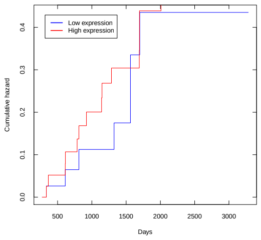

(xcviii)

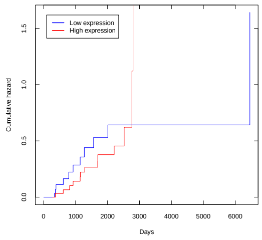

(xcix)

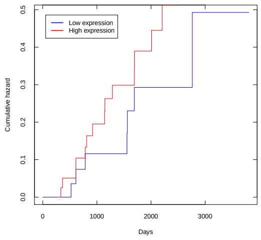

(c)

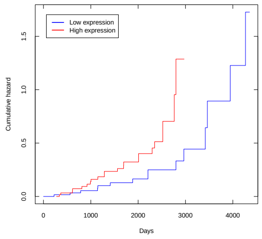

(ci)

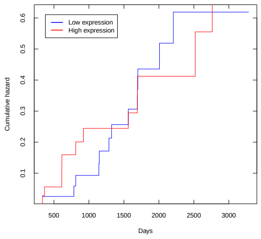

(cii)

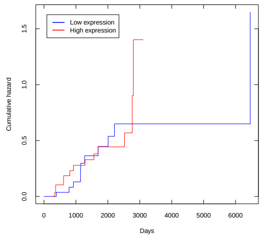

(ciii)

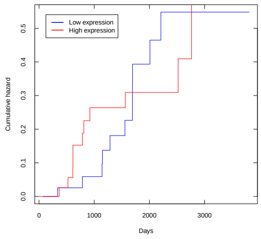

(civ)

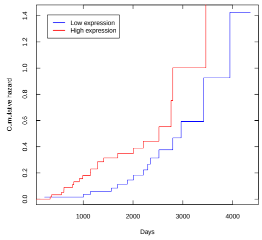

(cv)

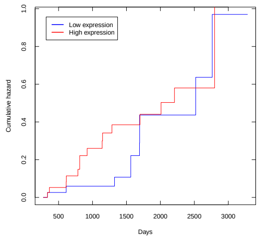

(cvi)

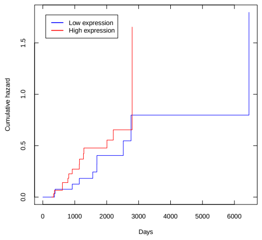

(cvii)

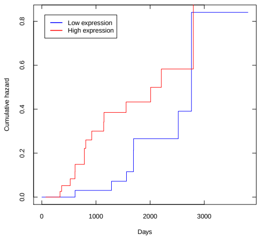

(cviii)

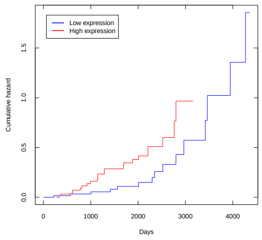

(cix)

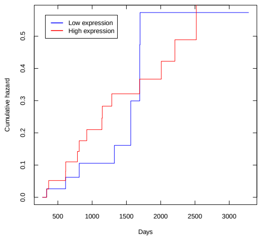

(cx)

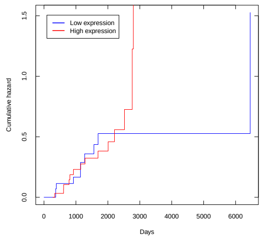

(cxi)

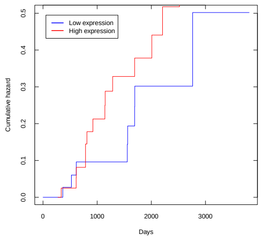

(cxii)

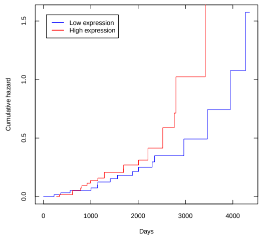

(cxiii)

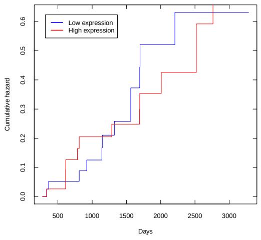

(cxiv)

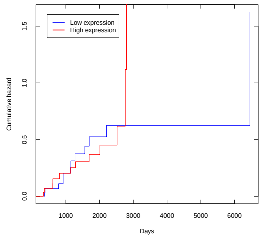

(cxv)

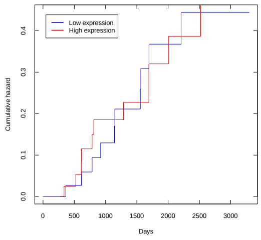

(cxvi)

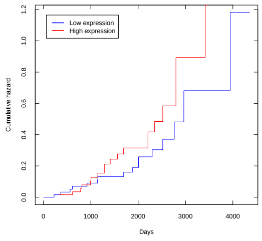

(cxvii)

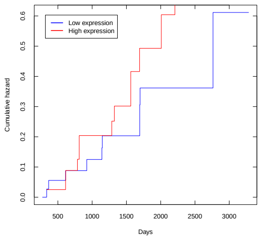

(cxviii)

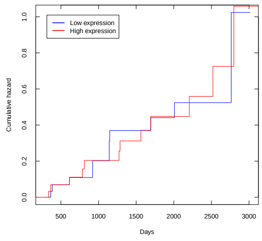

(cxix)

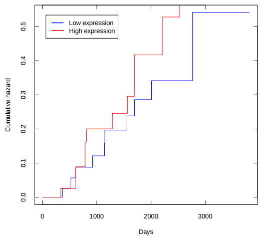

(cxx)

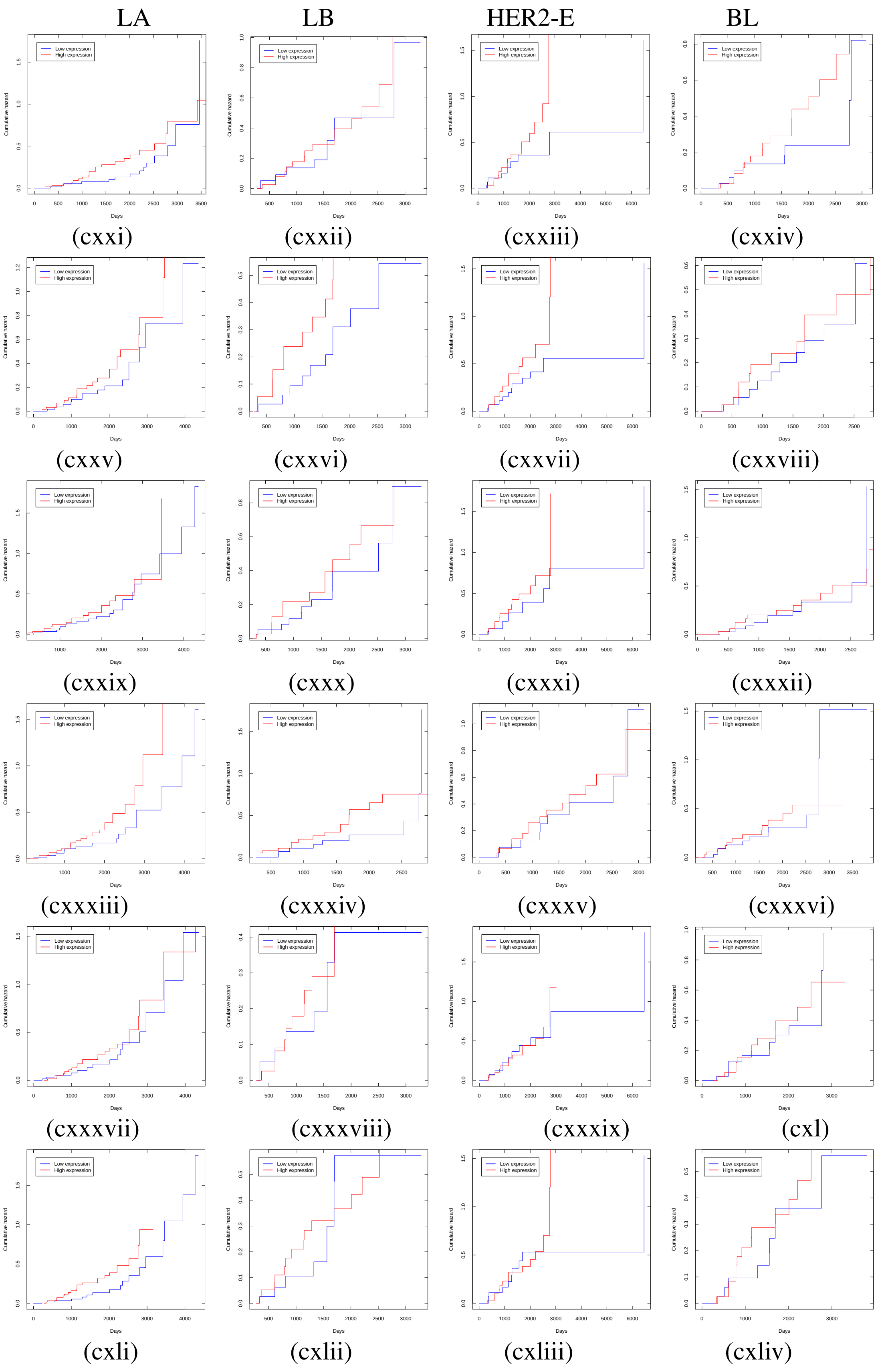

(cxxi) (cxxii) (cxxiii) (cxxiv)

(cxxv) (cxxvi) (cxxvii) (cxxviii)

(cxxix) (cxxx) (cxxxi) (cxxxii)

(cxxxiii) (cxxxiv) (cxxxv) (cxxxvi)

(cxxxvii) (cxxxviii) (cxxxix) (cxl)

(cxli) (cxlii) (cxliii) (cxliv)

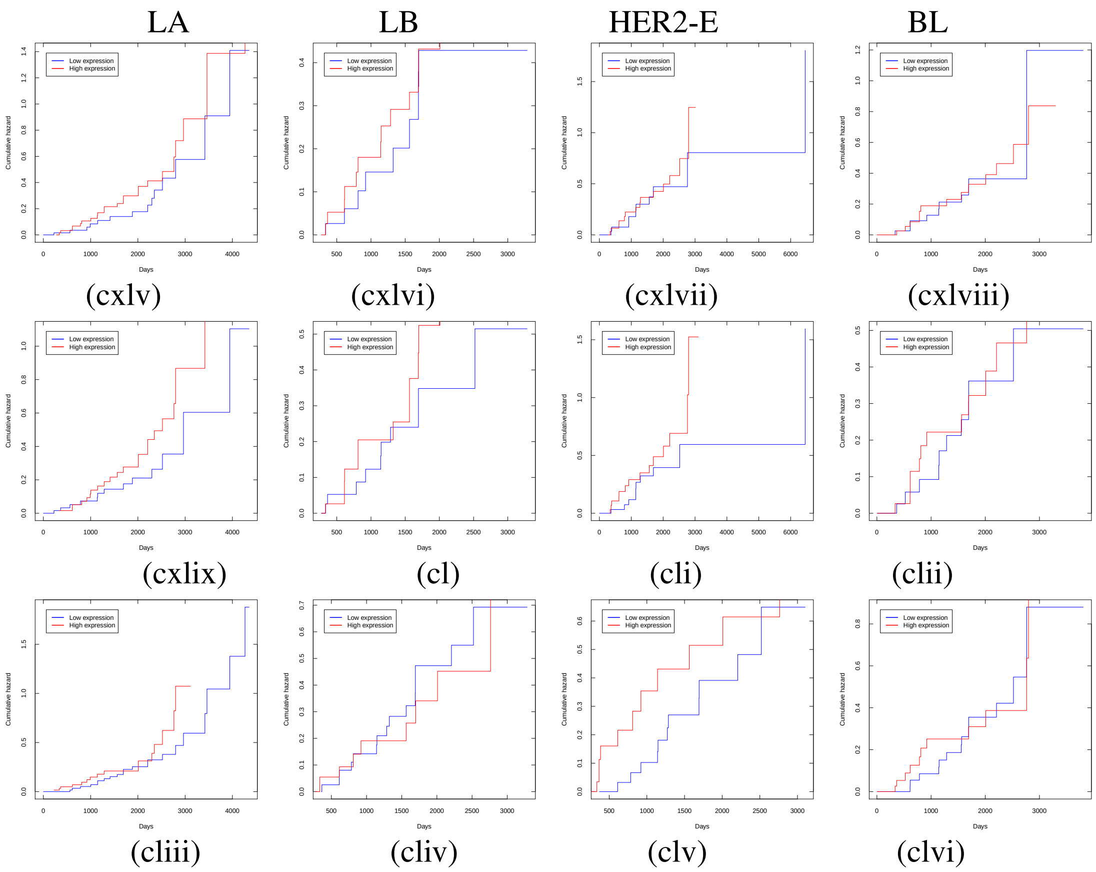

Figure S2: Cumulative hazard plots, using Nelson-Aalen estimator for 4-star miRNAs in LA, LB, HER2-E and BL as (i)-(iv):hsa-miR-224-5p, (v)-(viii):hsa-miR-335-3p, (ix)-(xii):hsa-miR-326, (xiii)-(xvi):hsa-miR-10a-5p, (xvii)-(xx):hsa-miR-217, (xxi)-(xxiv):hsa-miR-10b-3p, (xxv)-(xxviii):hsa-miR-378a-5p, (xxix)-(xxxii):hsa-miR-193a-5p, (xxxiii)-(xxxvi):hsa-miR-664a-3p, (xxxvii)-(xl):hsa-miR-30c-2-3p, (xli)-(xliv):hsa-miR-511-5p, (xlv)-(xlviii):hsa-miR-143-3p, (xlix)-(lii):hsa-miR-10b-5p, (liii)-(lvi):hsa-miR-22-3p, (lvii)-(lx):hsa-miR-140-3p, (lxi)-(lxiv):hsa-miR-338-3p, (lxv)-(lxviii):hsa-miR-451a, (lxix)-(lxxii):hsa-miR-486-5p, (lxxiii)-(lxxvi):hsa-miR-28-3p, (lxxvii)-(lxxx):hsa-miR-139-5p, (lxxxi)-(lxxxiv):hsa-miR-125b-2-3p, (lxxxv)-(lxxxviii):hsa-miR-100-5p, (lxxxix)-(xcii):hsa-miR-195-5p, (xciii)-(xcvi):hsa-miR-584-5p, (xcvii)-(c):hsa-let-7c-5p, (ci)-(civ):hsa-miR-574-3p, (cv)-(cviii):hsa-miR-144-5p, (cix)-(cxii):hsa-miR-145-5p, (cxiii)-(cxvi):hsa-let-7e-3p, (cxvii)-(cxx):hsa-miR-24-1-5p, (cxxi)-(cxxiv):hsa-miR-30a-3p, (cxxv)-(cxxviii):hsa-miR-362-5p, (cxxix)-(cxxxii):hsa-miR-339-5p, (cxxxiii)-(cxxxvi):hsa-miR-361-3p, (cxxxvii)-(cxl):hsa-miR-30e-3p, (cxli)-(cxliv):hsa-miR-145-3, (cxlv)-(cxlviii):hsa-miR-29a-3p, (cxlix)-(clii):hsa-miR-34a-5p, (cliii)-(clvi):hsa-miR-193b-5p, where blue line indicates low expression group and red line indicates high expression group

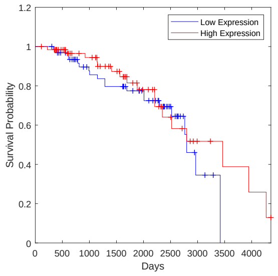

(a) hsa-miR-222-3p

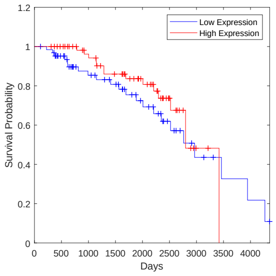

(b) hsa-miR-1180-3p

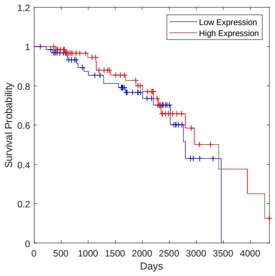

(c) hsa-miR-186-5p

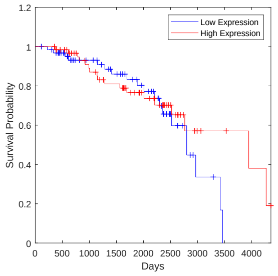

(d) hsa-miR-107

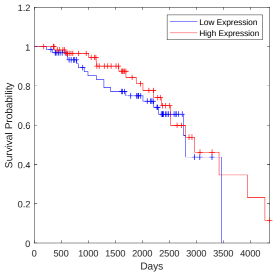

(e) hsa-miR-505-3p

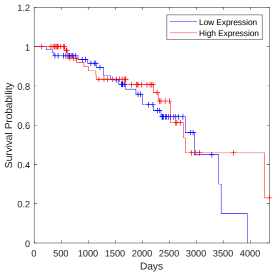

(f) hsa-miR-455-5p

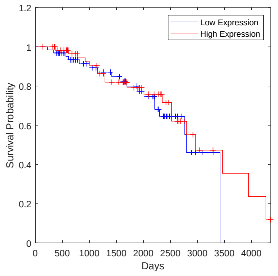

(g) hsa-miR-532-5p

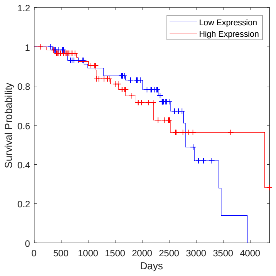

(h) hsa-miR-502-3p

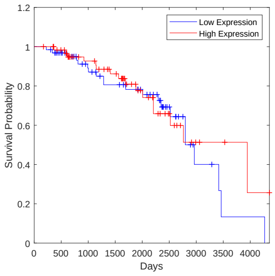

(i) hsa-miR-24-3p

Figure S3: Survival plots for 1-star LA miRNAs where blue line indicates low expression group and red line indicates high expression group

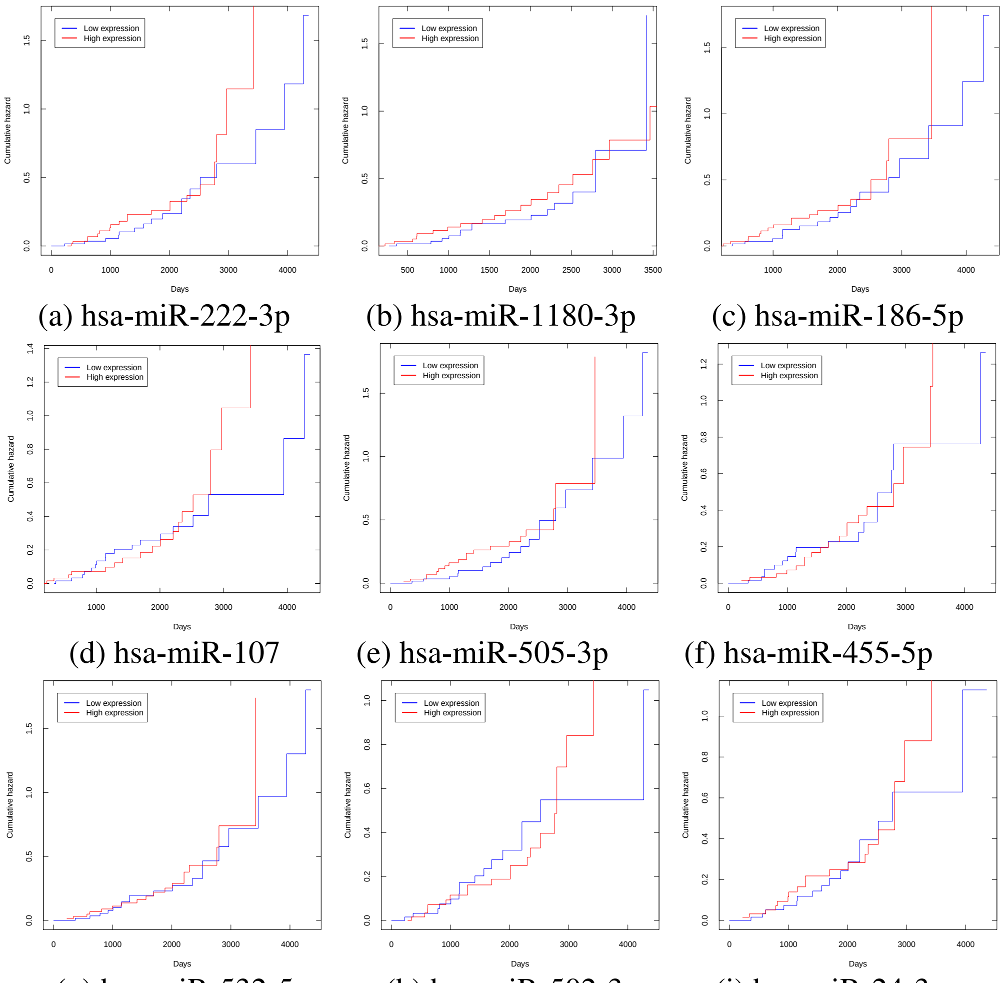

(a) hsa-miR-222-3p (b) hsa-miR-1180-3p (c) hsa-miR-186-5p

(d) hsa-miR-107 (e) hsa-miR-505-3p (f) hsa-miR-455-5p

(g) hsa-miR-532-5p (h) hsa-miR-502-3p (i) hsa-miR-24-3p

Figure S4: Cumulative hazard plots, using Nelson-Aalen estimator for 1-star LA miRNAs where blue line indicates low expression group and red line indicates high expression group

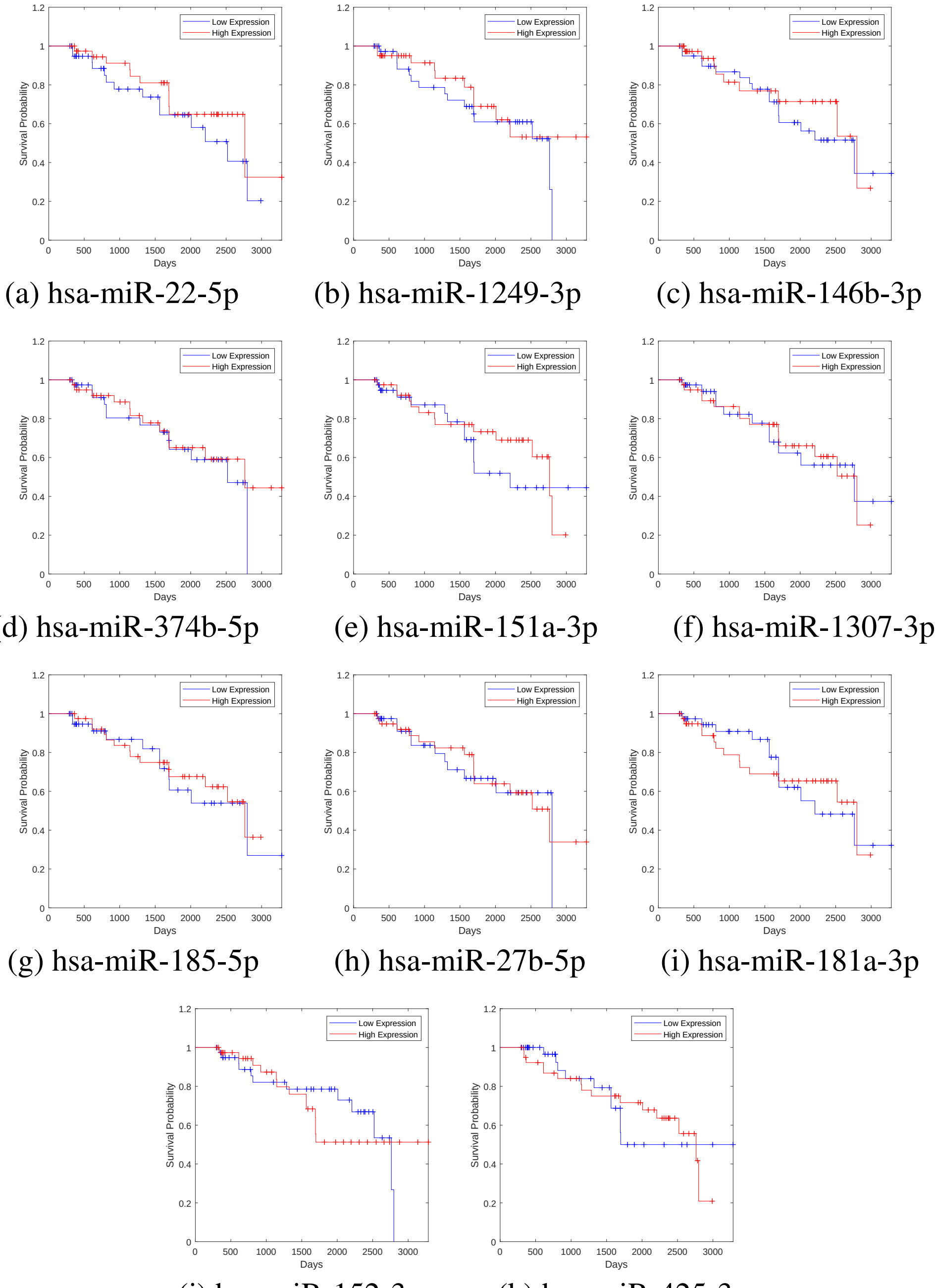

(a) hsa-miR-22-5p (b) hsa-miR-1249-3p (c) hsa-miR-146b-3p

(d) hsa-miR-374b-5p (e) hsa-miR-151a-3p (f) hsa-miR-1307-3p

(g) hsa-miR-185-5p (h) hsa-miR-27b-5p (i) hsa-miR-181a-3p

(j) hsa-miR-152-3p (k) hsa-miR-425-3p

Figure S5: Survival plots for 1-star LB miRNAs where blue line indicates low expression group and red line indicates high expression group

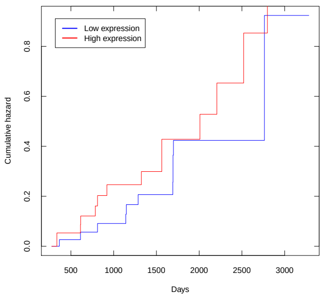

(a) hsa-miR-22-5p

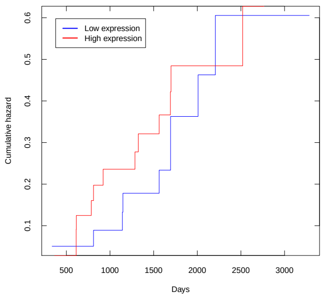

(b) hsa-miR-1249-3p

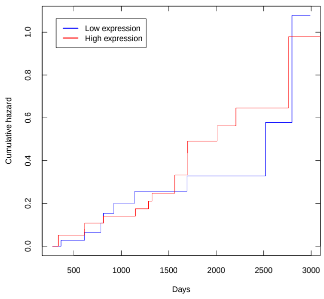

(c) hsa-miR-146b-3p

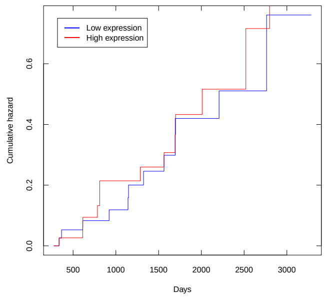

(d) hsa-miR-374b-5p

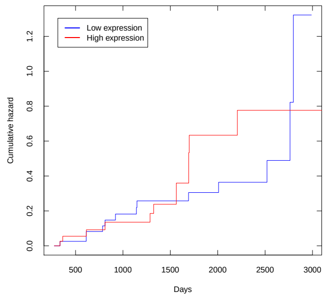

(e) hsa-miR-151a-3p

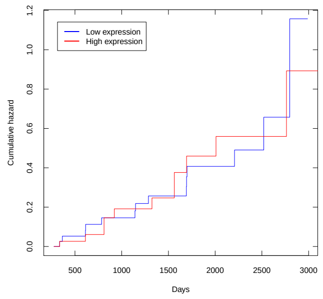

(f) hsa-miR-1307-3p

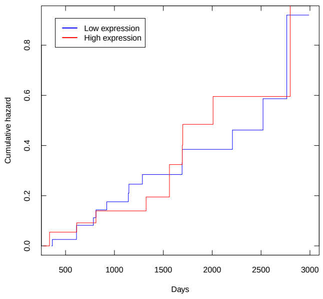

(g) hsa-miR-185-5p

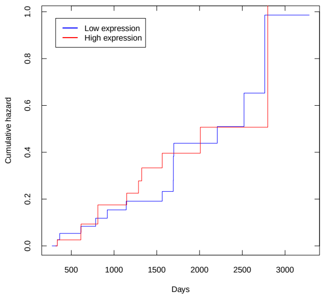

(h) hsa-miR-27b-5p

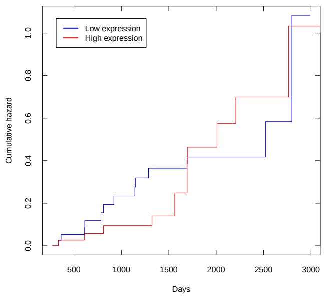

(i) hsa-miR-181a-3p

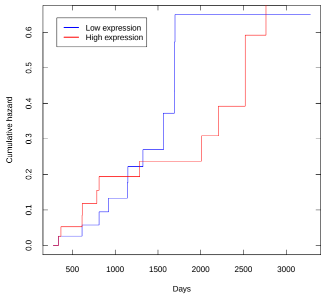

(j) hsa-miR-152-3p

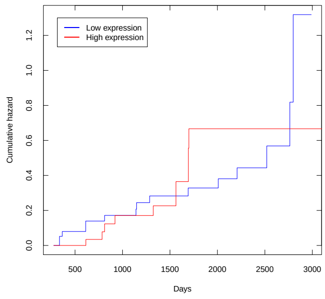

(k) hsa-miR-425-3p

Figure S6: Cumulative hazard plots, using Nelson-Aalen estimator for 1-star LB miRNAs where blue line indicates low expression group and red line indicates high expression group

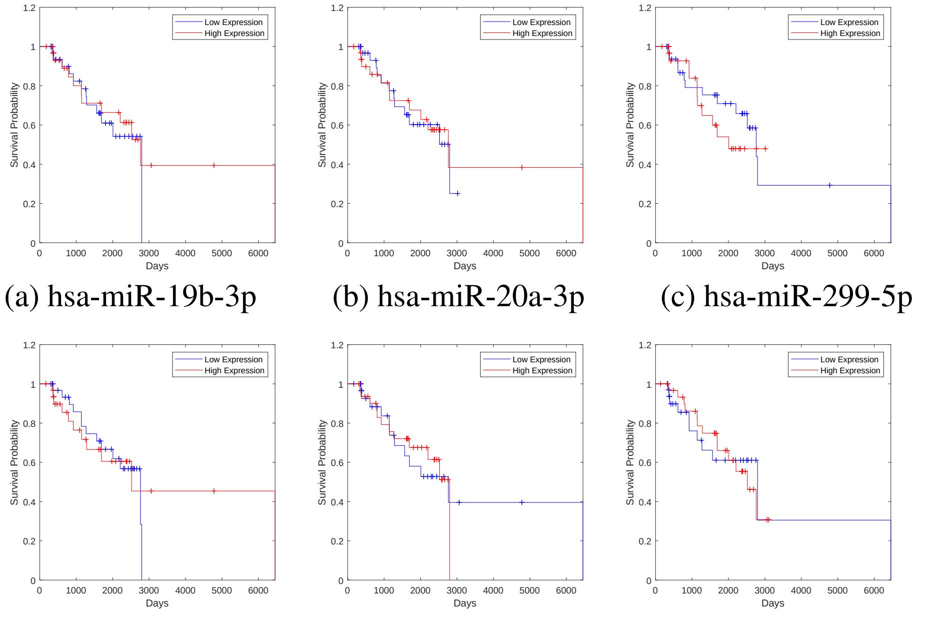

(a) hsa-miR-19b-3p (b) hsa-miR-20a-3p (c) hsa-miR-299-5p

(d) hsa-miR-874-3p (e) hsa-miR-3127-5p (f) hsa-miR-20b-5p

Figure S7: Survival plots for 1-star HER2-E miRNAs where blue line indicates low expression group and red line indicates high expression group

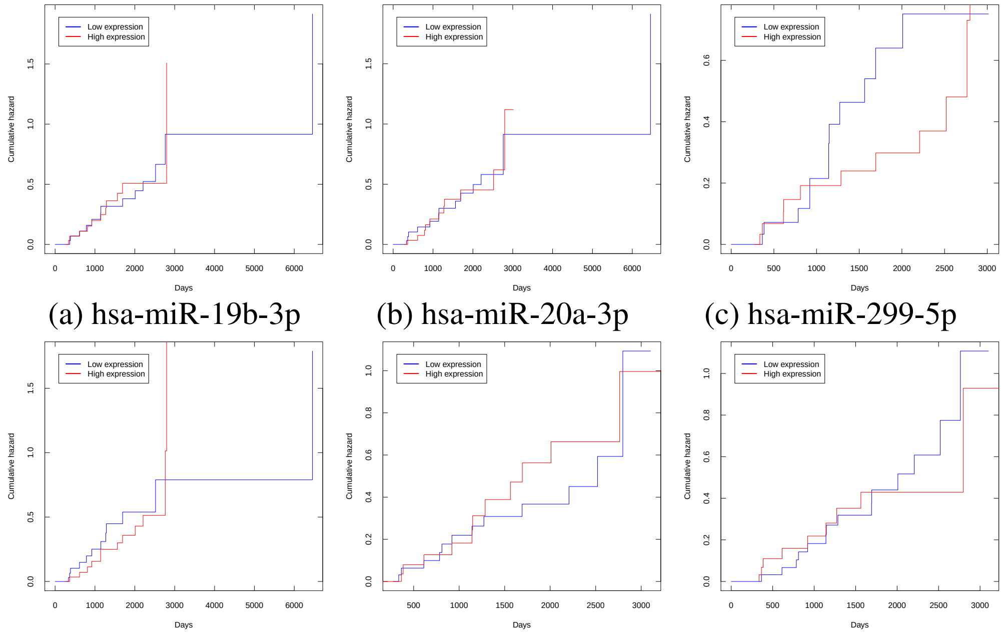

(a) hsa-miR-19b-3p (b) hsa-miR-20a-3p (c) hsa-miR-299-5p

(d) hsa-miR-874-3p (e) hsa-miR-3127-5p (f) hsa-miR-20b-5p

Figure S8: Cumulative hazard plots, using Nelson-Aalen estimator for 1-star HER2-E miRNAs where blue line indicates low expression group and red line indicates high expression group

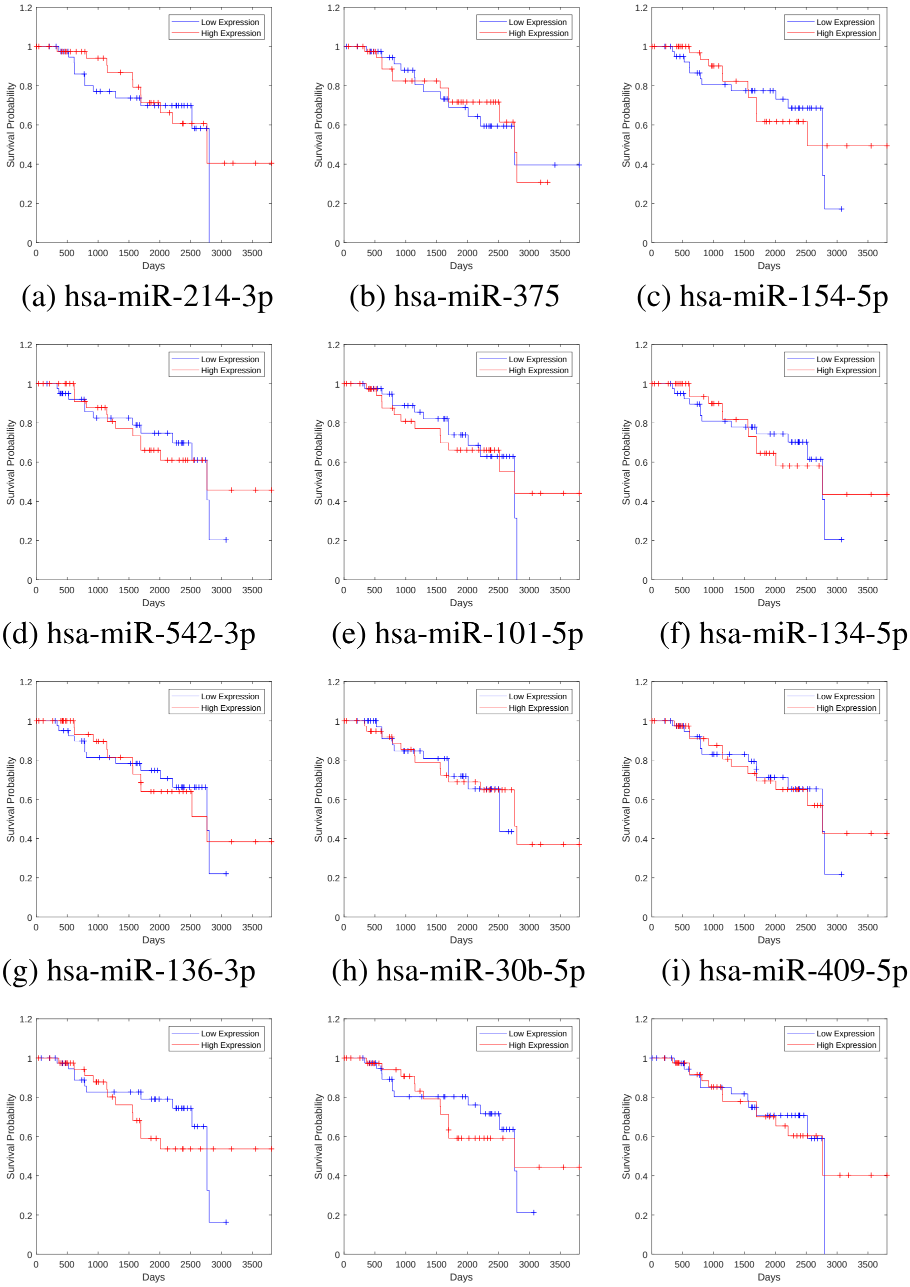

(a) hsa-miR-214-3p (b) hsa-miR-375 (c) hsa-miR-154-5p

(d) hsa-miR-542-3p (e) hsa-miR-101-5p (f) hsa-miR-134-5p

(g) hsa-miR-136-3p (h) hsa-miR-30b-5p (i) hsa-miR-409-5p

(j) hsa-miR-409-3p (k) hsa-miR-539-5p (l) hsa-let-7f-5p

Figure S9: Survival plots for 1-star BL miRNAs where blue line indicates low expression group and red line indicates high expression group

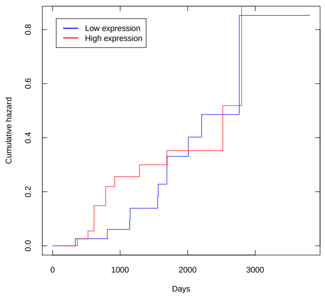

(a) hsa-miR-214-3p

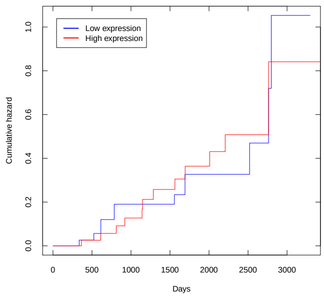

(b) hsa-miR-375

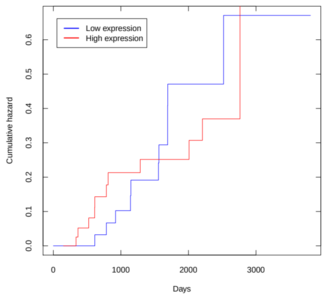

(c) hsa-miR-154-5p

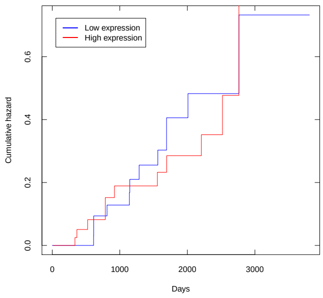

(d) hsa-miR-542-3p

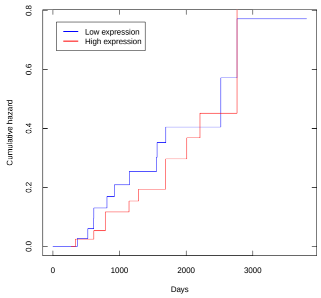

(e) hsa-miR-101-5p

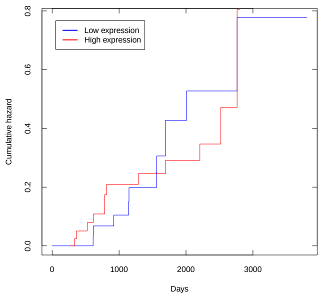

(f) hsa-miR-134-5p

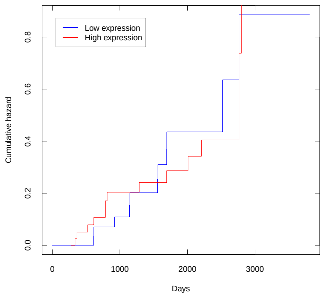

(g) hsa-miR-136-3p

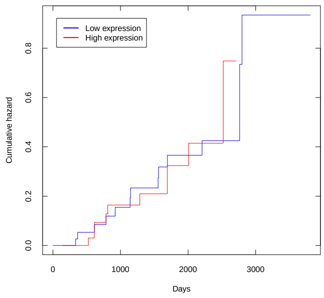

(h) hsa-miR-30b-5p

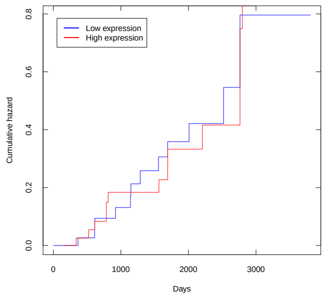

(i) hsa-miR-409-5p

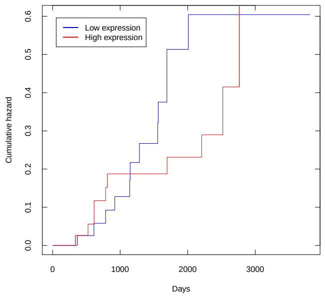

(j) hsa-miR-409-3p

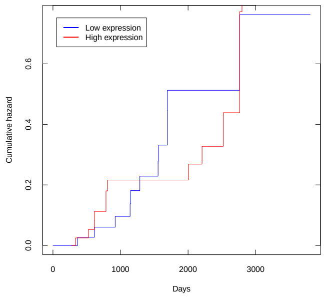

(k) hsa-miR-539-5p

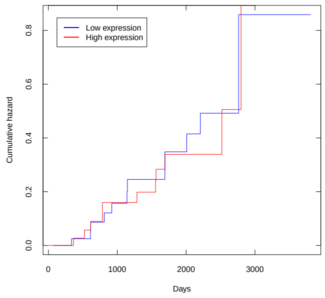

(l) hsa-let-7f-5p

Figure S10: Cumulative hazard plots, using Nelson-Aalen estimator for 1-star BL miRNAs where blue line indicates low expression group and red line indicates high expression group

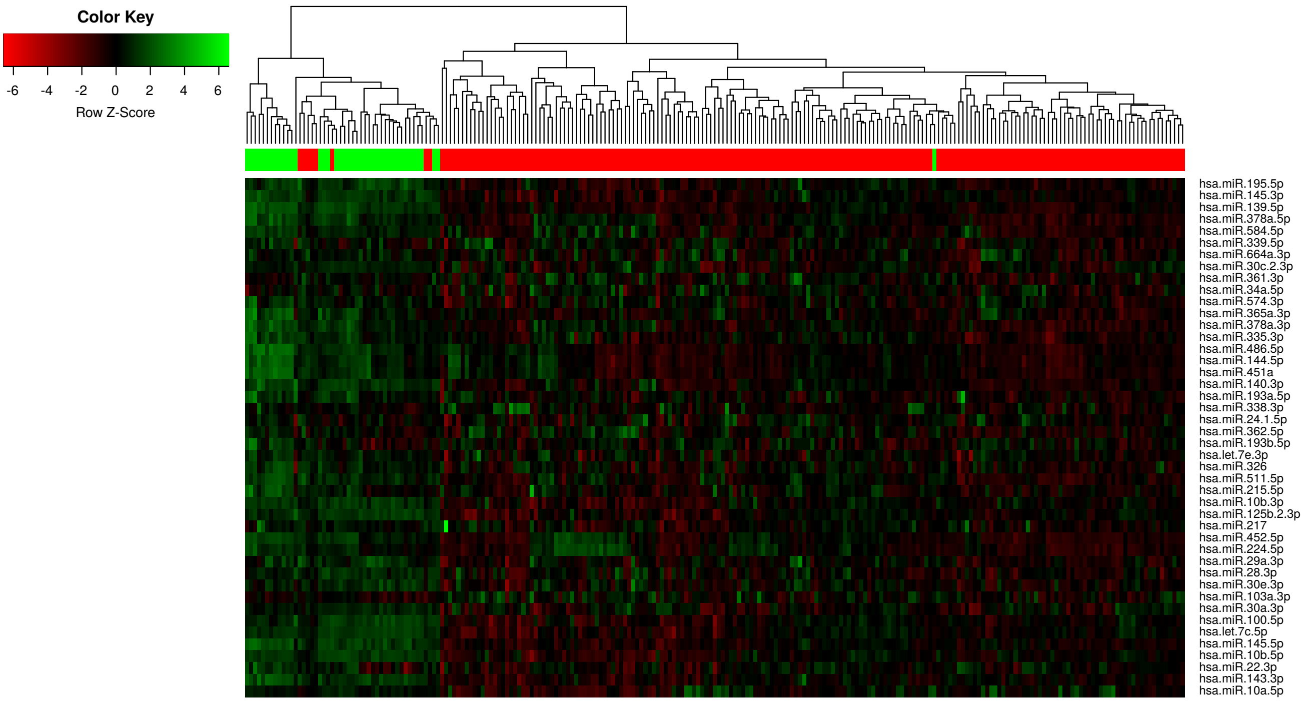

Figure S11: Clustering analysis of the 4-star miRNAs of all patients with control. Color of the top bar represents group: red for cancer (any subtype), green for control

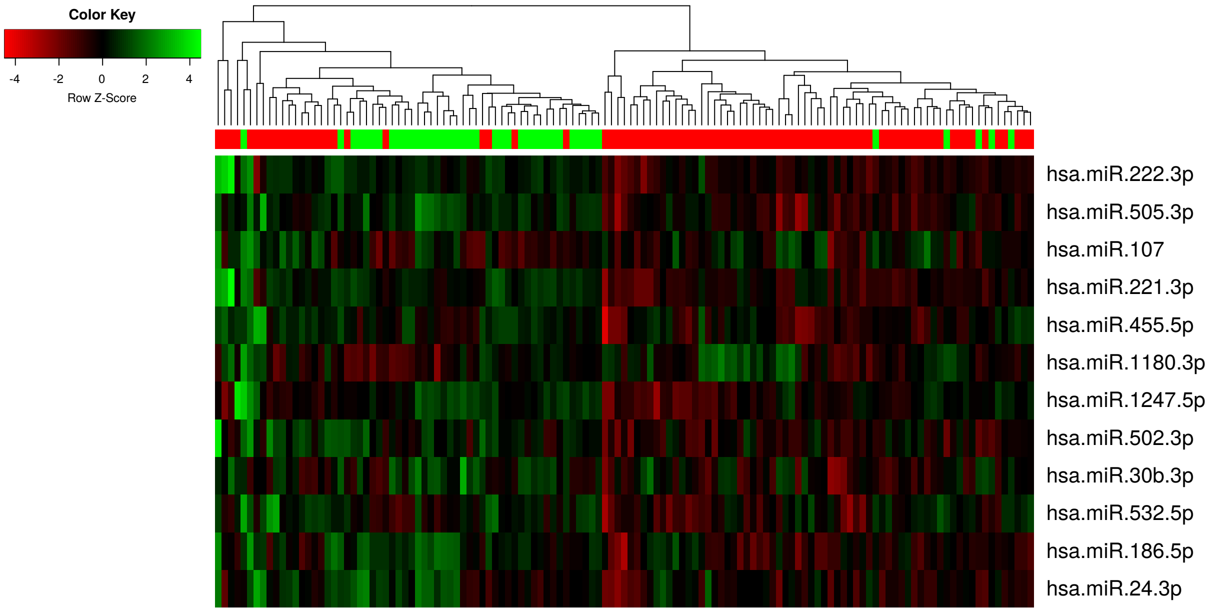
Color Key
-4
-2
0
2
4
Row Z-Score
hsa.miR.222.3p
hsa.miR.505.3p
hsa.miR.107
hsa.miR.221.3p
hsa.miR.455.5p
hsa.miR.1180.3p
hsa.miR.1247.5p
hsa.miR.502.3p
hsa.miR.30b.3p
hsa.miR.532.5p
hsa.miR.186.5p
hsa.miR.24.3p

(a)

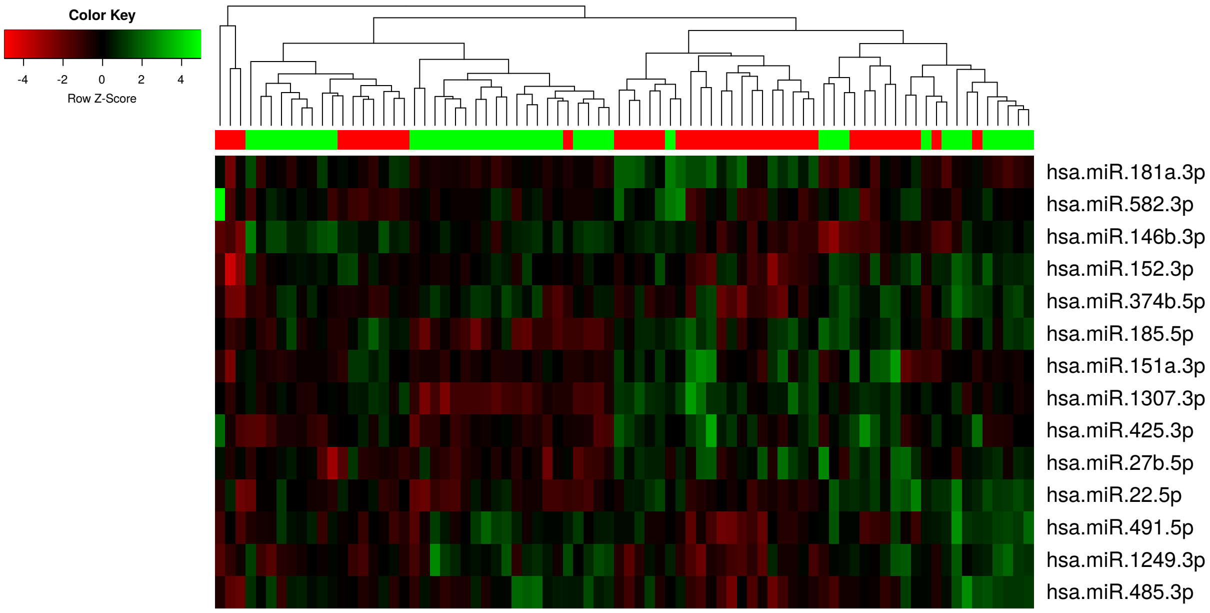
Color Key
-4
-2
0
2
4
Row Z-Score
hsa.miR.181a.3p
hsa.miR.582.3p
hsa.miR.146b.3p
hsa.miR.152.3p
hsa.miR.374b.5p
hsa.miR.185.5p
hsa.miR.151a.3p
hsa.miR.1307.3p
hsa.miR.425.3p
hsa.miR.27b.5p
hsa.miR.22.5p
hsa.miR.491.5p
hsa.miR.1249.3p
hsa.miR.485.3p

(b)

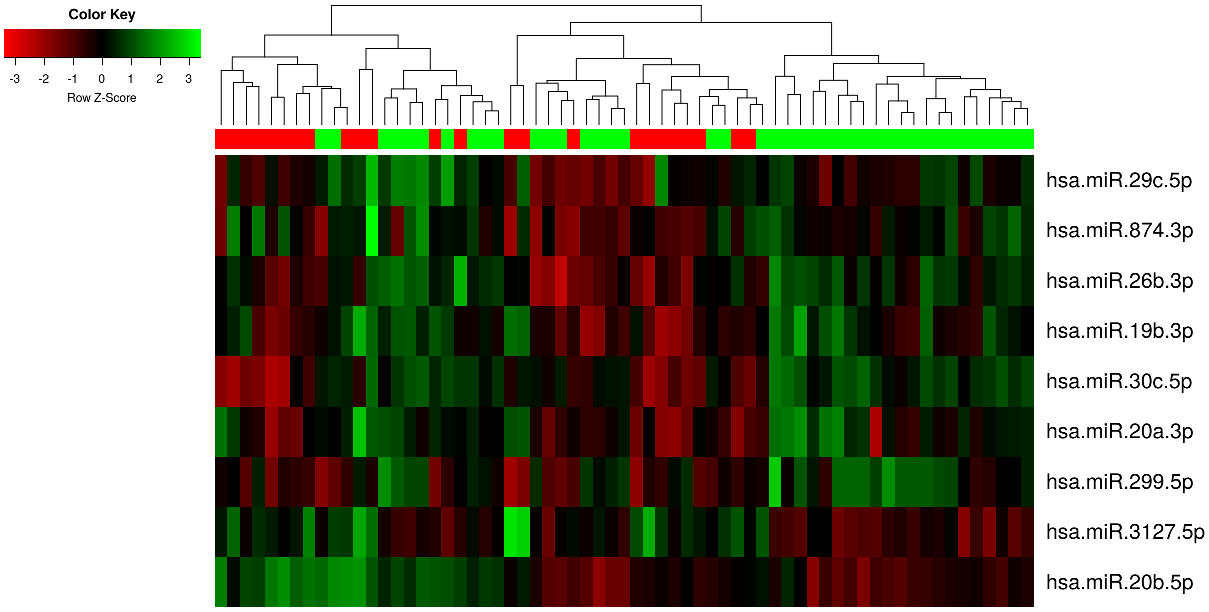

(c)

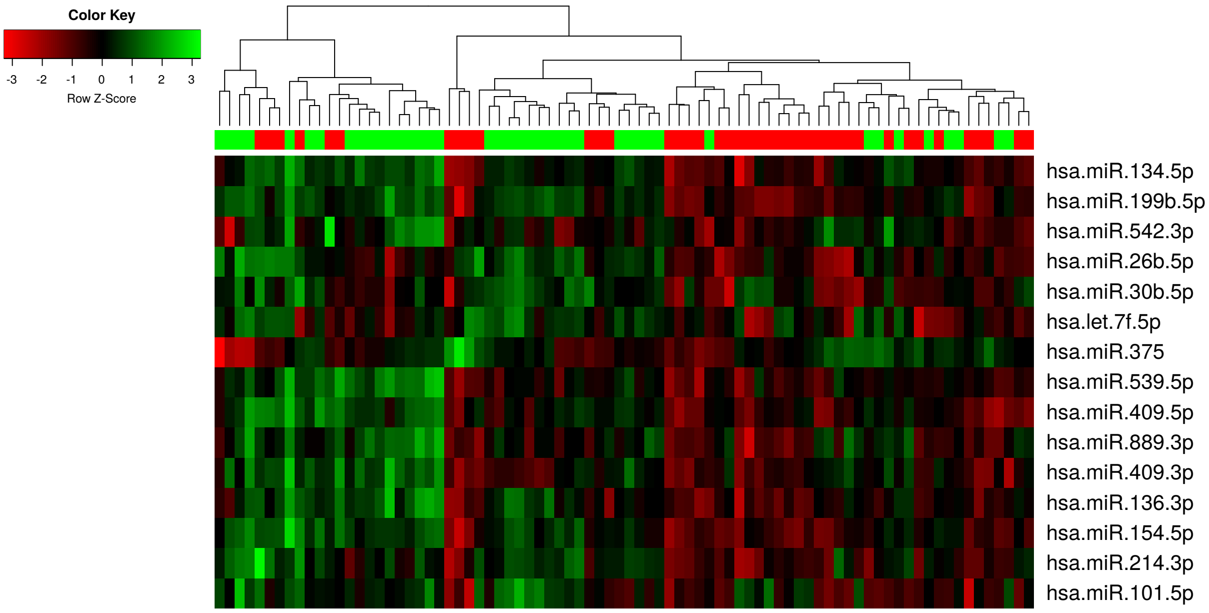

(d)

Figure S12: Clustering analysis of miRNA expression for (a) LA, (b) LB, (c) HER2-E and (d) BL subtypes with control patients. Color of the top bar represents group: red for cancer, green for control

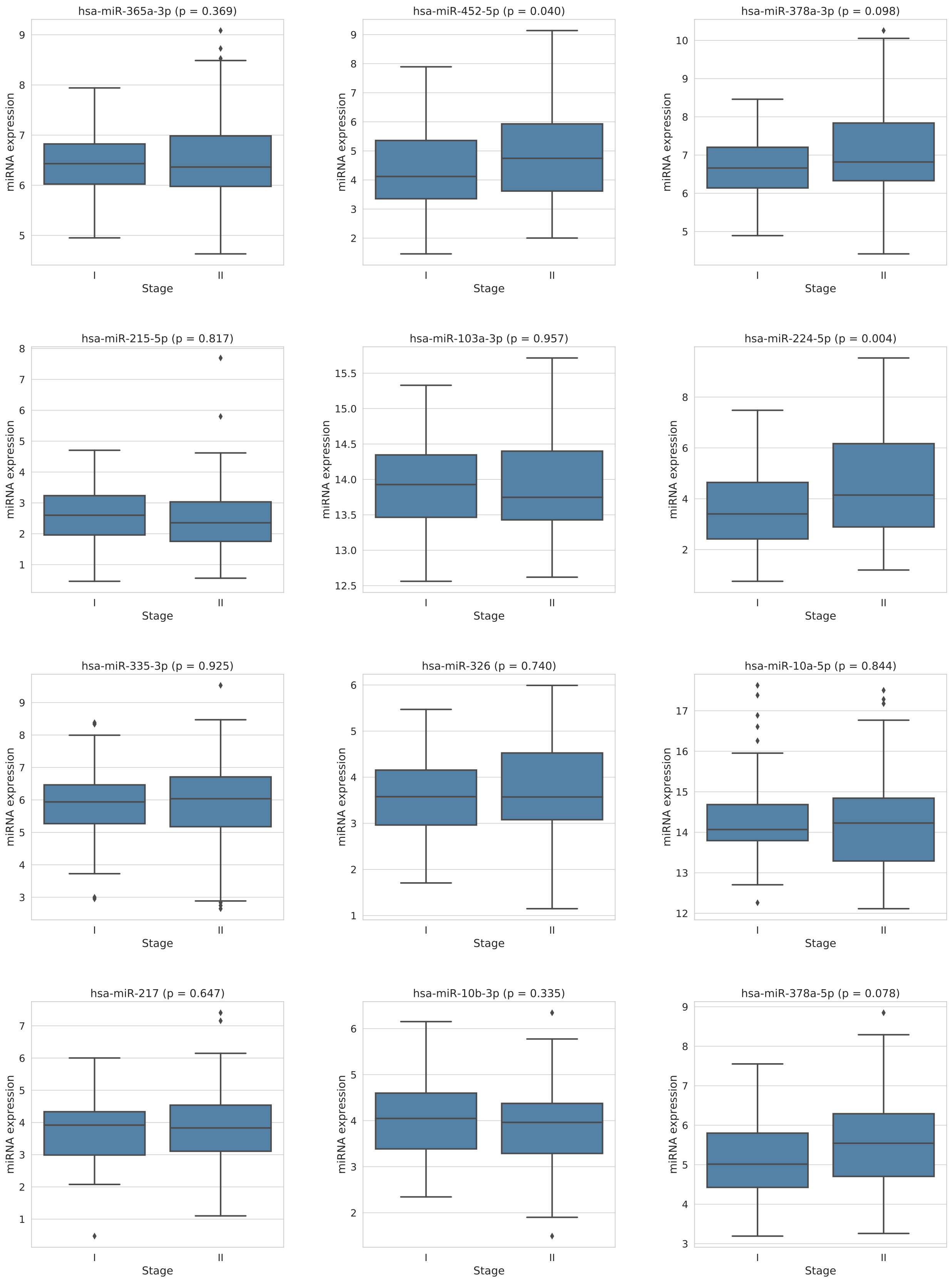
hsa-miR-365a-3p (p = 0.369)
hsa-miR-452-5p (p = 0.040)
hsa-miR-378a-3p (p = 0.098)
hsa-miR-215-5p (p = 0.817)
hsa-miR-103a-3p (p = 0.957)
hsa-miR-224-5p (p = 0.004)
hsa-miR-335-3p (p = 0.925)
hsa-miR-326 (p = 0.740)
hsa-miR-10a-5p (p = 0.844)
hsa-miR-217 (p = 0.647)
hsa-miR-10b-3p (p = 0.335)
hsa-miR-378a-5p (p = 0.078)
miRNA expression
Stage
I
II

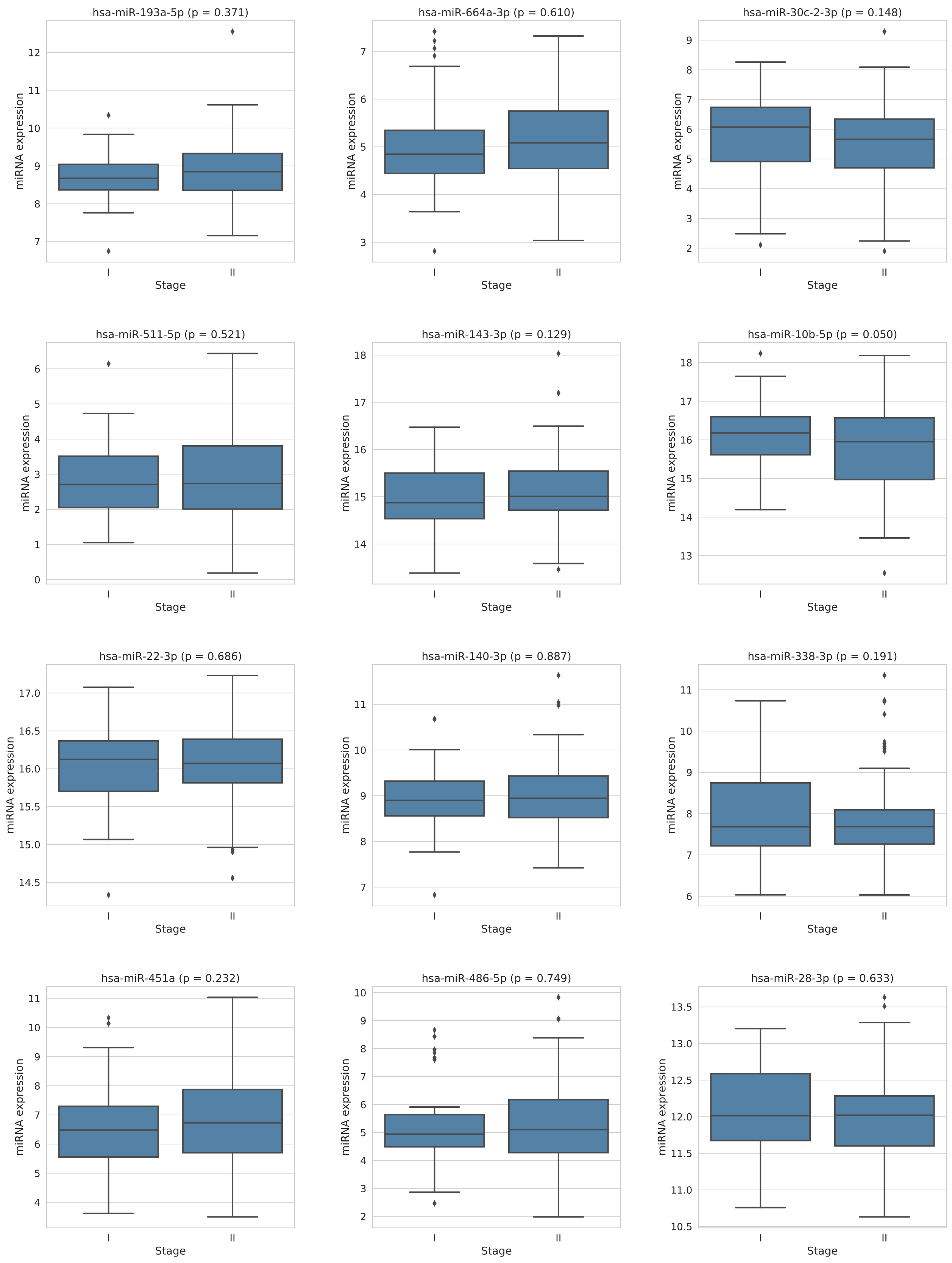
hsa-miR-193a-5p (p = 0.371)
hsa-miR-664a-3p (p = 0.610)
hsa-miR-30c-2-3p (p = 0.148)
hsa-miR-511-5p (p = 0.521)
hsa-miR-143-3p (p = 0.129)
hsa-miR-10b-5p (p = 0.050)
hsa-miR-22-3p (p = 0.686)
hsa-miR-140-3p (p = 0.887)
hsa-miR-338-3p (p = 0.191)
hsa-miR-451a (p = 0.232)
hsa-miR-486-5p (p = 0.749)
hsa-miR-28-3p (p = 0.633)
miRNA expression
Stage
I
II

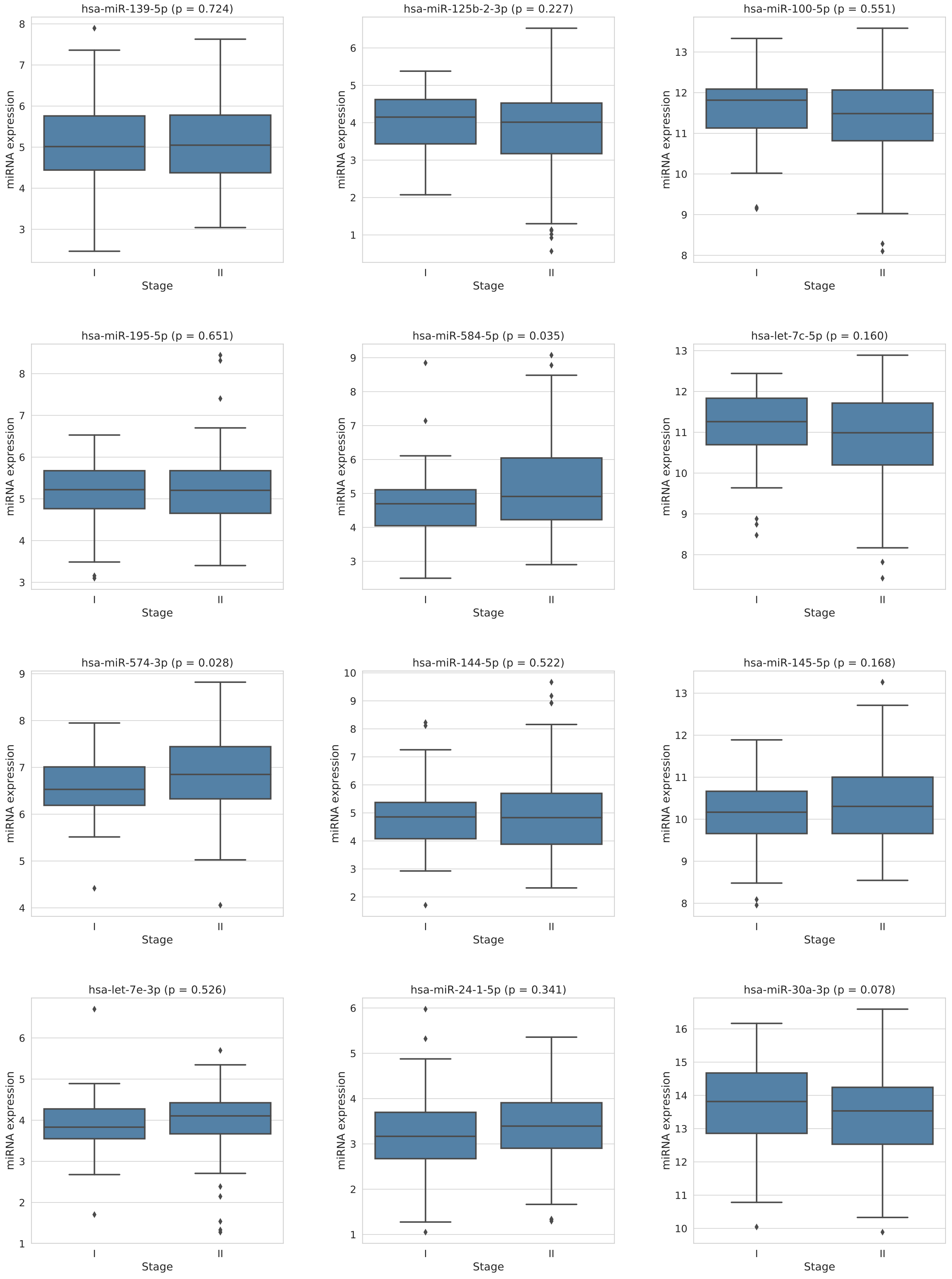
hsa-miR-139-5p (p = 0.724)
hsa-miR-125b-2-3p (p = 0.227)
hsa-miR-100-5p (p = 0.551)
hsa-miR-195-5p (p = 0.651)
hsa-miR-584-5p (p = 0.035)
hsa-let-7c-5p (p = 0.160)
hsa-miR-574-3p (p = 0.028)
hsa-miR-144-5p (p = 0.522)
hsa-miR-145-5p (p = 0.168)
hsa-let-7e-3p (p = 0.526)
hsa-miR-24-1-5p (p = 0.341)
hsa-miR-30a-3p (p = 0.078)
miRNA expression
Stage
I
II

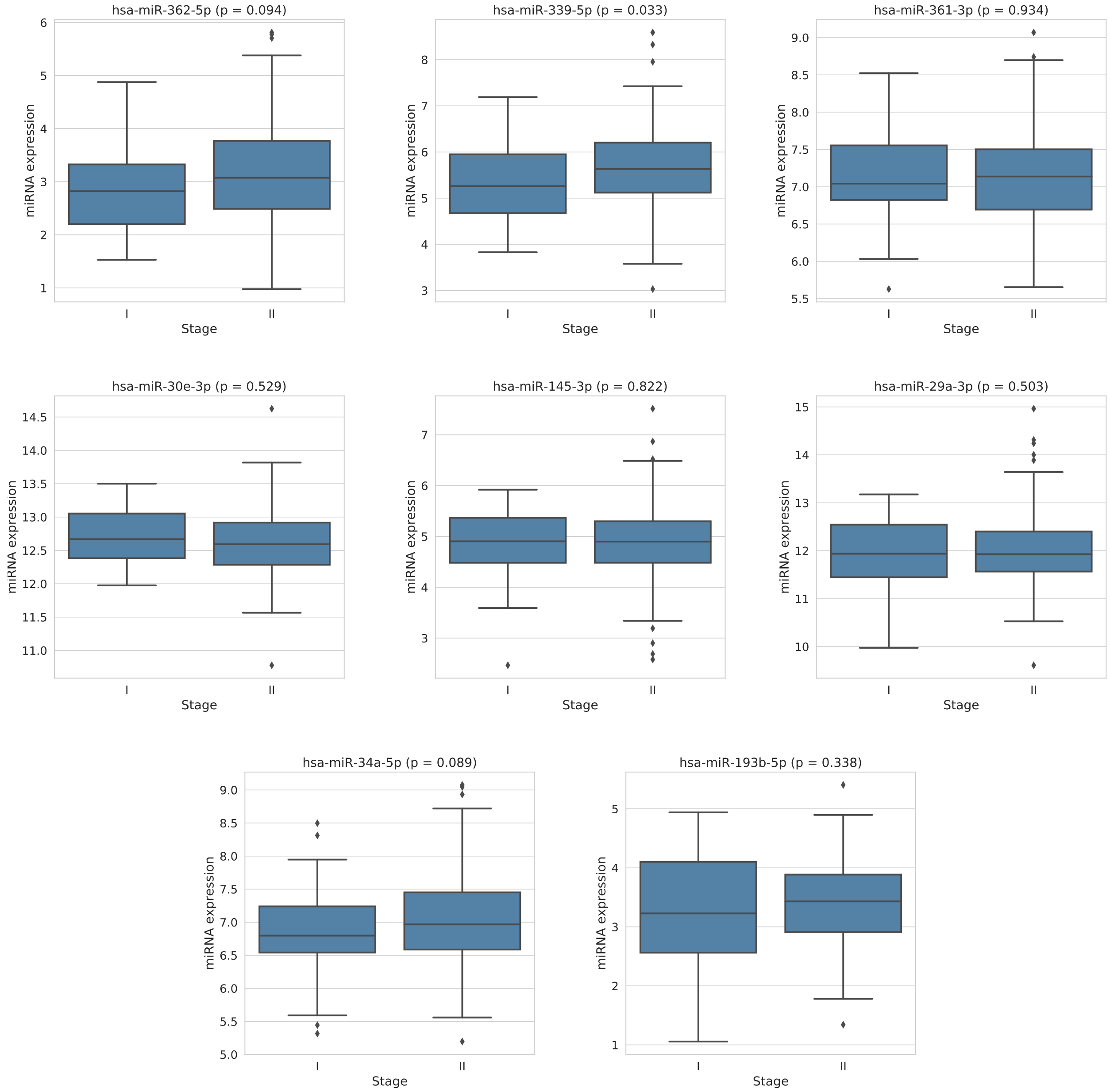
hsa-miR-362-5p (p = 0.094)
hsa-miR-339-5p (p = 0.033)
hsa-miR-361-3p (p = 0.934)
hsa-miR-30e-3p (p = 0.529)
hsa-miR-145-3p (p = 0.822)
hsa-miR-29a-3p (p = 0.503)
hsa-miR-34a-5p (p = 0.089)
hsa-miR-193b-5p (p = 0.338)
miRNA expression
Stage
I
II

(a)

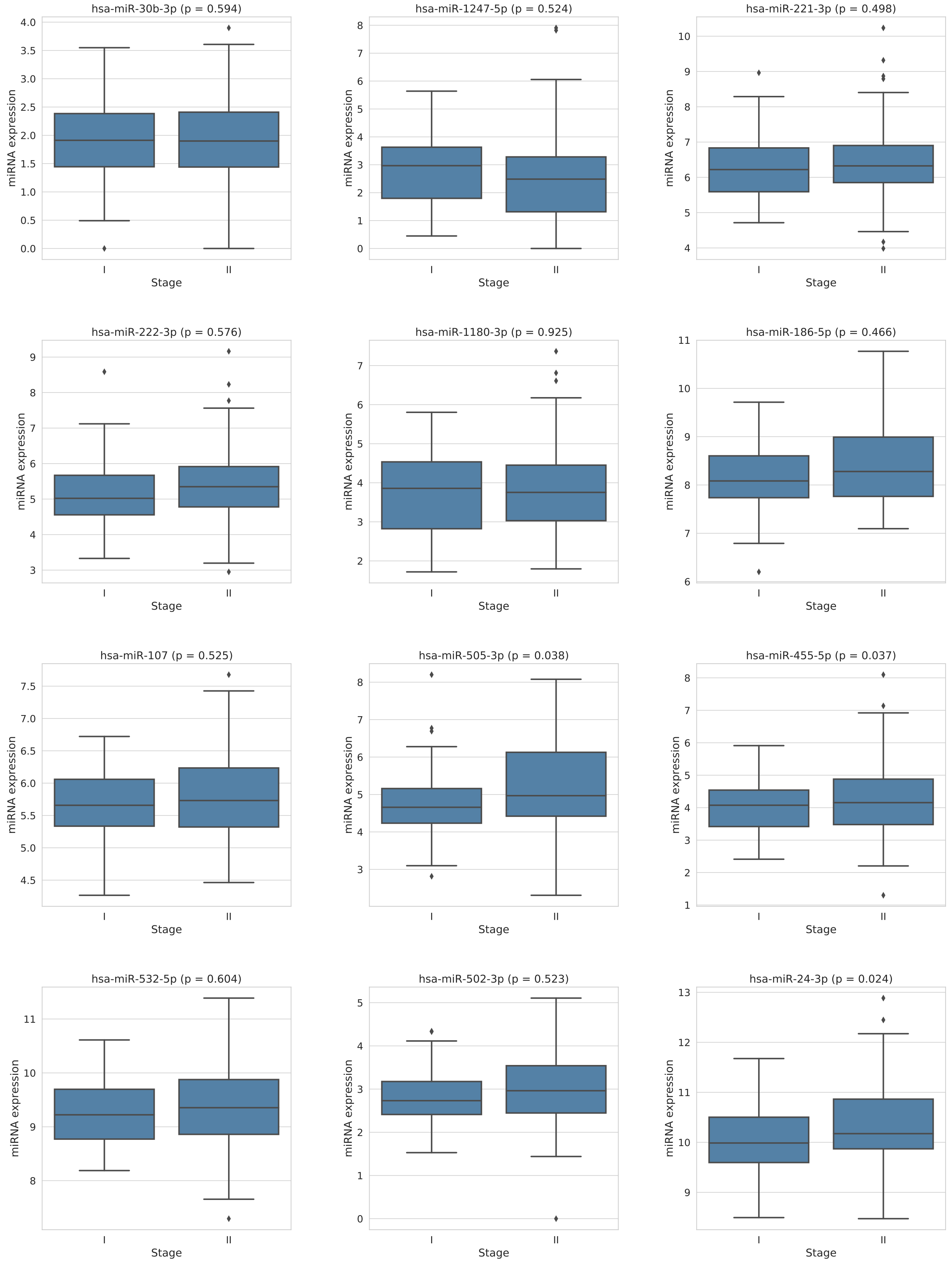
hsa-miR-30b-3p (p = 0.594)
hsa-miR-1247-5p (p = 0.524)
hsa-miR-221-3p (p = 0.498)
hsa-miR-222-3p (p = 0.576)
hsa-miR-1180-3p (p = 0.925)
hsa-miR-186-5p (p = 0.466)
hsa-miR-107 (p = 0.525)
hsa-miR-505-3p (p = 0.038)
hsa-miR-455-5p (p = 0.037)
hsa-miR-532-5p (p = 0.604)
hsa-miR-502-3p (p = 0.523)
hsa-miR-24-3p (p = 0.024)
miRNA expression
Stage
I
II

(b)

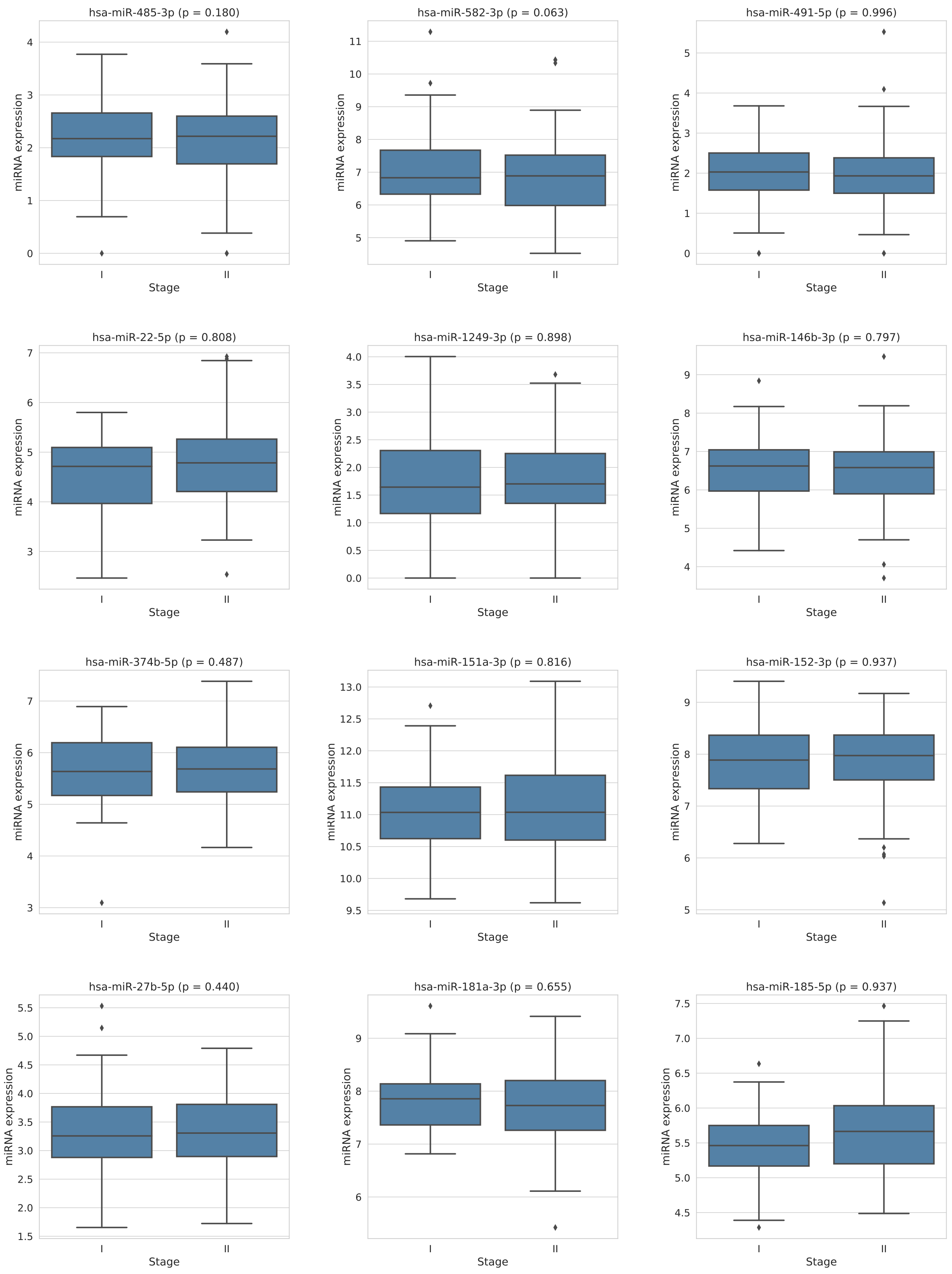
hsa-miR-485-3p (p = 0.180)
hsa-miR-582-3p (p = 0.063)
hsa-miR-491-5p (p = 0.996)
hsa-miR-22-5p (p = 0.808)
hsa-miR-1249-3p (p = 0.898)
hsa-miR-146b-3p (p = 0.797)
hsa-miR-374b-5p (p = 0.487)
hsa-miR-151a-3p (p = 0.816)
hsa-miR-152-3p (p = 0.937)
hsa-miR-27b-5p (p = 0.440)
hsa-miR-181a-3p (p = 0.655)
hsa-miR-185-5p (p = 0.937)
miRNA expression
Stage
I
II

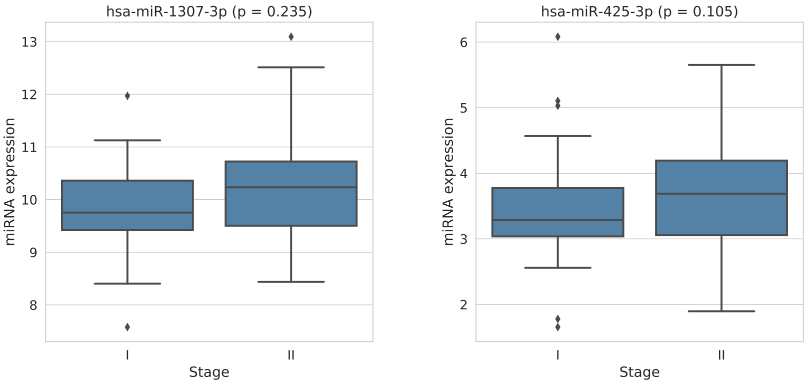
hsa-miR-1307-3p (p = 0.235)
miRNA expression
13
12
11
10
9
8
I
II
Stage
hsa-miR-425-3p (p = 0.105)
miRNA expression
6
5
4
3
2
I
II
Stage

(c)

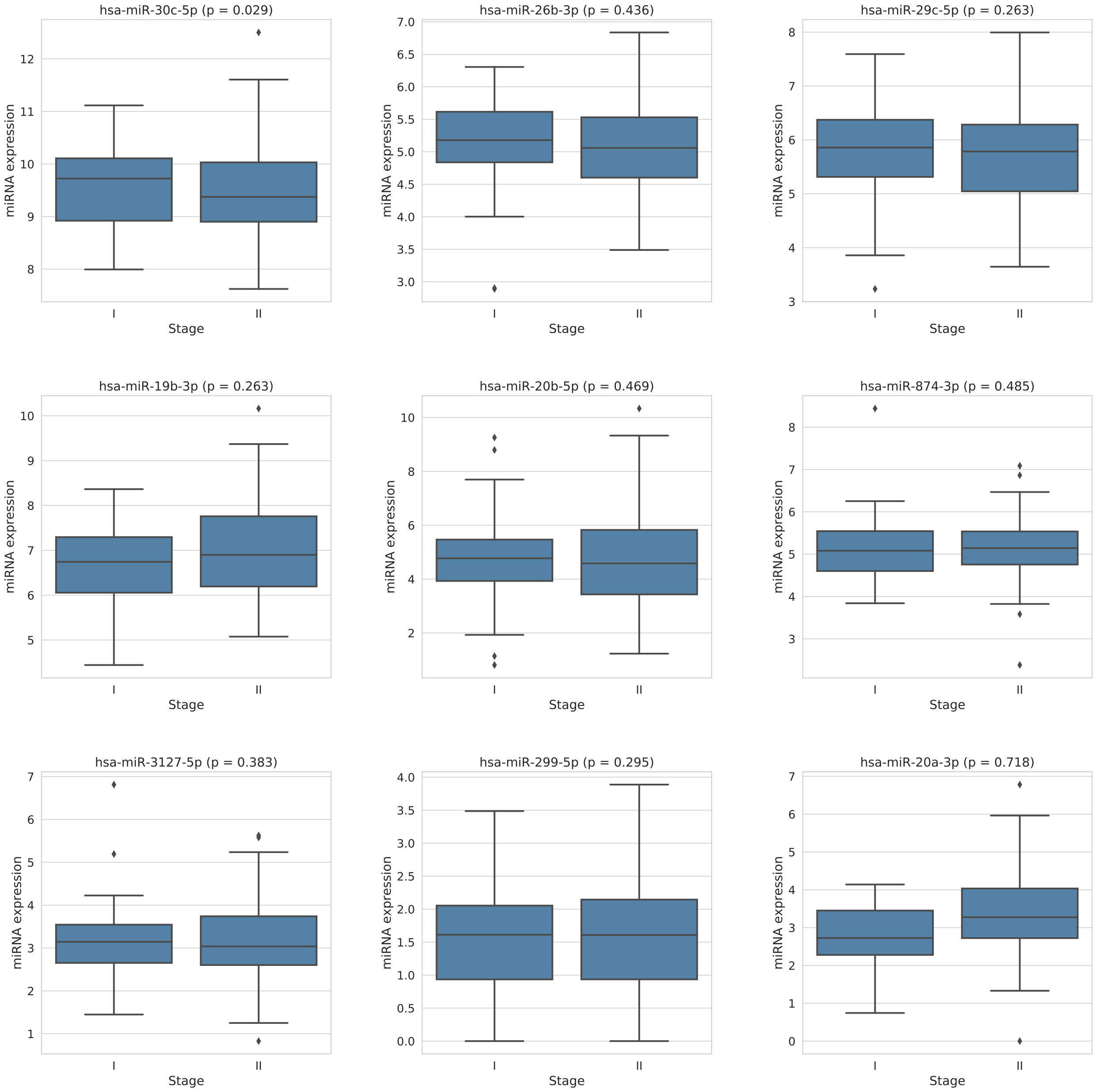
hsa-miR-30c-5p (p = 0.029)
hsa-miR-26b-3p (p = 0.436)
hsa-miR-29c-5p (p = 0.263)
hsa-miR-19b-3p (p = 0.263)
hsa-miR-20b-5p (p = 0.469)
hsa-miR-874-3p (p = 0.485)
hsa-miR-3127-5p (p = 0.383)
hsa-miR-299-5p (p = 0.295)
hsa-miR-20a-3p (p = 0.718)
miRNA expression
Stage
I
II

(d)

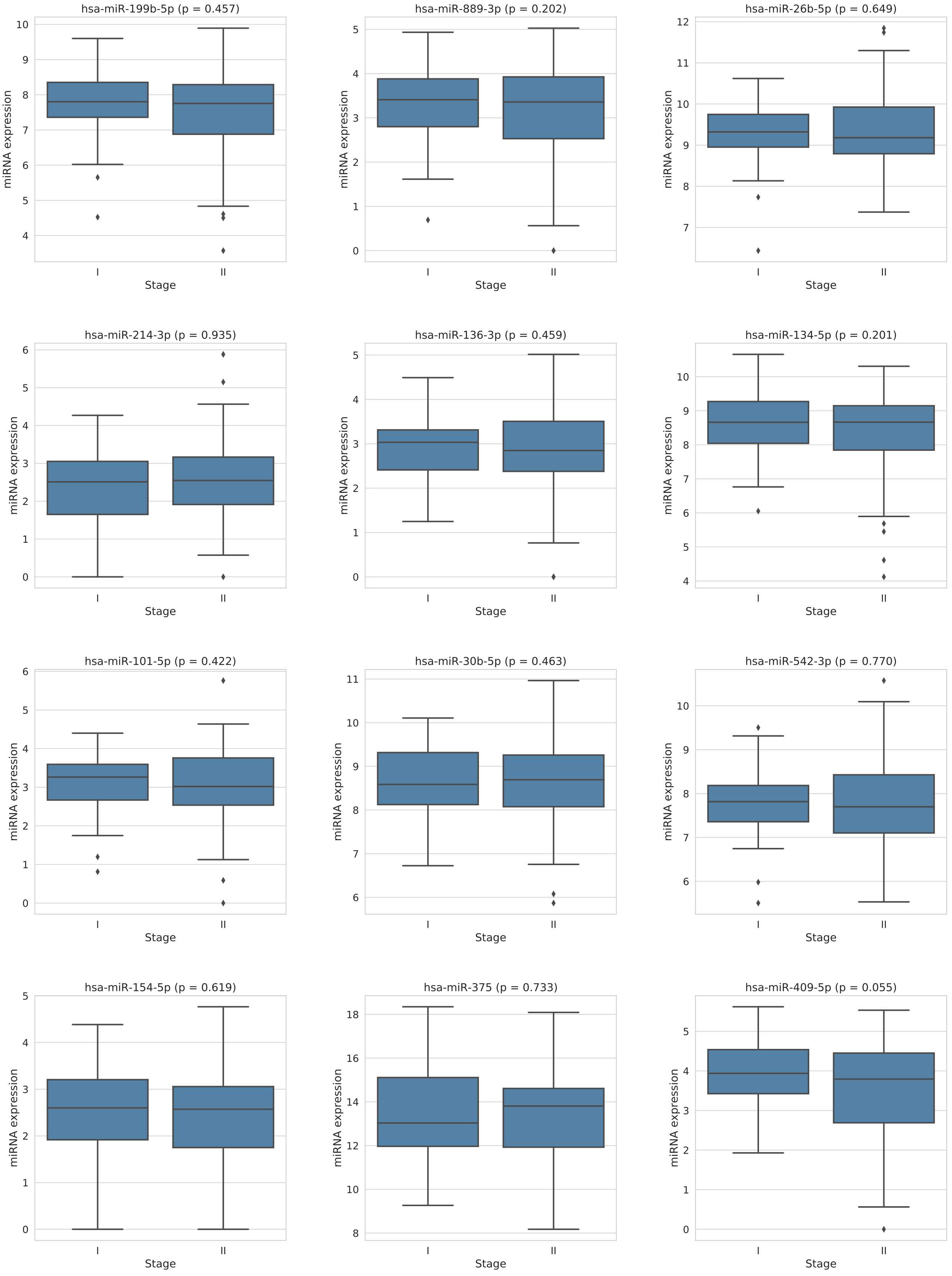
hsa-miR-199b-5p (p = 0.457)
hsa-miR-889-3p (p = 0.202)
hsa-miR-26b-5p (p = 0.649)
hsa-miR-214-3p (p = 0.935)
hsa-miR-136-3p (p = 0.459)
hsa-miR-134-5p (p = 0.201)
hsa-miR-101-5p (p = 0.422)
hsa-miR-30b-5p (p = 0.463)
hsa-miR-542-3p (p = 0.770)
hsa-miR-154-5p (p = 0.619)
hsa-miR-375 (p = 0.733)
hsa-miR-409-5p (p = 0.055)
miRNA expression
Stage
I
II

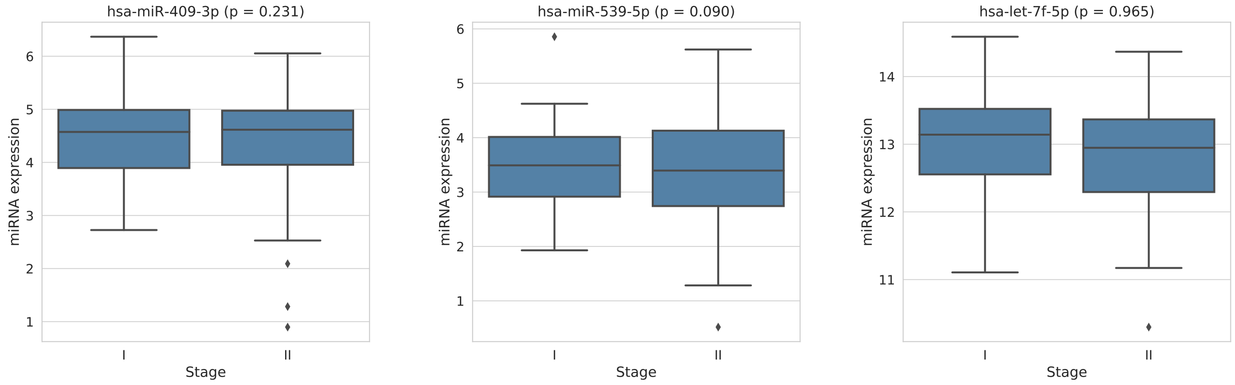

(e)

Figure S13: Boxplots showing the expression in cancer stages I and II for (a) 4-star miRNAs and 1-star miRNAs for (b) LA, (c) LB, (d) HER2-E and (e) BL

Figure S14: Regulatory networks of miRNAs (yellow rectangle), genes (purple ellipse) and transcription factors (blue octagon) for the 4-star miRNAs – full (a) and only showing loops (b), for the 4-star miRNAs

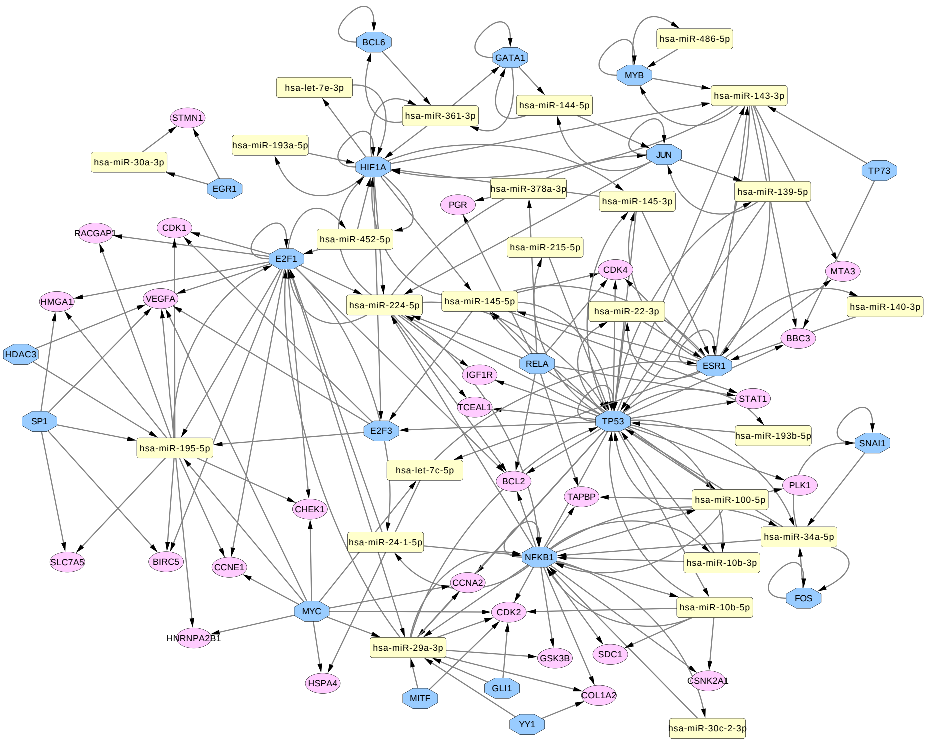

(a)

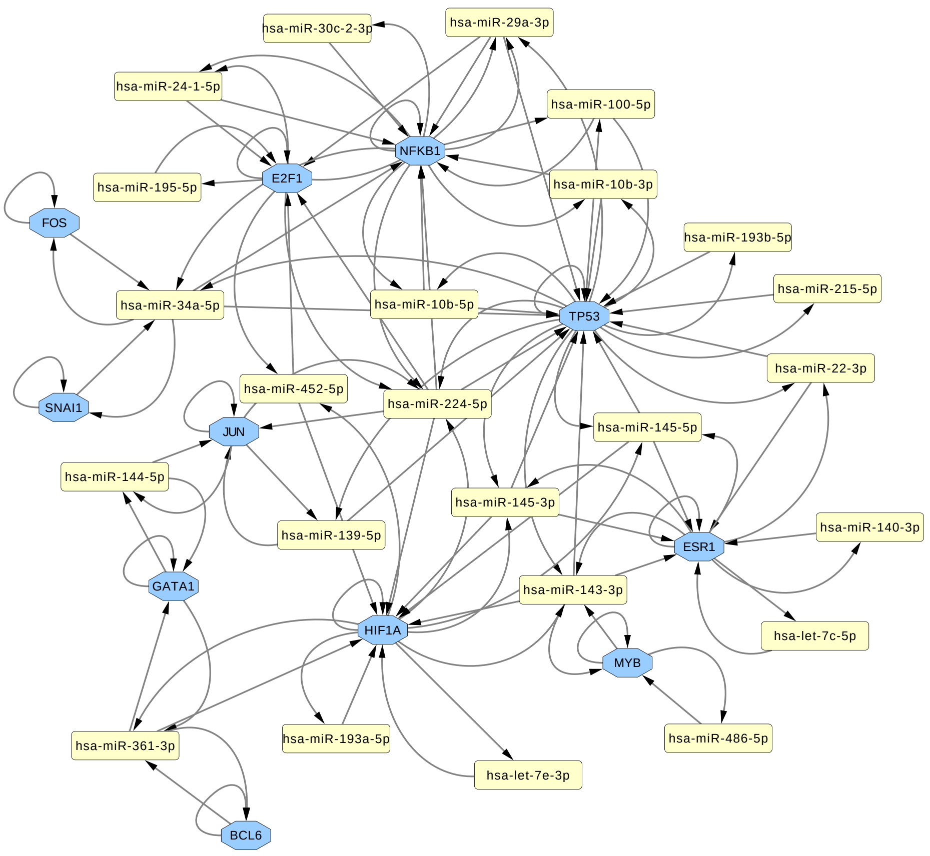

(b)

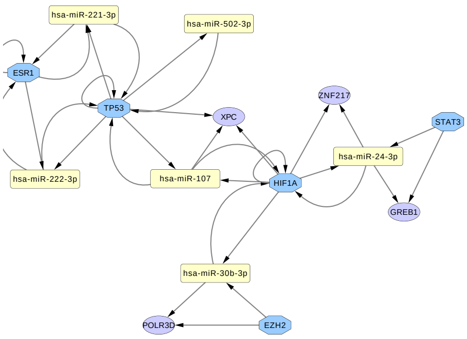
hsa-miR-221-3p
hsa-miR-502-3p
ESR1
TP53
XPC
ZNF217
STAT3
hsa-miR-24-3p
hsa-miR-222-3p
hsa-miR-107
HIF1A
GREB1
hsa-miR-30b-3p
POLR3D
EZH2

(a)

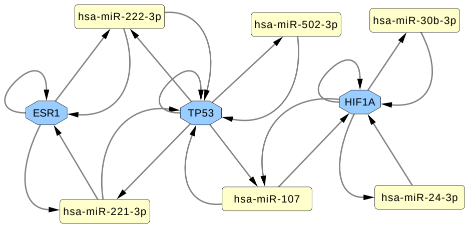
hsa-miR-222-3p
hsa-miR-502-3p
hsa-miR-30b-3p
ESR1
TP53
HIF1A
hsa-miR-221-3p
hsa-miR-107
hsa-miR-24-3p

(b)

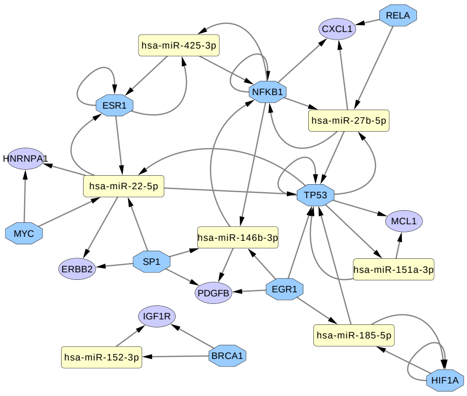
RELA
CXCL1
hsa-miR-425-3p
NFKB1
ESR1
hsa-miR-27b-5p
HNRNPA1
hsa-miR-22-5p
TP53
MCL1
MYC
hsa-miR-146b-3p
SP1
ERBB2
hsa-miR-151a-3p
PDGFB
EGR1
IGF1R
hsa-miR-185-5p
hsa-miR-152-3p
BRCA1
HIF1A

(c)

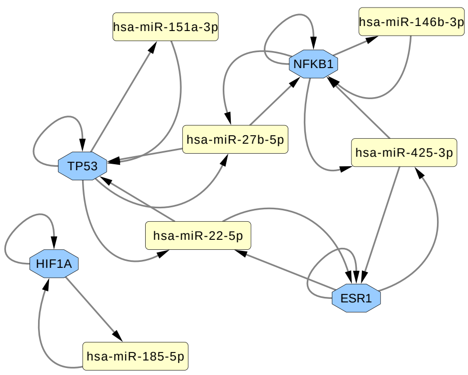
hsa-miR-151a-3p
hsa-miR-146b-3p
NFKB1
hsa-miR-27b-5p
TP53
hsa-miR-425-3p
hsa-miR-22-5p
HIF1A
ESR1
hsa-miR-185-5p

(d)

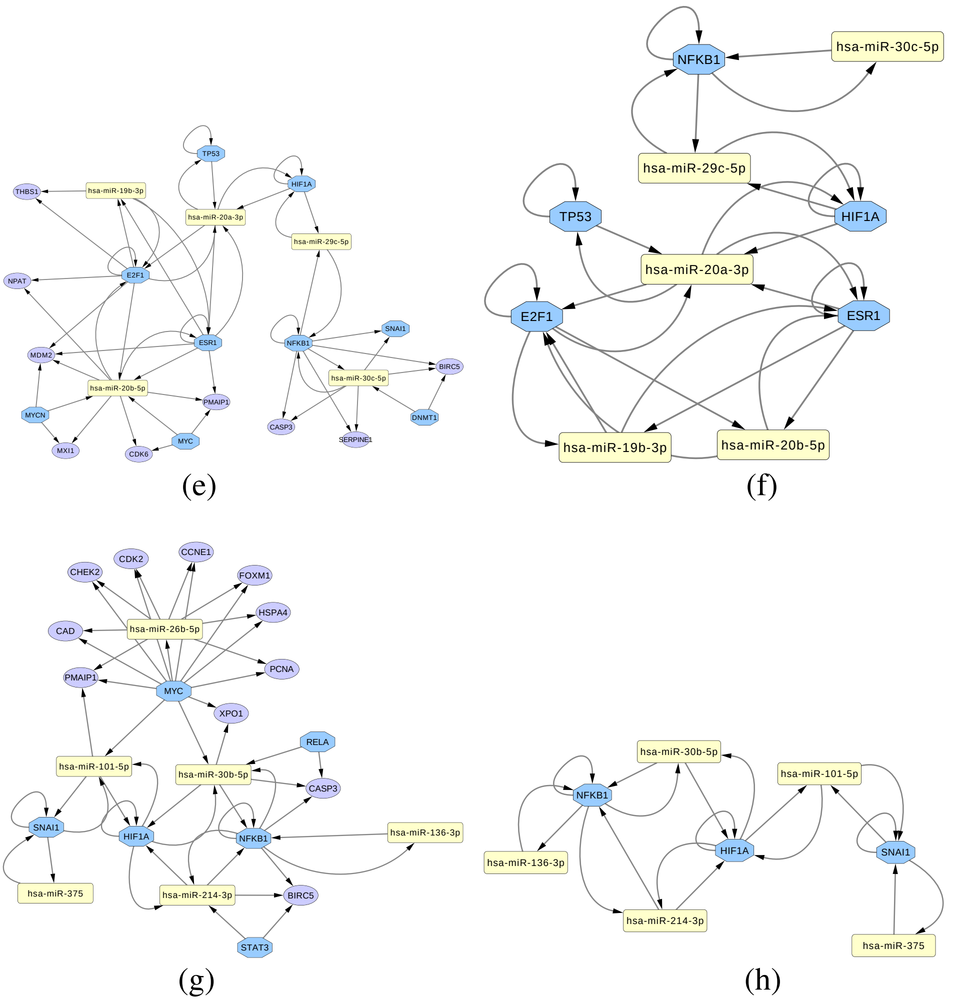

Figure S15: Regulatory networks of miRNAs (yellow rectangles), genes (purple ellipses) and transcription factors (blue octagons) for the 1-star miRNAs: LA (a, b), LB (c, d), HER2-E (e, f) and BL (g, h). Full networks are on the left and the ones only showing loops are on the right

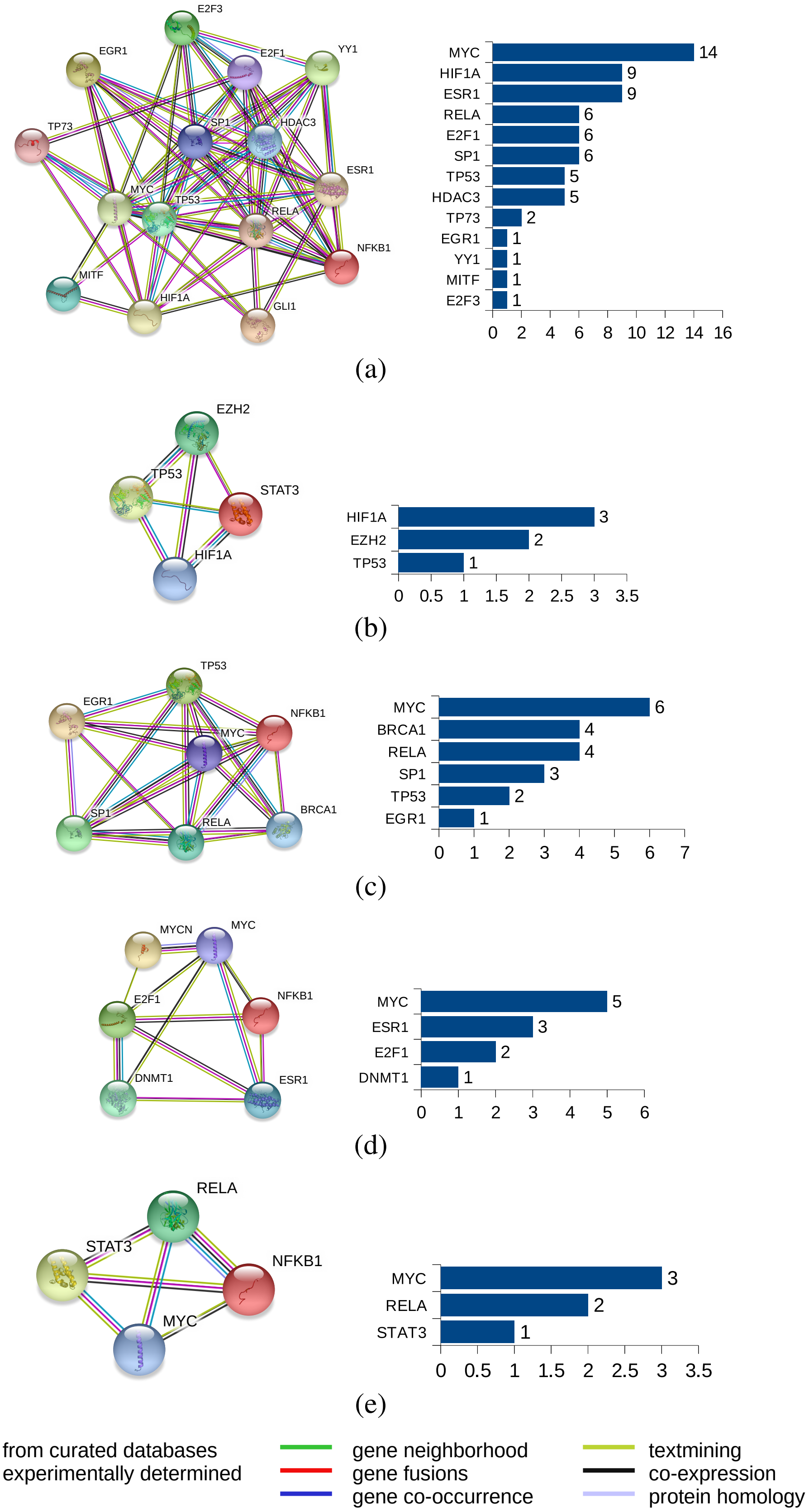

Figure S16: Protein-Protein Interaction networks for (a) 4-star and 1-star miRNAs for (b) LA, (c) LB, (d) HER2-E and (e) BL. The barplots show the degree (number of interactions) of each node in the Protein-Protein interaction networks. Colors of edges represent protein-protein association type, as indicated in the legend. Node colors are for aesthetic purposes
